# Sputtering onto liquids: a critical review

**DOI:** 10.3762/bjnano.13.2

**Published:** 2022-01-04

**Authors:** Anastasiya Sergievskaya, Adrien Chauvin, Stephanos Konstantinidis

**Affiliations:** 1 Plasma-Surface Interaction Chemistry (ChIPS), University of Mons, 23 Place du Parc, B-7000 Mons, Belgium; 2 Department of Condensed Matter Physics, Faculty of Mathematics and Physics, Charles University, Ke Karlovu 5, 121 16 Praha 2, Czech Republic

**Keywords:** low-pressure plasmas, magnetron, nanoparticles, nanoparticle formation, sputtering, sputtering onto liquids

## Abstract

Sputter deposition of atoms onto liquid substrates aims at producing colloidal dispersions of small monodisperse ultrapure nanoparticles (NPs). Since sputtering onto liquids combines the advantages of the physical vapor deposition technique and classical colloidal synthesis, the review contains chapters explaining the basics of (magnetron) sputter deposition and the formation of NPs in solution. This review article covers more than 132 papers published on this topic from 1996 to September 2021 and aims at providing a critical analysis of most of the reported data; we will address the influence of the sputtering parameters (sputter power, current, voltage, sputter time, working gas pressure, and the type of sputtering plasma) and host liquid properties (composition, temperature, viscosity, and surface tension) on the NP formation as well as a detailed overview of the properties and applications of the produced NPs.

## Introduction

**Figure 1:**
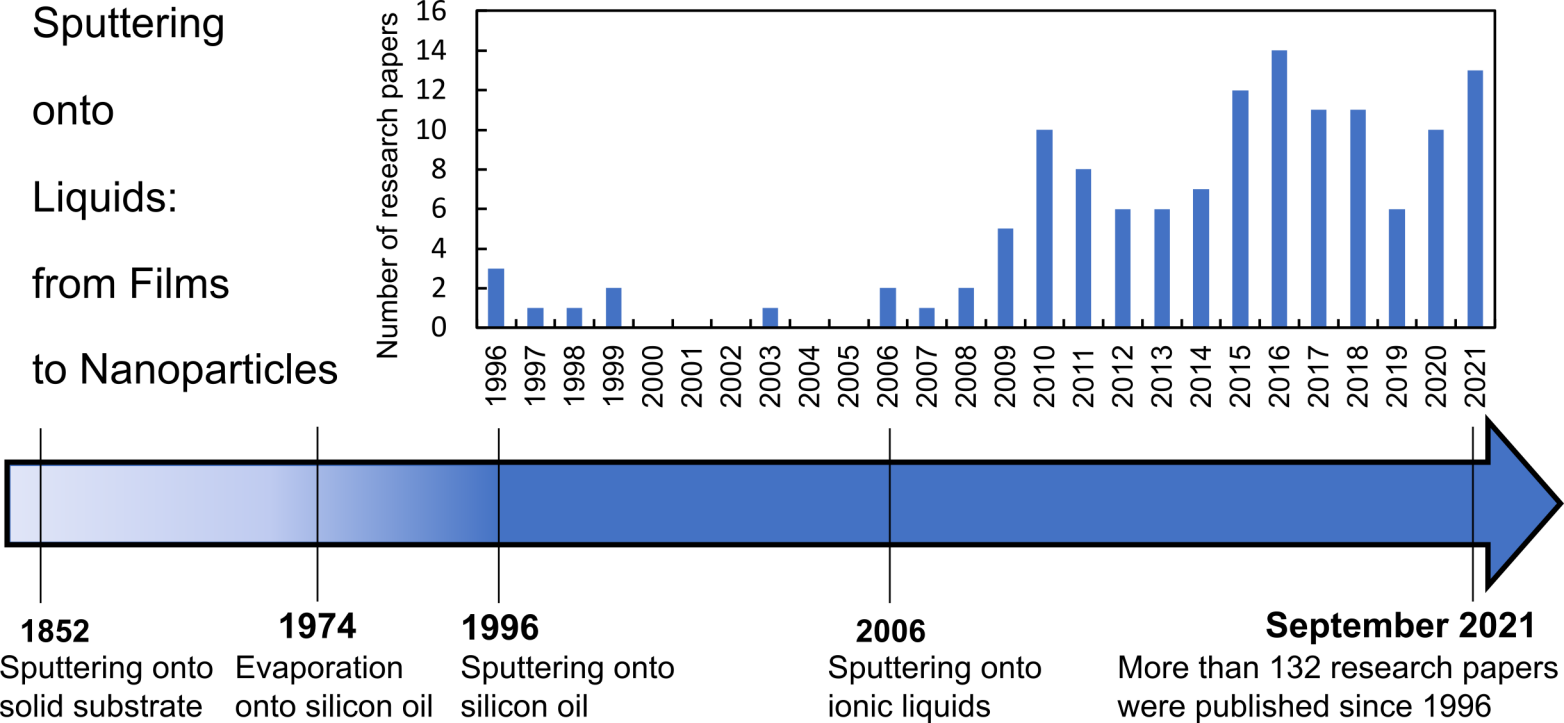
Growth of research interest in the SoL approach. Insert: Number of SoL-related publications in the period of 1996–2021.

According to the general terminology, nanoparticles (NPs) are objects that have a size of less than 100 nm. Because of the size effects, NPs have unique properties that allow them to be used as components of advanced materials for a wide range of applications such as optics, catalysis, or biomedicine [1–3]. Since the properties of NPs strongly depend on their size, size distribution, shape, composition, and the composition of surrounding media, it is extremely important to control these parameters. Nowadays, an interested reader can find thousands of different recipes of NP synthesis allowing one to prepare stable colloidal solutions of monodisperse NPs. However, not all published synthetic procedures are suitable for the upscaled production due to poor reproducibility or difficult purification processes [4–5]. Indeed, some applications require the usage of components with specific purity. Purifying the colloidal solutions from the by-products is an additional, time-consuming, and expensive step in the production chain that might cause detrimental issues, for example, NP aggregation. In this respect, low-pressure plasma-based sputtering onto liquids (SoL) is a relatively new synthetic approach in which the purification step can be avoided. This technique is based on the sputtering of metallic or ceramic targets over a host liquid substrate that sustains low pressures (typically in the range between 10^−4^ to 10^−3^ and 1–10 Pa). Because (i) the target material has a high purity and (ii) the host liquid plays the role of solvent and protective agent for growing NPs, the approach got a reputation of a method permitting the production of ultrapure monodisperse NPs without additional stabilizing and reducing reagents. Despite the fact that low-pressure plasma-based sputtering is known since 1852 and widely used in industry [6–8], the SoL approach is not a well-established field yet (Figure 1). In 1974, Yatsuya et al. used a liquid as a substrate during a physical vapor deposition (PVD) experiment. They thermally evaporated metals in vacuum onto silicon oil for NP production [9]. After the pioneering experiments of Yatsuya et al., depositions onto liquids were not mentioned in research papers for almost 20 years until 1996 when two research groups reported SoL experiments. Ye et al. studied the formation of silver films on the surface of silicon oil [10] while Wagener et al. prepared silver and aluminium NPs by deposition onto silicon oil using various experimental conditions [11]. Afterward, Torimoto et al. used a sputter coater for the synthesis of small gold (Au) NPs by sputtering an Au target onto a thin layer of an ionic liquid (IL) [12]. This paper [12] published in 2006 initiated a series of similar works in several labs which, were focused on the deposition of various metals onto low vapor pressure liquid substrates [13–14]. Careful analysis of the literature data has shown that about 132 research papers were published on the SoL topic since 1996 (Figure 1). It should be noted that, since the SoL field of research still does not have a well-established terminology, some works were found only by monitoring the citations of important pioneering works [10–12]. The next challenge after collecting the material was to make a direct comparison of the published data. Quite often, some essential experimental parameters were missing. These two problems come from the main feature of the SoL approach: It is the combination of a plasma-based technique and colloidal synthesis. Therefore, researchers from both communities are involved and physicists quite often do not pay enough attention to the solvent properties, while chemists may have little or no knowledge of plasmas and plasma–surface interactions. For this reason, we first briefly introduce the theoretical background of plasma-based sputtering and a short overview of the formation of metal NPs in liquid media.

This critical review aims to describe the scientific state of the art of SoL and to eliminate the misunderstanding of physicochemical basics of this technique. For this purpose, a presentation of the underlying processes leading to NP production is first provided. This part is constituted of a brief introduction to (i) sputtering and (ii) the kinetics of NP nucleation and growth in solutions. Then, the specific mechanism of NP production by SoL is discussed based on the detailed analysis of the effect of various experimental parameters on the size of NPs and their colloidal and oxidation stability in host liquid matrices. Furthermore, a carefully structured overview of all types of products produced by SoL is presented including monometallic and alloy NPs, oxide NPs, thin films, and nanocomposites and their promising optical, luminescence, and catalytic properties.

## Review

### 1 Theoretical background

#### 1.1 Techniques available for the synthesis of NPs

**Figure 2:**
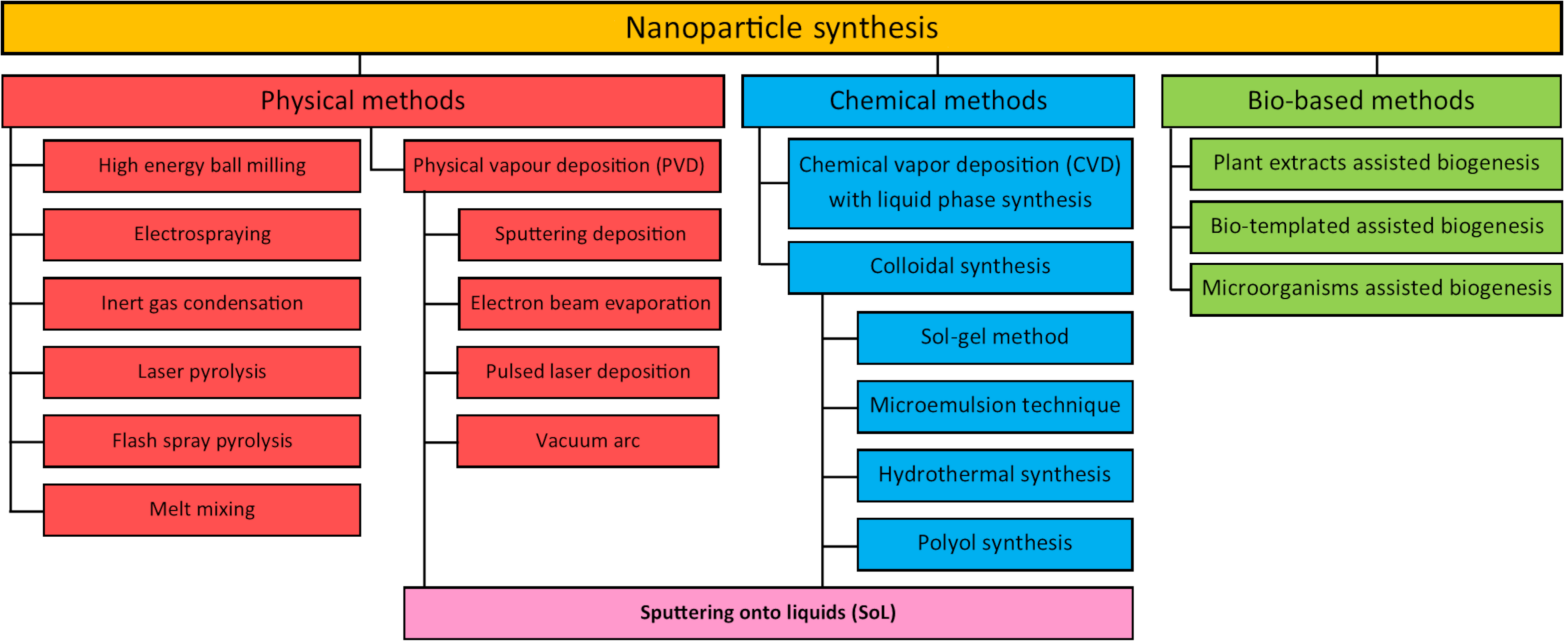
Summary of the different types of synthesis methods to produce NPs.

The mass production of NPs with controlled physicochemical characteristics is required for their applications in various fields on the industrial scale [15]. Generally, methods employed for the synthesis of NPs follow either the “top-down” or the “bottom-up” routes. On the one hand, in the “top-down” approach, a destructive technology is employed. The production starts from bulk materials that leach out systematically, leading to the generation of NPs. The starting material can be reduced in size using either a physical or a chemical route. On the other hand, the “bottom-up” approach, or self-assembly, refers to building up a structure atom-by-atom or molecule-by-molecule through a chemical or biological engineering procedure. In these synthesis approaches, the building blocks are initially formed and assembled into the final NPs. Therefore, NP synthesis methods can be divided into three groups: (i) physical methods, (ii) chemical methods, and (iii) bio-assisted methods [16–18]. An overview of the different approaches used in the literature for the synthesis of NPs is reported in Figure 2. For more detailed information on the methods and techniques available for the creation of NPs, dedicated reviews are available [16–21].

Physical methods are based on physical transformations of matter. These methods mainly operate with a “top-down” strategy where bulk materials are reduced in size, down to the nanoscale, via their interaction with photons, heat, or ions or via mechanical milling. Those methods are valuable as they are free from solvent contamination [16]. However, the production rate is relatively low, and the cost of production is very high, mainly due to the massive waste produced during the synthesis [22]. A further drawback is the large consumption of energy to maintain the required pressure and temperature conditions used in the synthesis procedures. The most common physical methods used to generate NPs are high-energy ball milling, laser ablation, electrospraying, inert gas condensation, PVD, laser pyrolysis, flash spray pyrolysis, and melt mixing [16].

Chemical methods are the traditional and most widely used approaches for the synthesis of colloidal NPs. In this case, molecular and/or atomic species are transformed into NPs. A typical procedure involves the growth of NPs in a liquid medium containing various reagents, particularly precursor species, reducing agents, and stabilizing agents to prevent the aggregation of NPs in the reaction mixture. Generally, the chemical methods are low-cost and allow one to produce large quantities of NPs; however, a couple of drawbacks can be highlighted and include contamination from precursor chemicals, use of solvents, and generation of sometimes hazardous by-products. These chemical methods can be divided into two major techniques, namely chemical vapor deposition (CVD) with liquid-phase synthesis and colloidal synthesis. In general, the colloidal synthesis of NPs is highly acclaimed due to its versatility [16].

So-called bio-assisted methods, biosynthesis, or green synthesis also attract the attention of many researchers due to the “environmentally friendly” nature of these processes, promoted by involving biological systems or by being related directly to biological systems [20,23]. These methods use, among others, bacteria, fungi, viruses, yeasts, and plant extracts to synthesize NPs. Although bio-assisted procedures are very promising, the major problem is the reproducibility of the processes. Besides, the exact mechanisms underlying the NP formation using green plant extracts have not been elucidated yet. Finally, their large-scale use is limited by the presence of undesired contaminants, such as fragments of biological materials, which require complicated, expensive, and time-consuming purification procedures. Bio-assisted methods can be divided into three categories according to the system used: (i) microorganisms, (ii) biomolecules, and (iii) plant extracts [23].

Besides bio-assisted methods, which are a promising approach but not well adapted for mass production yet, both chemical and physical processes have their advantages and can be complementary. On the one hand, chemical synthesis is very versatile in controlling NP size while the chemical purity of the NPs is limited mostly by the stabilizing agent used. On the other hand, physical methods are very clean methodologies to produce NPs, and the chemical purity of the materials produced is close to that of the starting bulk material. By combining both approaches, SoL takes advantage of both techniques and allows for the production of NPs with controlled size, shape, and purity [13].

The next parts of this section are dedicated to the detailed description of processes involved in the SoL process, namely the sputtering process and colloidal synthesis.

#### 1.2 Introduction to sputtering

**Figure 3:**
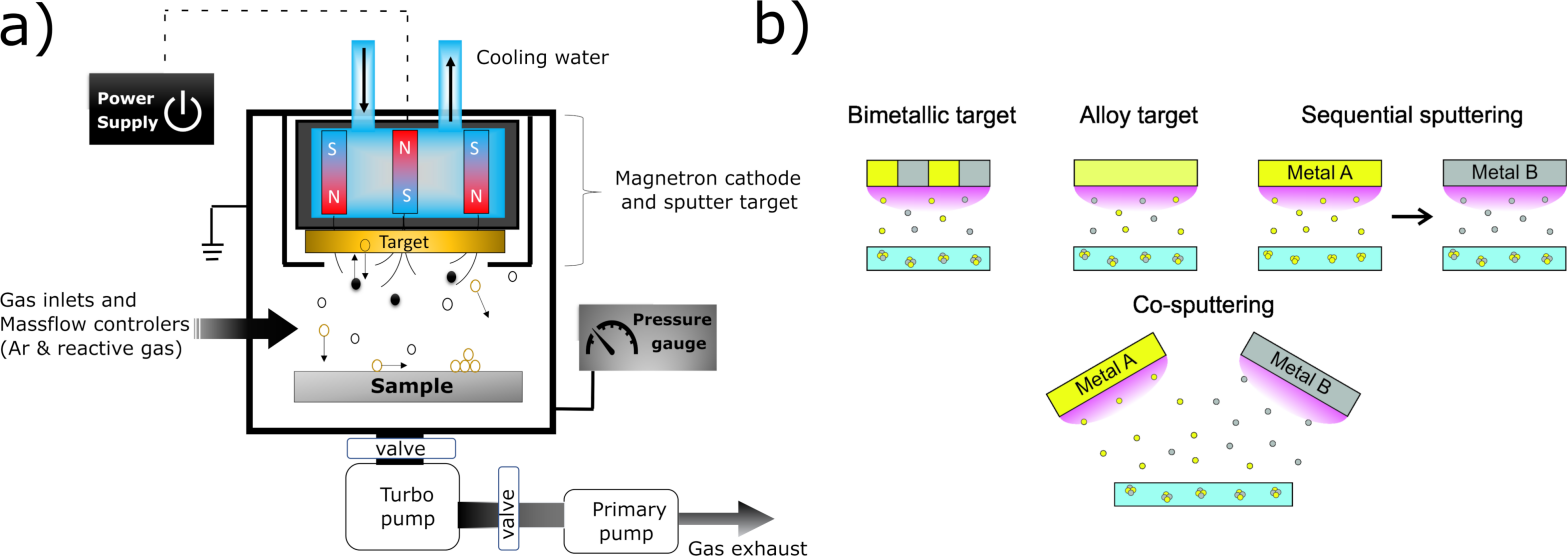
(a) Schematic representation of a (magnetron) sputtering deposition chamber along with some key setup components. Sputtered target atoms are represented in gold color while argon ions (Ar^+^) and argon neutrals (Ar) are shown as black and white dots, respectively. Plasma electrons are not shown. Some particles have their trajectories highlighted by black arrows. Magnetic field lines are drawn as dashed, arc-shaped, black lines in the vicinity of the target surface. The sample is a solid. (b) Four variations of the MS process for the deposition of multielement thin films. The deposition of a binary coating, containing elements A (yellow) and B (black), is presented. The substrate is a liquid.

In this chapter, we introduce the basic physical mechanisms involved in the sputter deposition of materials and related processes. Low-pressure plasma-based sputter deposition, along with evaporation, cathodic arc, or laser-based deposition, belongs to the family of PVD techniques, which have been originally designed to coat objects with thin film materials. Sputtering is the physical phenomenon describing the ejection of atoms from a surface bombarded by fast particles such as noble gas cations. These ions can be produced in a low-pressure plasma and accelerated towards a negatively biased surface, that is, the cathode of the system. A typical vacuum chamber used for coating deposition by sputtering is presented in Figure 3a where the key elements are presented: the negatively biased cathode covered by the target, that is, the source of atoms, the sample to be coated, the pumping system, the pressure gauge, the electric power supply, and the gas inlets.

Sputtering processes have been studied and developed for a long time [7–8] and have been implemented in the coating industry to deposit nanometer-scale films onto various solid substrates. Such functional coatings must have tailored physicochemical properties to fit the targeted application in fields such as mechanics, optics, electronics, and biomaterials. Various types of coatings can be produced, from pure metals to metal oxides, nitrides, carbides, oxynitrides to metal alloys, or chemically more complex combinations such as high-entropy alloys [24–25]. Also, the micro/nanostructure of the films is a very important aspect of the tailoring, and the sputtering process usually allows for controlling coating density, crystallinity, and micro/nanostructure to some extent by carefully varying the working parameters. For instance, energy transferred from the plasma to the film during growth through heating of the substrate and/or via bombardment by energetic particles or post-deposition annealing of the film can be envisaged. More information on thin film deposition (onto solid substrates) by sputtering-based processes can be found in books such as [26–31].

Commercial table-top sputter coaters can be found, for example, in electron microscopy facilities and are part of the sample preparation protocol. They are used to coat the sample with a conductive material to facilitate the observation. These devices usually come with a limited number of variable parameters. Customized vacuum chambers dedicated to the detailed study and development of this particular type of PVD process are also available in research laboratories. Sputtering has become industry-relevant since permanent magnets were set inside the cathode body, underneath the target, to generate a magnetic field in the target vicinity thus promoting magnetron sputtering (MS) cathode systems. Typical MS cathodes consist of a magnet placed at the center of the target and magnets with opposite poles on the target periphery. This configuration is schematically presented in Figure 3a, along with some typical variations (Figure 3b) that allow to further extend the possibilities towards the deposition of multielemental coatings. In Figure 3b, the deposition of elements A and B is depicted but the deposition processes can be further upgraded to include three cathodes (or more) or to perform co-sputtering with two alloy targets to deposit coatings containing, for instance, four different elements. Industrial-sized coaters, especially in the optical coating industry, rely on several meter-long rotating cylindrical targets [32].

In MS configuration, the combined magnetic and electric fields, the latter being generated by the negative voltage applied to the cathode, allow for trapping the plasma electrons near the target surface to enhance gas ionization in that region. Consequently, the ion flux towards the target and the corresponding cathode current is significantly increased as compared to a diode glow discharge ignited without inserting magnets. In the case of MS systems, the discharge current *I* scales exponentially with the applied voltage *V,* as highlighted by the equation *I* = *k*·*V^n^*, where *k* and *n* depend on the magnetron cathode and other process parameters as discussed in [33–34] while *n* is an indication of the electron-trapping efficiency.

Since the discharge current is dramatically increased because the ionization efficiency of the gas is augmented, the gas pressure can be substantially decreased, typically in the millitorr (roughly pascal) range, as compared to conventional diode discharges. In turn, lowering the working pressure while keeping a high discharge current density, that is, a few tens of millamperes per square centimeter, allows for increasing the target erosion rate and facilitates the transport of the sputtered atoms towards the substrate as gas-phase scattering is minimized. The film deposition rate, lying typically in the range of several nanometers per minute, is therefore higher compared to non-magnetized sputtering discharges. Here a remark should be made: While the film growth rate is usually expressed in units of thickness per unit time (e.g., nanometers per minute) in sputtering-related publications, in the case of SoL, it might be better to present the deposition rate as a flux of particles or a mass deposited onto the liquid surface per unit time.

**Figure 4:**
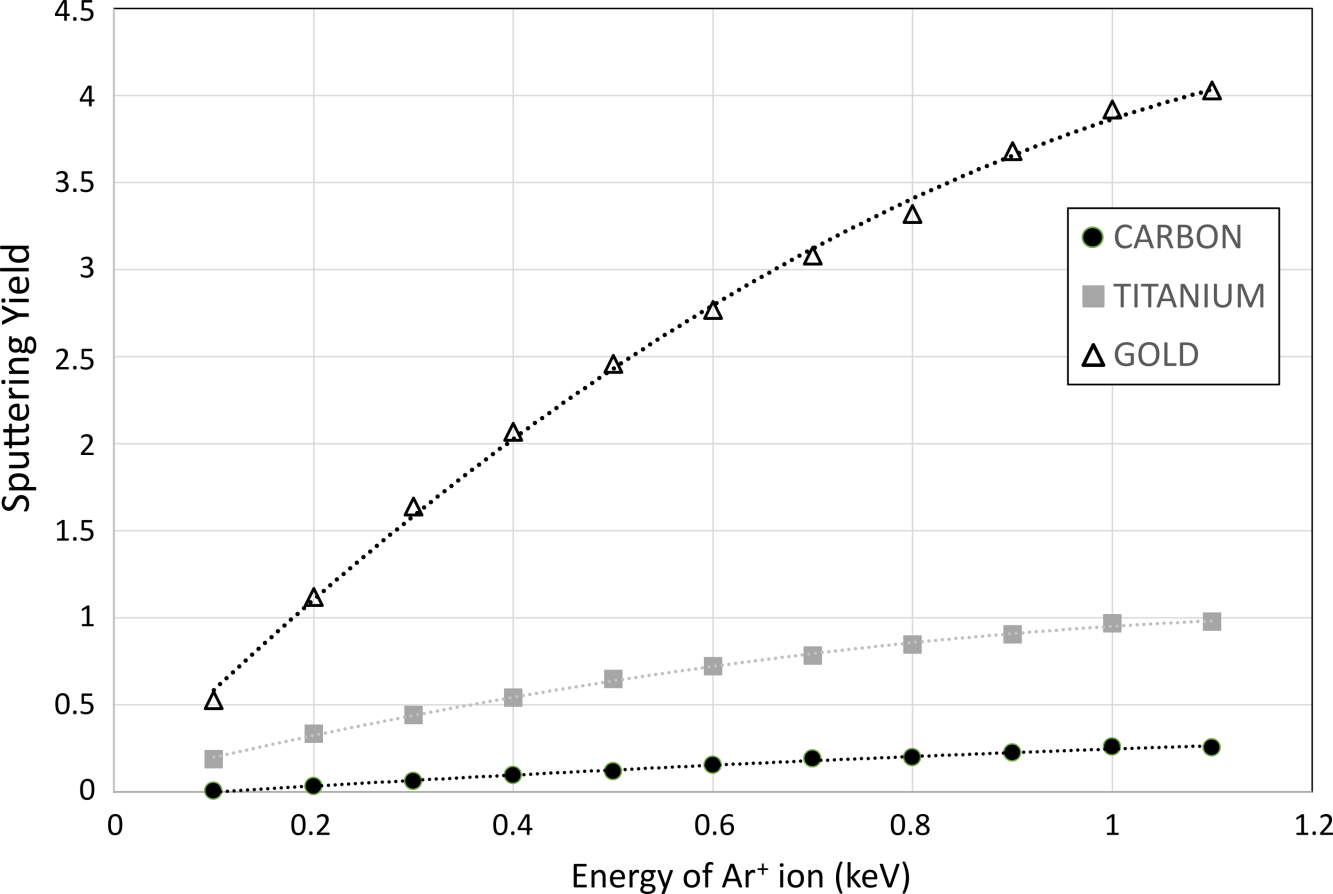
SRIM calculation of the sputtering yield of C (black disks), Ti (grey squares), and Au (white triangles) targets by argon ions. The lines are a guide to the eye.

The sputtering process itself is characterized by the so-called ion-induced sputtering yield, which represents the probability of sputtering a given number of target atoms for one incident plasma ion. The plasma ion (e.g., Ar^+^), accelerated towards the surface of the sputter target by the negative potential applied to the cathode, transfers its momentum to the surface atoms, which are expelled out of the surface, that is, sputtered. More information about the theory of ion-induced sputtering can be found in [8]. Ion–surface interaction and sputtering yield data can be calculated using codes such as SRIM [35] and TRYDIN [36], while transport of the sputtered species through the gas phase and subsequent film growth can be computed using, for example, SIMTRA [37] and NASCAM [38] codes, respectively. The evolution of the sputtering yield calculated by SRIM for carbon (C), titanium (Ti), and Au targets as a function of the kinetic energy of the bombarding argon ions is presented in Figure 4. The kinetic energy of the argon ions ranges from 100 to 1100 eV, which are typical values for MS discharges. For the calculation, ions are assumed to impinge the surface at normal incidence and the thickness of the target is set to 1 µm. The sputtering yield values are averaged over 5000 ion impacts. The sputtering yield is influenced by the surface binding energy (*E*_b_) of the target material, here *E*_b_ = 7.41, 4.89, and 3.8 eV for carbon, titanium, and gold, respectively. These values are provided by the SRIM code.

Atoms leaving the target surface have an average kinetic energy of the order of a few electronvolts [39–41]. This is a higher value than atoms produced during evaporation-based processes, whose energy is in the range of the thermal energy of the body that is heated and evaporated, that is, a fraction of an electronvolt. Sputtered atoms may collide with plasma atoms and lose their energy before reaching the neighboring surfaces onto which they condense. The collision rate and the fraction of the kinetic energy they dissipate in the gas phase mainly depend on the product of pressure times the distance traveled. Typically, the pressure lies in the range of pascals while the distance between the target and the substrate is of the order of several centimeters. Ultimately, these atoms may be thermalized if the number of collisions is high and the atoms land on the surface with a kinetic energy of the order of the gas temperature (ca. 1/40 eV). Condensation enthalpy is released as atoms land on the surface and heat the substrate. Besides condensation, energy is also provided to the surface by other plasma species such as ions, electrons, (fast) neutrals, and photons whose fluxes may vary in accordance with the process conditions [42]. It should be mentioned that the surface of the sputter target, whose temperature is gradually increasing because of ion bombardment, emits IR photons. This radiation is detected at the substrate location and contributes to an increase of the substrate surface temperature as well [43]. In the case of reactive sputtering (discussed below), the formation of chemical bonds between, for instance, oxygen and deposited metal atoms, may occur and contribute to the increase of the amount of heat released on the surface, too. Thornton reported a substrate temperature in the range of 150 °C when measuring heat fluxes during magnetron sputter deposition processes [44]. In Thornton’s report, the probe used for the heat flux measurement, whose temperature increases when submitted to the sputtering plasma, was a stainless-steel body weighing 200 mg. Ultimately, in the case of a solid substrate, the heat transfer and resulting increase of surface temperature will influence the film growth mechanism and coating properties such as microstructure and phase constitution, as emphasized in [45–46]. Depending on the process conditions, energy flux values span from a few tens to thousands of milliwatts per square centimeter at the substrate position [42,47]. In the case of the SoL process, the abovementioned contributions may also impact the liquid temperature but also its physicochemical properties as plasma electrons, ions, and radiation may induce, for example, bond breaking in the liquid molecules.

**Figure 5:**
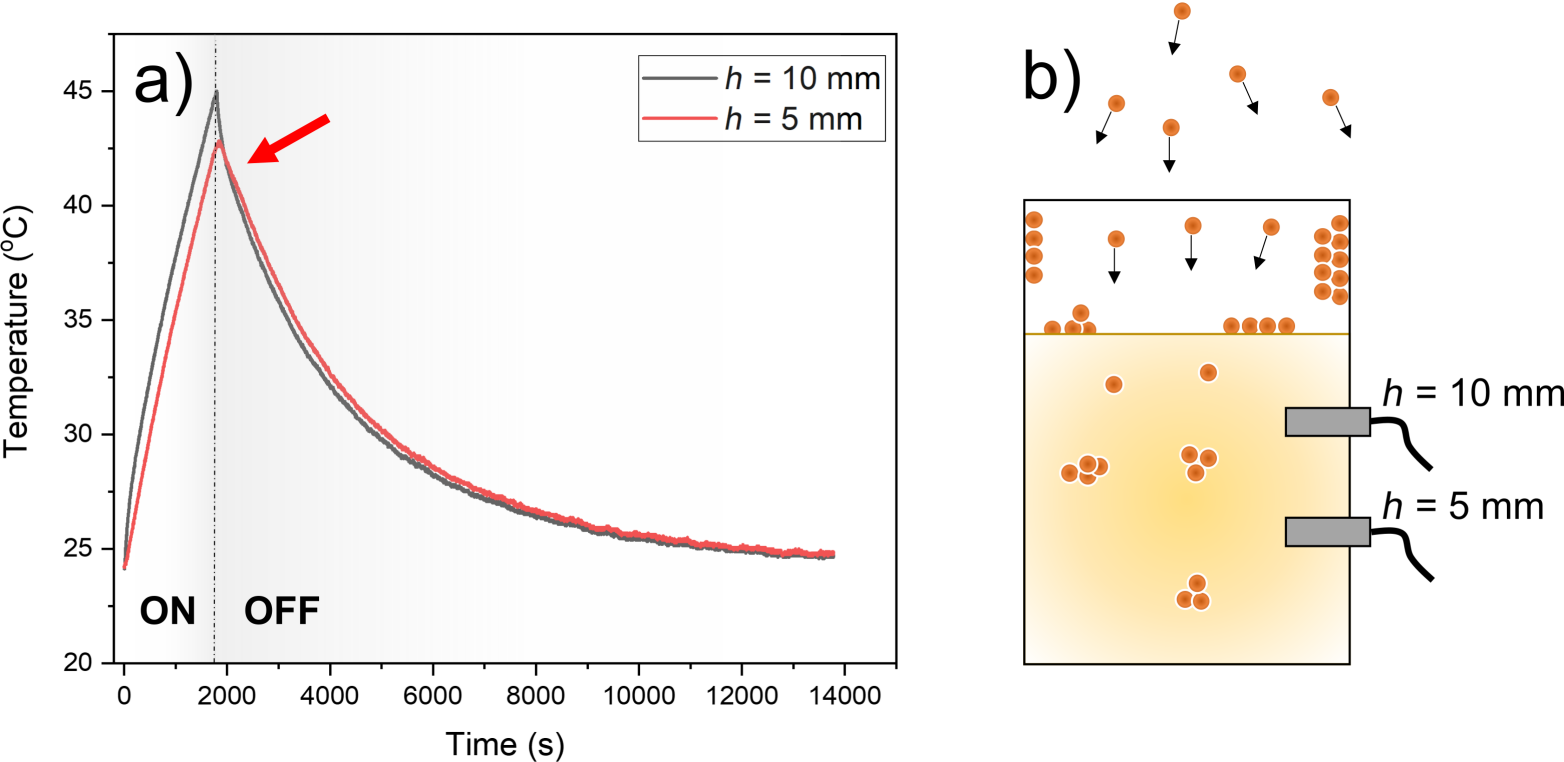
(a) Evolution of the castor oil (4 g) temperature as a function of time, at 5 and 10 mm from the bottom of the container. Copper is sputtered by applying 4 W/cm^2^ to the cathode. Argon pressure equals 0.07 Pa. The topmost probe records a higher temperature than the probe located deeper inside the liquid during the plasma ON time. The red arrow highlights the presence of a cross-over point during the cooling period, that is, plasma OFF, when the temperature gradient is reversed. (b) Schematic drawing (not to scale) showing the positions of the two probes inside the container. Sputtered copper atoms are shown as well.

In Figure 5, we report some of our experimental data regarding the heating of the host liquid when the latter is submitted to a MS plasma [48]. The temperature of 4 mL of castor oil was recorded by inserting two thermocouples at two different positions inside the cup (3 cm in height and 1.25 cm in radius) containing the liquid, during the plasma ON and OFF times. The liquid is heated by the sputtering plasma and cooled by the emission of radiation. Values of the liquid temperature reached after 1800 s of plasma ON time are reported in [48] for a power density of 4 W/cm^2^ applied to the magnetron cathode. The argon plasma (0.07 Pa) is used to sputter a 5 cm in diameter copper target. The liquid is placed 10 cm away from the target. The red arrow on the thermograms highlights the presence of a cross-over point when the temperature gradient is reversed during the cooling period (plasma OFF). The heating of the liquid as well as the presence of a temperature gradient might influence the physicochemical properties of the host liquid and the transport of matter inside the liquid medium.

It is possible to sputter two or more elements simultaneously by setting up the sputtering apparatuses identical to the one presented in Figure 3 with segmented, alloy, or pressed powder-based targets [49–50]. However, one should pay attention to the fact that the elemental composition of the deposited material might not be identical to the elemental composition of the sputter target. Various phenomena may intervene here such as a difference in the scattering efficiency for the various sputtered atoms transported in the gas phase and different sputter yields, ultimately leading to the abovementioned composition discrepancies between the source and the deposited material. The interested reader might refer to articles that exemplify this discussion such as [51–53]. On the other hand, co-sputtering processes involve two or more cathodes inside the vacuum chamber; the electrical power applied to each cathode independently controls the flux of each sputtered atoms and, hence, the chemical composition of the coating. Here again, extra care should be taken regarding the elemental composition of the deposited materials as it might depend on the position where the elemental analysis is made on the substrate, especially if a spatially resolved analysis is carried out by a technique such as scanning (transmission) electron microscopy combined with energy-dispersive X-ray spectroscopy (S(T)EM-EDS). Typical examples of the formation of compositional gradients on coated surfaces can be found, for example, in [54–55]. Further control on film chemistry is obtained through reactive (magnetron) sputtering. In this case, the metallic target is sputtered by a plasma generated in a mixture of argon with a molecular gas, by using dedicated mass flow controllers (MFC). Oxygen, nitrogen, methane, or hydrogen sulfide can be added to deposit metal oxides, nitrides, carbides, or sulfides, respectively. One example of such tailoring of the film chemistry is reported in [56] where zinc (Zn) and tin/copper (Sn/Cu) targets are both co-sputtered in a reactive H_2_S/Ar reactive plasma for the synthesis of Cu_2_ZnSnS_4_ (CZTS) coatings. In the case of reactive sputtering, MFCs manage the flow (usually expressed in standard cubic centimeters per minute, sccm). This is a key aspect of the reactive process because in most cases, for a critical value of this parameter, the discharge current and voltage, the partial pressure of the reactive gas, and the flux of deposition change significantly, sometimes abruptly. When this critical value is reached, the interaction of the reactive gas molecules and atoms, ionized or not, present in the plasma leads to the formation of a layer of compound material on the surface of the target. For example, a few nanometers thick layer of AlO*_x_* will cover an aluminum (Al) target surface when it is sputtered in an Ar/O_2_ atmosphere. This rather complex phenomenon named target poisoning has been discussed and modeled in, for instance, [57–60]. The value of the critical reactive gas flow depends on parameters such as the volume of the deposition chamber and the system pumping speed, the electrical power applied to the target, the chemical nature of the sputtered material, and the molecular gas.

Before reaching the critical value, the number of reactive species detected in the gas phase is close to zero because the sputtering and subsequent deposition of metal atoms on the chamber walls acts as an auxiliary pumping system for the reactive gas species. This so-called getter effect is efficient for metals having a strong affinity for the reactive gas, as in the case of the Al–O combination. As the target gets poisoned, the partial pressure of reactive gas increases abruptly [61] as evidenced by plasma analysis [62]. Also, the ion-induced secondary electron emission yield (ISEE) of the target in its metallic and poisoned states are different. So-called secondary electrons are electrons emitted by the surface when the latter is bombarded by fast particles such as ions. If more (or less) secondary electrons enter the plasma, the ionization rate of the plasma particles and therefore the ion current at the cathode increases (or decreases). From a practical point of view, if the power supply used to generate the plasma is set to keep the discharge current or the discharge power constant, the voltage will be adapted as the target surface chemistry and the ISEE change. Besides, once the poisoned regime is reached, compound molecules can be sputtered as demonstrated by mass spectrometry analysis of the plasma ion chemistry during the reactive sputtering of Ti in Ar/N_2_ [63] or in Ar/O_2_ plasmas [64]. In these publications, besides Ti^+^, molecules such as TiN^+^ or TiO^+^ and TiO_2_^+^ but also reactive gas molecules, atoms, and ions (N_2_, N_2_^+^, N^+^) were detected in the gas phase. Interestingly, AuO molecules were observed when sputtering Au in an Ar/O_2_ mixture [65], highlighting the rich gas-phase chemistry of sputtering plasmas. Finally, as target poisoning occurs, the deposition flux is found to decrease, sometimes by one order of magnitude or more as reported in [60].

It should be noted that various kinds of electric waveforms can be applied to the cathode to generate the plasma and induce sputtering. Direct current (DC), radio frequency (RF, usually at 13.56 MHz), pulsed-DC, or high-power pulses can be applied. Typically, RF generators are employed when a dielectric target is to be sputtered. Pulsed-DC discharges are generated when a metallic target is sputtered in a reactive atmosphere to synthesize insulating compounds [66]. This situation may lead to unwanted arcing events on the target surface, which are detrimental to the discharge stability and deteriorate the quality of the deposited film because large particles can be emitted from the target surface. Such pulsed-DC discharges, which sometimes also make use of bipolar waveforms, allow for discharging the dielectric layer grown on the target surface during the OFF time and allow for safe operation, see [67] for more information. High-power plasma pulses can be applied as well on the cathode. The goal is to apply a very high peak current pulse without overheating the target material and destroying the underlying magnets. By applying such a very high voltage in a short duration of time, from a few tens of microseconds up to a few milliseconds, at a proper repetition frequency to allow the time-averaged power to be comparable to that applied in other discharge types, one can dramatically increase the plasma electron density. As a result, in such high-power impulse magnetron sputtering (HiPIMS) discharges, the ionization degree of the sputtered metal atoms is significantly increased. Typically, under these conditions, the sputtered species have a higher kinetic energy, and coating properties (on solid substrates) are changed as compared to DCMS discharges. The film crystallinity can be promoted at lower growth temperatures, although sometimes, a too intense ion bombardment may lead to the amorphization of the material [68]. Because they are ionized, the kinetic energy of the film-forming species can also be tuned by applying an electric bias voltage onto the substrate during growth. More recently, bipolar HiPIMS (B-HiPIMS) discharge has been implemented. A positive voltage pulse is applied after the negative pulse generated to sputter and ionize the metal atoms [69]. The kinetic energy of the film-forming species can thus be controlled by the positive pulse voltage to some extent. The kinetic energy of the film-forming species is critical when depositing thin films deposited on a solid substrate. Most likely, it also plays a critical role when a liquid substrate is used. More information on HiPIMS discharges can be found in [70–72].

The abovementioned variations of the plasma characteristics, which, in turn, impact the plasma–surface interaction, can be monitored by ad hoc plasma analysis measurements as described in [73–76].

This section highlights that various process parameters can be varied during a sputtering experiment to manipulate the characteristics of the plasma and tailor the plasma–(liquid) surface interaction to, ultimately, control the characteristics of the NP. Although the external control parameters such as pressure and sputter power may influence simultaneously several plasma characteristics and induce sometimes non-linear phenomena, one can nevertheless highlight some general trends: (i) The nature of the target materials (e.g., the combinations of different targets or the fact that the target is made of an alloy, see Figure 3b), and/or the implementation of reactive sputtering by adding a molecular gas (e.g., N_2_, O_2_ and CH_4_) allow for depositing a very large catalog of materials ranging from pure metals and alloys to metal oxides, nitrides, and other compounds. (ii) The product of pressure times target–substrate distance and the electrical power applied to the cathode controls the deposition flux. The precision achieved in controlling the thickness of films deposited on a solid substrate is typically in the nanometer range. The deposition rate is very stable over time. (iii) The product of pressure times target–substrate distance, the sputter power, the type of power supply utilized (DC vs HiPIMS plasmas), and the pressure and the nature of the gases utilized to generate the plasma control the energy deposition and, more specifically, the fluxes of particles reaching the liquid surface. Amongst the species that may play a role in NP formation are (1) atoms/molecules such as Ar neutrals, Ar metastables (Ar atoms have metastable states lying at 11.6 eV), sputtered metal atoms, and molecules such as N_2_ or O_2_ if reactive sputtering is implemented; (2) ions such as Ar^+^, metal ions (M^+^) but also sometimes multiply charged ions (Ar^2+^, M^2+^). If the plasma is generated in an argon/reactive gas mixture R^+^, R_2_^+^, R^2+^, and MR*_x_*^+^ ions (with R being, e.g., O or N) can also be found; (3) chemically reactive radicals such as O or N atoms produced through dissociation reactions during reactive sputtering. O atoms can also originate from dissociated H_2_O or O_2_ molecules present in the chamber if the quality of the vacuum is not good; (4) electrons and photons, with energies spanning from the hard UV up to the IR domain, may eventually promote bond breaking of the liquid host molecules and heating.

Researchers working with the sputter deposition process should be aware of these “control knobs” to ultimately control the NP formation during the SoL approach.

#### 1.3 Formation of NPs in solutions

Colloidal synthesis, which is also called the “classical wet chemical approach”, is one of the well-studied methods to produce NPs with desired size, shape, and composition [2]. Colloidal synthesis allows one to obtain metal, oxide, halide, chalcogenide, and other types of NPs but the mechanisms of their formation are different, even though all of them include an initial nucleation step followed by a growth step [77]. In this chapter, we will concentrate on the formation of metal NPs in solutions because, up to June 2021, the vast majority of NPs produced by the SoL technique has been metallic [13–14,78].

As it was described in section 1.1, four components are normally needed for the colloidal synthesis of metal NPs: (i) the metal precursor*,* usually a salt such as HAuCl_4_, is chemically reduced by a (ii) reagent (e.g., sodium citrate, sodium borohydrate, ascorbic acid, glucose, hydrazine, or various amines) in the presence of a (iii) stabilizing agent (e.g., organic thiols, amines, acids, or various surfactants, i.e., compounds having active groups with S, N, or O atoms) in the chosen (iv) solvent [2,79]. The number of reagents might be less than four when the reduction process is initiated by light, when the reducer molecules protect the obtained NPs, or when the solvent plays either the role of the reducer or the stabilizer [79]. The final amount of NPs in the solution and their final size depend on the kinetics of NP formation and, mainly, on the ratio between the rates of nucleation and growth steps [77]. The stability of obtained NPs depends on many factors but the affinity of the stabilizer reagent to the NP surface plays a leading role [79].

Two main models have been actively used to describe the formation of primary metal NPs, namely the La Mer model and the autocatalytic model [80]. Even though both models were proposed decades ago, the colloids community is still arguing which one is better [81–85]. La Mer and Dinegar proposed a theory describing the formation of monodispersed sulfur hydrosols in 1950 [86]. Two years later, La Mer published a critical work on nucleation theory applied mostly to the formation of sulfur and barium sulfate sols [87]. It is important to highlight here that the La Mer model was not built based on experimental observations about the formation of metal NPs. Basically, La Mer and co-workers applied classical nucleation theory (CNT; this abbreviation was widely used by colloidal chemists years before carbon nanotubes became a hot research topic) that was developed by Döring and Becker in 1935 [88]. It is worth noting that CNT treats nuclei as fragments of the bulk phase having the same macroscopic properties. This, as we know today, is not accurate since NPs and especially their nuclei have a different surface free energy [77]. The mechanism of sulfur (S) sol formation proposed by La Mer includes the formation of monomers, S_2_ (Equation 1), their burst homogeneous nucleation (Equation 2), and the subsequent diffusive, agglomerative growth (Equation 3):


[1]

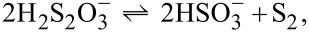




[2]

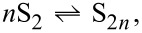




[3]

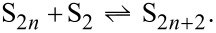



**Figure 6:**
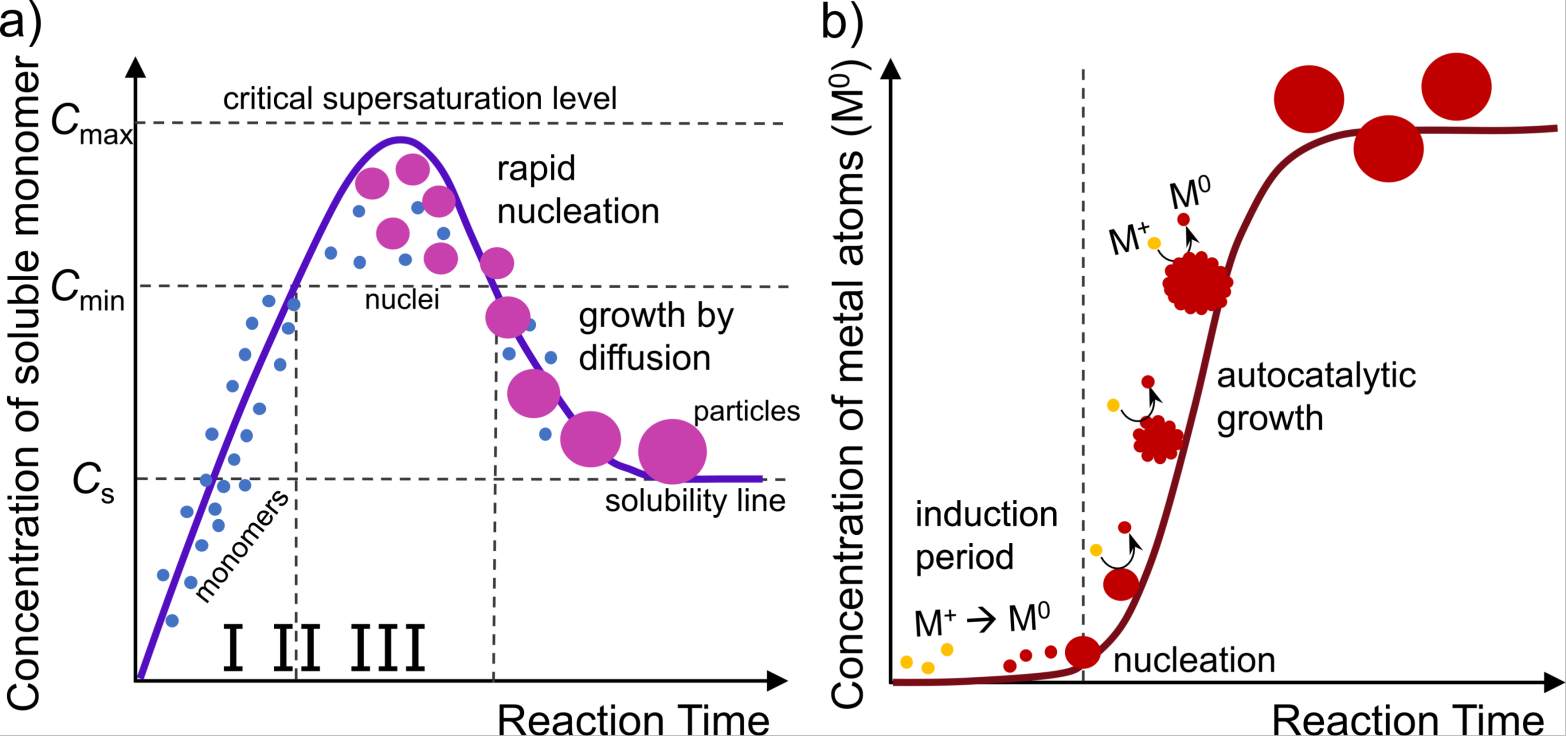
Schematic diagrams representing the NP formation as a function of time: (a) La Mer model and (b) autocatalytic model.

As one can see in Figure 6a, the La Mer mechanism can be described by a succession of three phases. Phase I is a rapid increase of monomer (S_2_) concentration in the reaction solution. Phase II is an extremely fast nucleation process via a stepwise sequence of S_2_ monomer additions until the formation of nuclei having a critical size is reached. Since La Mer worked in a frame of statistical mechanics, the nucleation step can occur only in supersaturated solutions, when the probability of encounters between monomers is high. After the formation of the first nucleus, the monomer concentration falls below supersaturation and new nuclei cannot be formed. Phase III is the further growth of the particles limited by the diffusion of the monomers to the nuclei surface. The assumption that burst nucleation significantly reduces the concentration of monomers allows for achieving the key separation of the nucleation and growth steps, which is a mandatory condition for the formation of monodisperse particles (but it can be achieved in the frame of other models). The readers interested in CNT and mathematical equations behind the La Mer model [86] are referred to the reviews in [80,83,85].

For almost 50 years, La Mer's pioneering mechanism was the only available mechanism that explained the formation of monodisperse particles and provided a mathematical model for fitting the kinetic curves [89]. As a result, it was applied to any colloidal system, including colloidal dispersions of metal NPs, and finally was considered as “overcited” [90]. In reality, this mechanism describes very well the formation of sulfur sols and analogous systems, for example, AgCl, BaSO_4_, and TiO_2_ NPs [85,91].

The main problem of generalizing the La Mer mechanism to other systems is that homogeneous nucleation does not always take place in a supersaturated solution [89]. For example, transition-metal NPs can nucleate and grow from diluted solutions [77] as shown for the first time by Turkevich in 1951 [92]. Turkevich and co-workers studied the reduction of HAuCl_4_ by citrate anions (which also plays the role of a stabilizer for the Au NPs) at different temperatures and different reagent concentrations [92]. By means of transmission electron microscopy (TEM), it was shown that the Au sol formation process consists of an induction period followed by a rapid increase in the number of particles, followed by a linear increase, followed by a rapid decrease in the growth rate. In other words, the typical kinetic curve has a sigmoidal S-shape (Figure 6b). Turkevich and co-workers proposed an “organizer” nucleation theory, according to which the Au ions are bound together by an “organizer” (citrate ion) into “copolymers” [92]. Unfortunately, the authors did not formulate a clear detailed mechanism of the process describing the underlying, kinetically dominant, elementary steps. So, their “organizer” theory looked very complicated as compared to the simpler La Mer mechanism. As a result, the work [92] had been cited scarcely until the production of Au NPs became a hot research topic. The reduction of Au ions by sodium citrate was also studied by Takiyama in 1958 [93]. He noticed that (i) the process starts with an induction period followed by rapid nucleation, (ii) the number of particles does not change after the nucleation, and (iii) particles grow by an autocatalytic reaction on the nuclei surface, that is, the surface atoms of the metal NPs reduce the metal ions present in the solution and, thus, NPs grow further. Takiyama used empirically chosen mathematical equations to fit the obtained kinetic curves and calculated rate constants under different conditions. However, he did not formulate a step-by-step mechanism and his work stayed almost unnoticed. There had been no sufficient progress regarding the kinetics of NP formation during the 35 years after Takiyama’s work. Studies of pulse radiolysis reduction of silver (Ag) ions showed the presence of small ionic Ag*_р_^z^*^+^ clusters (*p* = 2–9, *z* = 1–2) that grow and become nuclei of the final NPs [94–95]. Because silver clusters absorb light at different wavelengths than the surface plasmon resonance (SPR) band of Ag NPs (which is used for monitoring of NP growth kinetics by UV–vis spectroscopy) one can spot the induction period on the sigmoidal kinetic curves. Noteworthy is the important contribution by Huang et al. [96], who proved the autocatalytic nature of NP growth by an easy experimental test. They introduced Ag NPs to a solution containing Ag ions and a reducer. This led to a significant increase in the process rate due to the reduction of Ag ions on the NP surface.

In 1997, Watzky and Finke published a fundamental work, which included a simple mechanism describing the formation of metal NPs in diluted solutions, a kinetic model, and a set of mathematical equations that allowed one to process the kinetic data [89]. They suggested a two-step mechanism, which consists of a slow, continuous nucleation (Equation 4) and a fast autocatalytic surface growth (Equation 5):


[4] $$n{\text{M}}^{z+}+nz{\text{e}}^{-}\to {\left({\text{M}}^{0}\right)}_{n},$$

[5] $${\left({\text{M}}^{0}\right)}_{n}+{\text{M}}^{z+}+z{\text{e}}^{-}\to {\left({\text{M}}^{0}\right)}_{n+1}.$$

**Figure 7:**
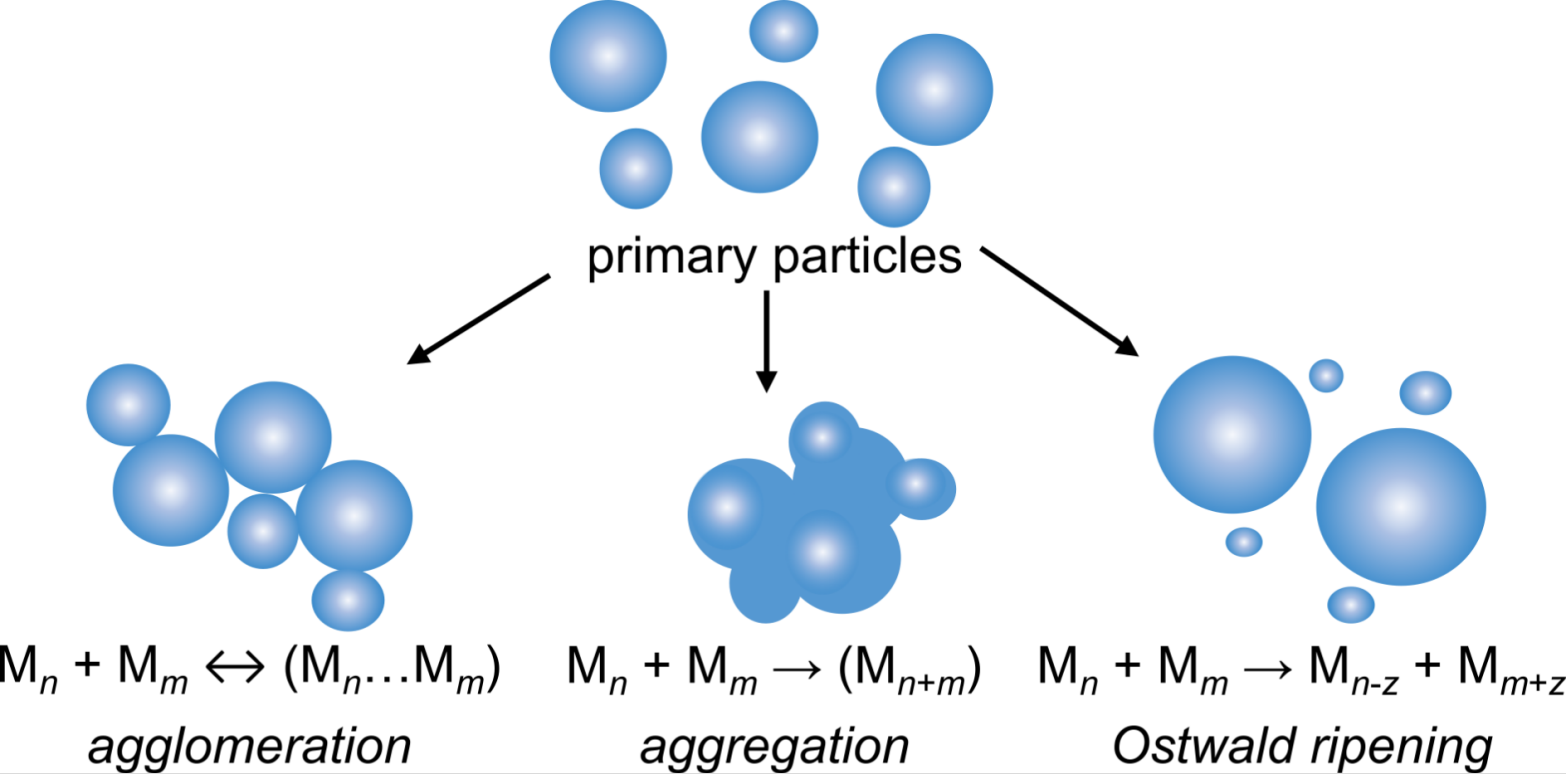
Schematic representation of the secondary growth processes.

According to the Finke–Watzky model, the process of NP formation is limited by the rate of metal precursor reduction. Nucleation (Equation 4) is limited by the homogeneous reduction of ionic metal species (M*^z^*^+^) to metal atoms (M^0^), which aggregate until the critical nucleus size ((M^0^)*_n_*) is reached. The future growth of the formed nuclei is limited by the autocatalytic reduction of metallic species on their surface (Equation 5). NPs formed via these steps are called primary particles according to the colloid chemistry classification [97]. Controlling the ratio between the rates of the processes in Equation 4 and Equation 5 allows one to control the number and size of the primary particles. Fast nucleation leads to the formation of large amounts of nuclei and smaller final primary particles, while a slow nucleation process yields a reduced amount of seeds, which finally leads to bigger particles. Separation of the nucleation and surface growth processes is the main condition for the production of monodisperse primary NPs [77]. The Finke–Watzky autocatalytic model was first proposed for small iridium (Ir) NPs [89]. Yet, it has been shown later by Finke’s group and other research teams that it might be successfully adapted to quantitatively study the formation of Au [98–101], Ag [102–104], and other transition metal NPs [77,105–106]. The interested reader can find more detailed information about the Finke–Watzky mechanism, the mathematical analysis of data, and links to useful references in [77,107–109]. It is important to stress that the autocatalytic model does not deny the possibility of the further growth of the metal NPs by secondary processes such as reversible agglomeration, irreversible aggregation, or Ostwald ripening (Figure 7) [77,79–80,83,110]. When agglomerates or aggregates form a precipitate in colloidal solution, the process is called coagulation. The fact that such secondary processes take place in a colloidal solution of primary metal NPs means that these primary particles were not stabilized well enough [79,83].

Despite the fact that the colloidal synthesis of NPs has been actively studied for decades because of the growing interest in nanomaterials, the majority of synthetic protocols were found and optimized by empirical procedures and often have reproducibility issues [110]. The main reason for this is the lack of fundamental kinetic studies without which it is impossible to fully understand the mechanisms of NP formation and to control NP synthetic procedures on the same level as in modern organic chemistry [110]. Nevertheless, the small number of quantitative kinetic studies of NP formation and accurate analysis of the obtained products allowed researchers to distinguish factors that significantly affect the reproducibility of the colloidal synthesis and properties of the obtained NPs. The most important parameters are:

1. **Reagent concentration**. Concentrations and volumes of reagents must be fixed because their variations lead to different NP sizes (it is not that easy to upscale even for the well-known synthetic protocols). Ideally, the amount of stabilizer molecules should be of the same order of magnitude as the number of surface atoms of NPs. A lack or an excess of stabilizer might lead to NP aggregation [79].
2. **Speed of introduction of the reagent into the reaction system.** If a solution of metal precursor or reducer is to be injected into the reaction vessel after a certain delay, it should always be done in the same way, ideally by a machine, to exclude time variations that will affect the local concentration (supersaturation) and therefore the rates of nucleation and growth processes.
3. **Reaction temperature.** Temperature affects the rates of nucleation and growth processes (which follow the Arrhenius law) [77,111] and many other parameters such as solvent viscosity, species diffusion coefficients, and adsorption of protective ligands onto NP surface [79]. So, variations of the “room temperature” in different laboratories might explain the different sizes and stability of obtained NPs.
4. **Light.** The level of light should be similar during all experiments and metal precursors should be stored in dark places because UV light might cause the formation of nuclei that will affect the size of final NPs and the reproducibility of the synthesis.
5. **Impurities.** Even small amounts of chemical impurities affect the reproducibility of the colloidal synthesis, especially during the synthesis of NPs with non-spherical morphology [4].
6. **Dust.** Dust particles present in the solvent affect the size distribution of formed NPs [77]. Filtration of reagents allows for obtaining NPs with narrow size distribution [112].

Because SoL is a combination of the PVD process and colloidal synthesis, it is important to consider the factors mentioned above to be able to create a well-reproducible method of NP synthesis.

### 2 Sputter deposition onto liquids

#### 2.1 Experimental parameters affecting the formation of nanoparticles

Composition, size, shape, size distribution and stability of NPs produced via SoL depend on many experimental parameters that can be divided into two groups, namely sputtering parameters and host liquid parameters. Sputtering parameters include the sputter power, current, voltage, time, the distance between target and substrate surface, the working gas pressure and gas mixture, and the type of sputter plasma. All these parameters are well-studied in the case of (magnetron) sputtering on solid substrates as it was discussed earlier in section 1.2. It is well-known that the sputtering parameters control the flux of sputtered material, namely the number of species that will reach the substrate surface per time unit and unit area, but also the kinetic energy of the sputtered species, as well as other parameters (e.g., flux and wavelength of the light emitted by the plasma). Because all research groups carry out experiments in different vacuum chambers, it is extremely important to know the flux used to be able to compare the obtained results and to define normalized parameters to describe the interaction of the plasma with the surfaces. At the same time, it is necessary to know the parameters of the host liquid, not only the liquid composition and its physical properties but also its volume and the thickness of the liquid layer, to be able to estimate the concentration of sputter material in the resulting NP solution and to consider if the liquid surface is heated by the plasma [42,47].

Despite the fact that most researchers accept that experimental parameters influence the processes of nucleation and growth of NPs, it is usually almost impossible to compare results obtained in different labs because the applied conditions are too different and because not all experimental parameters are always provided by authors. Important information such as the value of discharge current and voltage, and the deposition rate are frequently lacking in the experimental sections. Nevertheless, after summarizing all experimental conditions provided in more than 112 research papers published since 1996 (see Supporting Information File 1) we found several common trends that we discuss below.

**2.1.1 Sputtering parameters:** Analysis of the experimental parameters provided in papers related to SoL has shown that a given research group uses similar conditions from paper to paper (sputtering device, working distance, working gas composition, and its pressure). Quite often, the only parameters changed are target material and/or host liquid. Unfortunately, such cases do not fully suit for direct comparison of the results, because changing the target means also changing the flux and kinetic energy of sputtered species even if the applied power and working pressure were similar. The sputtering yield is a direct function of the nature of the sputtered material, as shown in Figure 4. The transport of the atoms from the sputtering target surface towards the liquid surface is also a key issue and depends on the nature of the atoms moving through the gas phase, their initial kinetic energy, and the angle of ejection from the target surface. At the same time, each research group checked at least once the influence of sputtering parameters such as current and/or voltage and sputter time. We summarize the trends reported in the literature below.

**2.1.1.1 Power, current, voltage (i.e., particle flux):** The effect of the discharge current and/or discharge voltage on the NPs was studied in [113–129]. Controversial data were obtained by different research groups. It is worth noting that one important issue to enable the comparison of data obtained in different studies is to use power and current densities, that is, the values are divided by the area of the target, instead of the current and power alone. But the best is to provide the reader with the intrinsic properties of the plasma such as the flux of particles, which is a parameter underlying the current/power parameters.

The authors of the following papers found out that the size of NPs increases with the current [113,117,121–123,126–127,129]. For example, according to Suzuki’s research [113], an increase in discharge current from 10 to 40 mA during the sputtering of Ag onto 1-butyl-1-methylimidazolium hexafluorophosphate (BMIM-PF_6_) leads not only to a higher total Ag concentration in the solution and a subsequent increase of absorbance of the solution at the SPR peak maximum but also to an increase in the size of Ag NPs from 5.7 to 11 nm. In the case of Au sputtering onto 1-(2-hydroxyethyl)-3-methylimidazolium tetrafluoroborate (HyEMI-BF_4_), changing the current from 10 to 40 mA resulted in changing the size of Au NPs from 2.7 to 4.7 nm [126]. Studying MS of Cu onto pentaerythritol ethoxylate (PEEL) showed that the NP size depends on the current. At low current values (10–20 mA) the average size of NPs was between 2 and 3 nm but at a higher current value (100 mA) the size distribution of obtained particles become wider and divided in two populations with sizes around 5.5 and 8 nm [117]. In another study where platinum/copper (Pt/Cu) NPs were obtained by using an alloy target for the sputtering onto polyethylene glycol (PEG), it was reported that increasing the current from 10 to 50 mA leads to the formation of bigger NPs, from 1.3 to 4.5 nm, respectively [127]. The authors explained it by the fact that usage of higher current allows for sputtering a higher number of atoms per time unit. Thus, the atoms will collide with a higher frequency inside the liquid, hence forming larger NPs. Wender et al. reported that the size of NPs depends on the current and the discharge voltage. According to them, the NP size increases with increasing the current when sputtering Au onto 1-butyl-3-methylimidazolium bis(trifluoromethylsulfonyl)imide (BMIM-TFSI) [129] and the population of small Au NPs increases with increasing discharge voltage in case of 1-butyronitril-methylimidazolium bis(trifluoromethylsulfonyl)imide [(BCN)MIM-TFSI] [128]. At the same time it was reported that an increase in discharge voltage leads to an increase in diameter of Ag and Au NPs produced by sputtering onto castor oil [124–125]. An augmentation of the voltage applied at the cathode, at a given working pressure, will lead to an increase of the current, and therefore of the flux of atoms reaching the liquid surface.

According to Hatakeyama et al. [114], an increase in the discharge current (from 20 to 40 mA by varying the voltage) during the sputtering of gold onto 1-butyl-3-methylimidazolium tetrafluoroborate (BMIM-BF_4_) mainly increases the total concentration of metal and the number of Au NPs in the solution but hardly affects the NP size and their size distribution. According to Qadir et al. [116], variations of the current do not affect the formation of palladium (Pd) NPs in 1-thioethyl-methylimidazolium bis(trifluoromethanesulfonyl)imide [(HSE)MIM-TFSI]. Yonezawa’s group [120] showed that only the absorbance of the Au NP solutions increases proportionally with the current (from 10 to 30 mA) but not the size of the NPs after sputtering onto PEG. It was also shown that the size of metal NPs does not depend on the sputter power in the case of sputtering iron (Fe) onto the surface of silicon oil [130] and in the case of sputtering of Ag or Au onto castor oil [131–132].

We agree with Hatakeyama et al. [114] that researchers who discuss about the influence of current and voltage on the NP size never considered the fact that increasing the discharge current causes a heating of the host liquid surface and eventually also a temperature increase of the target. As it can be evidenced in the next subsections an increase in liquid temperature changes its viscosity and might lead to the formation of larger NPs.

**2.1.1.2 Sputter time:** Influence of sputter time on the NP formation was studied in [12,113,117–118,120,122,125–126,129–149]. According to most of the researchers, the time of sputtering does not affect the size of NPs. However, the number of formed NPs linearly increased with increasing the sputtering time. In this case, adjusting the sputtering time allows for getting the desired number of NPs in the host liquid [12]. For example, long term (24 h) deposition of Cu onto 1-butyl-3-methylimidazolium bis(trifluoromethylsulfonyl)imide (BMIM-TFSI) allowed the authors to produce 1 g of Cu NPs in 50 mL of IL [150].

At the same time, we cannot ignore several papers where authors mentioned the increase of the mean NP diameter [126–127,138–139]. In the work by Sugioka et al. [126], Au was sputtered on a very thin layer of HyEMI-BF_4_ and a quite significant change of NP size was detected when increasing the deposition time: from 2.7 to 5.1 nm after 0.5 and 5 min, respectively. In [127,138–139], PEG was chosen as a host liquid. A slight increase in the mean particle diameter was reported for Au sputtering (from 5.6 ± 1.8 nm after 5 min to 5.9 ± 0.8 nm after 15 min) [138–139], and for Pt/Cu prepared via sputtering of an alloy target (from 1.2 ± 0.4 nm after 15 min to 3.1 ± 1.7 nm after 4 h) [127]. It seems that the authors of these papers [126–127,138–139] did not consider the fact that irradiation of the host liquid by the plasma species causes heating. For example, some researchers used a very thin layer of host liquid (0.08 mL of IL was spread onto a 4 cm^2^ glass plate with 3.5 cm of working distance [151] or 0.6 mL of IL was spread onto a 10 cm^2^ glass plate [113,152–153]), which makes overheating very likely. This leads to a change in the viscosity and surface tension and might affect the diffusion of the sputtered species in the liquid matrix which, in turn, changes the size of final NPs.

**2.1.1.3 The working distance between target and liquid:** According to Hatakeyama et al. [114], the working distance between the target and the host liquid surface does not affect the size of NPs. The variation of this distance from 25 mm to 50 mm and finally to 75 mm, with all other parameters fixed, showed that the number of NPs increased with decreasing of the distance, but the size of Au NPs and its distribution remained the same.

As it was found during vacuum evaporation of silver onto running silicon oil, changing the deposition rate by changing the distance between metal source and liquid might lead to heating of the liquid surface for the shortest distances, subsequently changing the viscosity for long deposition processes and impacting the size of the final NPs [154].

**2.1.1.4 The working gas composition:** Ar was used as a working gas in most of the papers with the exception of several works [12,120,142–143,147,155–157] that will be considered below. The main reason for the wide usage of Ar for SoL, and sputter deposition in general, is that it is a cheap and inert gas that will not react with the liquid substrate. The sputtering yield is also high because Ar is a rather heavy atom. Moreover, parameters of Ar-based plasmas are already well studied in the case of sputtering onto solid substrates.

Air was used as a working gas for sputtering of Au onto various ILs (1-ethyl-3-methylimidazolium tetrafluoroborate (EMIM-BF_4_), *N*-trimethyl-*N*-propylammonium bis(trifluoromethylsulfonyl)imide (TMPA-TFSI) [12], and BMIM-PF_6_ [157]), (6-mercaptohexyl)trimethylammonium bromide (6-MTAB) [142], PEG [120], PEEL, and pentaerythritol tetrakis(3-mercaptopropionate) (PEMP) [143]. Oxygen was used to sputter Au onto liquid crystals of 4-cyano-4′-pentylbiphenyl [147]. Since Au is a noble metal, no oxidation processes were detected in the samples, although AuO molecules have been detected in the gas phase when sputtering gold in an Ar/O_2_ atmosphere in earlier studies [65]. Comparing the sizes of Au NPs obtained using air 1.9 ± 0.46 nm [12] and Ar 2.2 ± 0.4 nm [126] or 2.3 ± 0.3 nm [153] during sputtering onto TMPA-TFSI shows that, in the case of Au, changing the working gas does not affect the size of the product. However, the exchange of the target material from Au to a less noble metal, namely Cu changes the situation. As it was shown by Yonezawa’s group, sputtering Cu onto PEMP in air atmosphere, that is, reactive sputtering, leads to the formation of Cu_2_O NPs [155] that further react with SH groups from PEMP to form Cu_2_S NPs.

It was reported that the working gas composition influences the size of Pt NPs [156]. The mean diameter increased from 2.24 ± 0.358 nm in Ar to 3.28 ± 0.60 nm in N_2_. The authors proposed that the ions of the N_2_-based plasma eject larger Pt clusters than those of the Ar-based plasma, but no additional research was done in that case. Here, some comments can be made. First, the deposition rate decreases with increasing the nitrogen partial pressure as reported, for example, in the case of Ti, Cr, In, Sn, Al, and Si targets sputtered in Ar/N_2_ atmosphere [158]. The difference in deposition flux during SoL would lead to a reduced heating of the host liquid, which might affect the diffusion of sputtered atoms and the size of the final NPs. Still, in line with the use of a molecular gas, it might also be speculated that N atoms generated in the plasma interact with the liquid surface and modify its chemistry to some extent. Second, the emission of metal atom clusters in conventional sputtering processes, in which the bombarding ions have sub-kiloelectronvolt kinetic energy, is a rather marginal phenomenon. Simulations works (see, e.g., Figure 2 in [159]) reveal that the yield of cluster emission (*Y*) decreases exponentially with the cluster size and the kinetic energy of the impinging Ar^+^ ions. According to the above-cited article, the yield of emission of a five-atom silver cluster is ca. 5000 times lower than the sputtering yield of single silver atoms when 500 eV Ar^+^ ions hit the surface. These authors provide a mathematical expression for the yield *Y*(*n*) of nascent, that is, right after the cluster has been emitted, *n*-atom clusters, which is *Y*(*n*) ∝ *n*^−α^. The exponential factor α equals 7–9 for Ar^+^ ions with 250 eV energy, 5.5–6 for 500 eV Ar^+^, and 3–4 for 5 keV Ar^+^. Furthermore, it should be highlighted that polyatomic clusters may spontaneously break up [160]. Consequently, the final size of the cluster population is smaller than the one predicted by the expression of *Y*(*n*).

**Figure 8:**
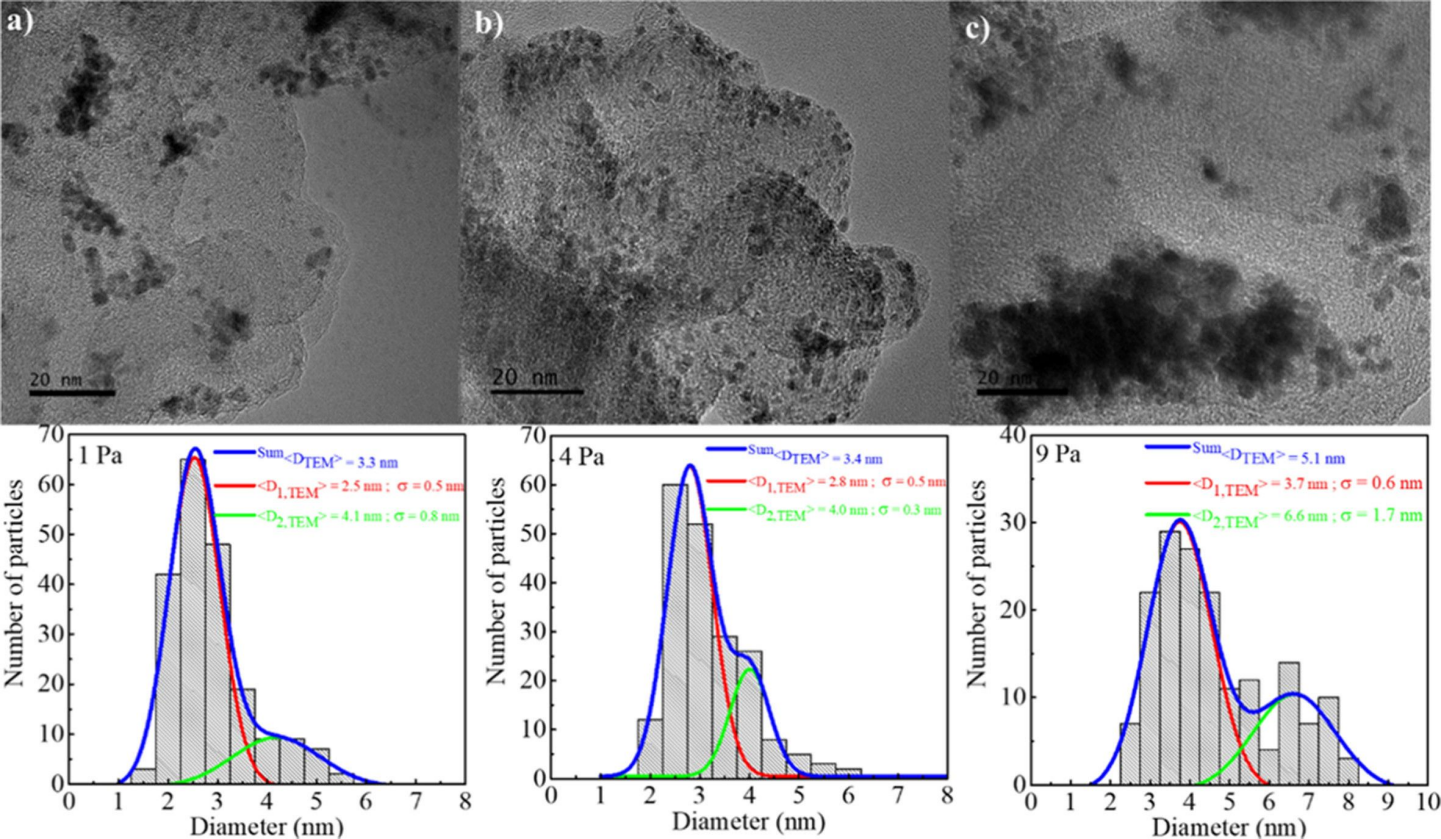
TEM images and corresponding size distributions of Pt NPs produced via MS of a platinum target onto glycerol at pressures of 1, 4, and 9 Pa, respectively. Figure 8 was reprinted with permission from [163]. Copyright 2021 American Chemical Society. This content is not subject to CC BY 4.0.

**2.1.1.5 Gas pressure:** The effect of changing the working pressure, while keeping the other parameters identical, was studied in [11,131–132,161–163]. Increasing the pressure from 0.067 Pa to 2 Pa during the sputtering of Ag onto castor oil led to a slight increase in the diameter of primary Ag NPs from 2.1 nm to 2.8 nm [131]. Sputtering of Pt onto glycerol at a pressure of 1, 4, and 9 Pa led to the formation of spherical Pt NPs with the mean diameter of 2.5 nm, 2.8 nm, and 3.5 nm, respectively [163] (Figure 8).

Accordingt to Wagener et al., increasing the pressure from 1 to 30 Pa leads to increasing the NP size from 5 to 20 nm in case of sputtering Ag and Fe onto silicon oils [11,161–162]. We should mention that 30 Pa is an unusually high pressure for sputtering as the deposition rate is, in this case, extremely low and aggregation in flight most likely happens like in dedicated gas aggregation source (GAS) experiments. Choukourov et al. recently reported the combination of a GAS (at 75 Pa pressure) with a liquid substrate for the production of Ag and Cu NPs [164–165]. Molecular dynamics-based simulation data are also available on this topic [166].

Despite the fact that pressure can affect the size of NPs is known since 1996, the authors of some important works [114,120] did not control the pressure and let it range between 2 and 30 Pa. Unfortunately, we cannot compare the results obtained for the same target material and host liquids at different working pressures because all other experimental parameters affecting the flux were very different.

**2.1.1.6 Type of sputtering plasma:** As it was discussed earlier RF, DC, or HiPIMS power supplies can be used for plasma generation. The choice of the power supply depends on the target material and the desired product properties. In the case of SoL, most of the works were carried out in DC mode. RF power supplies were used for the production of Au [167], Pt [168], and Pt/Ni [169] NPs and Ag films [170–172].

Unfortunately, different experimental conditions do not allow for a direct comparison of the results provided for Au NPs from [167] (RF; 0.53 Pa (Ar); 30 W; 9 min; 2-inch target, 150 mm distance) and [126] (DC; 20 Pa (Ar), 10 mA; 0.5 min; 2-inch target, 35 mm distance). Au NPs obtained via sputtering onto the IL 1-ethyl-3-methylimidazolium ethyl sulfate (EMIM-EtSO_4_) were smaller when a RF power supply was used (1.3 ± 0.7 nm for RF vs 2.5 ± 0.6 nm for DC). Yet, it is impossible to draw any conclusions from this study, especially considering the secondary growth processes taking place in the Au NP–IL mixtures.

DC and HiPIMS power supplies were used for the production of Cr–Mn–Fe–Co–Ni alloy NPs in BMIM-TFSI [173]. It should be noted here that, in general, sputtering of monomaterial or alloy targets made of noble metals leads to the formation of spherical monocrystalline NPs. However, the use of a complex target in [173] led to different results. On one hand, for DC, the initial NPs were amorphous with a mean diameter of 1.7 ± 0.2 nm, the size increased during the electron-beam assisted crystallization process up to 2.6 ± 0.3 nm. On the other hand, the HiPIMS as-deposited NPs were already crystalline and had a bigger diameter of 3.2 ± 0.5 nm. The authors explained the difference by the fact that species sputtered with HiPIMS are ionized and have a higher kinetic energy than neutral species sputtered with DC discharges. This might affect nucleation and growth processes and allow for the formation of crystalline phases at lower temperatures. Depositions of Ag and Au onto castor oil with DC and HiPIMS power supplies have shown that the particle size is also affected by the plasma type. NPs obtained at the same working pressure in castor oil in HiPIMS mode were more stable and twice as large as those obtained in DC-MS mode: 2–3 nm for DC-MS vs 5–7 nm for HiPIMS with a Ag target [131] and 2.4 ± 0.9 nm for DC-MS vs 5.2 ± 0.8 nm for HiPIMS with a Au target [132]. The positive voltage applied to the cathode, in the bipolar HiPIMS mode, allowed for an additional increase of the size of primary Ag NPs and their aggregates. This is presumably due to an increase of the temperature of the top layer of the host liquid. An increase in temperature leads to a decrease in viscosity and hence to a higher number of collisions yielding larger NPs. Thus, depositions with a HiPIMS power supply [131–132] might be used as an alternative to the annealing process [174–176] for producing larger metallic NPs.

**2.1.1.7 Target temperature:** The temperature of the target is increasing during the sputtering process. Hence, a cooling system is a necessary part of the vacuum chamber since it allows for a stable deposition rate. The influence of the target temperature was studied by Hatakeyama et al. [114]; it was shown that an increase in target temperature leads to an increase in the NP size. The authors explained it by “the change in kinetic energy of the sputtered Au particles and their degree of clustering“ [114]. It can be speculated here that an increase in target temperature leads to an increase in sputtering yield (as reported in, e.g., [177]) which causes heating of the host liquid and subsequent formation of larger NPs in the limited volume of the solution. A heated target may also emit IR radiation [178], which subsequently also heats the liquid.

**Figure 9:**
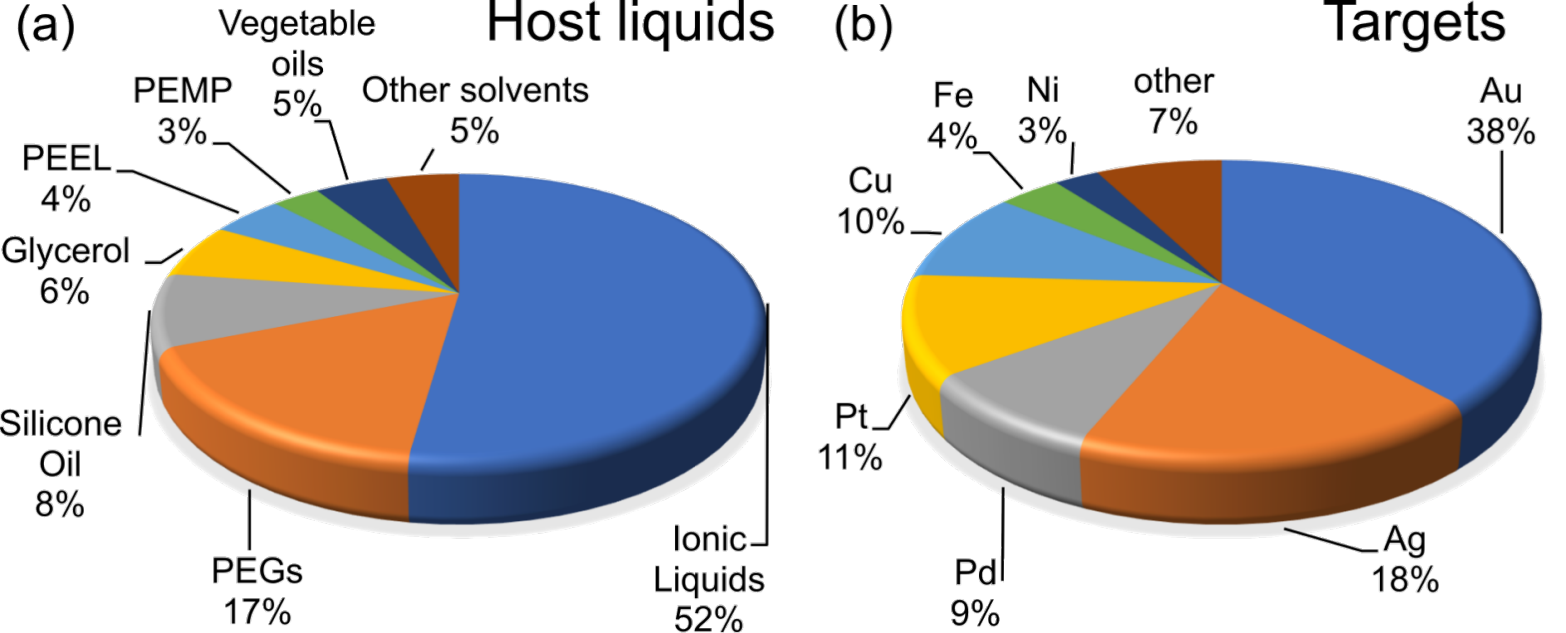
Statistics data of the most commonly used (a) host liquids and (b) target materials for the SoL approach as of September 2021.

**2.1.2 Host liquid parameters:** The main common characteristic of all host liquids used for SoL processes is their low vapor pressure, which allows for a use inside a vacuum chamber. As one can see in Figure 9, the most widely used host liquids are ILs, PEGs, and silicon oils. Of course, not all compounds mentioned in Figure 9 are perfect stabilizing agents for NPs, especially in terms of the “wet synthesis approach”. The stability of the obtained solutions depends on the affinity of the deposited target material to a given host liquid but, generally, NPs can be dispersed in these viscous liquids for a long time before coalescence and aggregation processes take place [14]. Here, we review how the host liquid parameters affect the properties of NPs obtained via sputtering.

**2.1.2.1 Nature of host liquid:** The nature of the host liquid, namely the chemical structure of the solvent, is one of the most important parameters for the NP formation process. In a typical SoL experiment, there are only two components in the reaction mixture, that is, sputtered species of the target material and the host liquid. The latter plays the role of dispersion medium and capping agent at the same time. Thus, the presence of functional groups having a high affinity to sputtered species or the ability to stabilize NPs via electrostatic repulsion and steric stabilization must be considered during the selection of a suitable host liquid. Here, theoretical chemistry studies such as quantum chemistry-based calculations and molecular dynamics simulations may provide useful insights, as shown in [131–132,163,174–175,179]. Below, we highlight some trends.

**2.1.2.1.1 Different host liquids/fixed target material and sputtering parameters:** There are several works in which the same metal was sputtered onto different ILs, namely Au [115,126,153,180–183], Ag [184–185], Pt [186], Pd [116], and In [187]. Most researchers share the point of view that an increase of IL viscosity leads to slower diffusion of sputtered species and larger sizes of NPs. Of course, the presence of hydroxy functional groups in IL complex cations provides stronger adsorption on the NP surface and leads to the formation of smaller particles [126]. At the same time, according to Dupont et al., the size of NPs depends more on the structural organization (anisotropy) of the IL than on the nature of the functional groups present in the imidazolium cation [116]. It was independently shown by Torimoto’s [126] and Ludwig’s [188] groups that sputtering onto a mixture of ILs allows one to control the size of NPs by controlling the composition of the liquid substrate.

The presence of functional groups having a high affinity to the sputtered metal allows one to get smaller NPs while the other parameters are fixed, as it was shown for Au in the case of sputtering onto thiolated (PEG-SH, PEG-S_2_H_2_) and aminated (PEG-NH_2_) polyethylene glycols [138–139]. Changing the composition of a host liquid mixture of oleic acid and oleylamine allows one to control the NP size and colloidal and oxidation stability of Au and Cu NPs [189]. A discussion on the NP stabilization by host liquids can be found in section 2.2.

**2.1.2.1.2 Fixed liquid and almost similar sputtering parameters (except the flux)/different target materials:** Comparing the NP sizes obtained under similar experimental conditions has shown that the mean diameter of metal NPs in the solution increases as Pt ≤ Pd ≤ Au ≪ Ag in the host liquids TMPA-TFSI, BMIM-PF_6_, BMIM-TFSI, PEG-600, and glycerol (see Supporting Information File 2). The size of bimetallic Au*_x_*Pd_1−_*_x_* and Au*_x_*Pt_1−_*_x_* NPs made by co-sputtering always increases with increasing *x* [136,140,152,190–193]. In the case of co-sputtered Au*_x_*Ag_1−_*_x_* NPs, the mean diameter of alloy NPs increases with an increasing fraction of Ag [194].

It should be noted that, in the case of noble metals, secondary growth processes were mentioned for dispersions of Au [115,118,138,140,143–144,167,183,189,195], Ag [131,164,185,196], and Pt [185] NPs. When metals such as Ti [179,197], Cu [117,155,174–175,189,198–199], Fe/Al [200], Mo [201], or In [187] were sputtered , the products were oxidized due to reaction with the host liquid and/or air after removing the samples from the vacuum chamber.

**2.1.2.2 Additional ligands in the host liquid:** It was shown that the introduction of an additional stabilizing agent containing thiolate, amine, or carboxy groups promotes the formation of NPs of smaller size and with a more narrow size distribution than in the case of a pure host liquid [119–120,161–162,202–210]. For example, Yonezawa’s group demonstrated that in the case of sputtering of Ag onto pure PEG-600 (2-inch target; 50 mm distance; 2 Pa (Ar); 20 min; 10 g of PEG) the size of Ag NPs is 7.4 ± 3.6 nm. After adding a few percents of capping agent, the mean diameter decreases to 2.2 ± 0.5 nm for 11-mercaptoundecanoic acid (MUA) [205] and to 2.7 ± 0.5 nm for sodium 3-mercaptopropionate (SMP) [206]. This approach allows for synthesizing ultrasmall highly fluorescent clusters and for controlling the size of the NPs without changing sputtering parameters by varying the concentration of stabilizing ligand [211].

Different kinds of carbon nanomaterials can also be considered as a protecting species for NPs since clusters prepared by sputtering onto them can easily absorb on their surface. This prevents future coalescence of the metal NPs in the host liquid and allows one to get new composite materials which are described in the section 2.3.6.1. Here are several examples: Pd, Pt, and PdAu NPs stabilized on carbon nanotubes [212–213], AuPd NPs on graphene sheets [136], Au NPs on graphite surfaces [153], and Pt and NPs on powdered carbon black [168].

**2.1.2.3 Volume of the host liquid:** The volume of the host liquid, more precisely the thickness of the liquid layer, is a parameter that might indirectly affect the properties of the final NPs. In the case of prolonged plasma treatment onto a very thin layer of liquid, its surface can be significantly heated, which might increase the size and size distribution of the particles. Even if we exclude the temperature influence, it is worth mentioning that depositing metal onto a thin liquid layer with high viscosity might create a situation where sputtered species cannot diffuse deeply into the bulk solution, so that a film of NPs will form.

**2.1.2.4 Substrate temperature:** The effect of host liquid temperature on NP properties was studied in [123,127,182,214–217]. Changing the temperature affects surface tension and the viscosity of the host liquid. Thus, it dramatically changes the diffusive velocities of the sputtered species inside the liquid [214]. It was shown that an increase of the substrate temperature for fixed sputtering conditions leads to an increase in both the size and size distribution of formed NPs because the number of collisions between species in the solution during the nucleation and growth steps drastically increases. It was also noticed that the anisotropy of NPs increases with temperature [215]. This might be explained by the coalescence of primary particles due to the weakening of the interactions between metal NP surfaces and host liquid molecules [216].

**2.1.2.5 Viscosity and surface tension of the host liquid:** The conclusion that the NP size depends on the viscosity of the host liquid was reached in [115,123,126–127,134,183,214,216,218–219]. Some of the researchers used ILs with fixed cation [115,183] or anion [134] and tried to find a trend in how the IL viscosity and structure affect NP properties. Others preferred to use the same compound as a host liquid and to control the viscosity by changing the substrate temperature [123,127,214,216]. A common trend was found: “dispersed NPs were formed on liquids with low surface tension and low viscosity whereas dense films were formed on liquids with low surface tension and high viscosity” [218] while the size of NPs (in the solution and inside the films) increases with the viscosity of the host liquid.

Hatakeyama et al. decided to change the viscosity by changing the cation in imidazolium ILs [C*_n_*mim]-BF_4_ (*n* = 2,4,8) [134]. They found that the diffusion velocity of Au NPs is higher in liquids with low surface tension and low viscosity; thus, such liquids are preferable for the generation of small and uniform NPs. Vanecht et al. worked with a line of 1-butyl-3-methylimidazolium (BMIM)-based ILs with different anions [115,183]. They discovered that secondary growth processes take place in the NP solution when the deposition of Au is completed. It was noticed that the kinetics of aggregation and sedimentation depends on the NP diffusion ability and might be controlled by the IL viscosity. They highlighted that when the IL viscosity is too high for immediate penetration by the sputtered species, a metal film forms on the liquid surface rather than a colloidal dispersion of particles [115]. Wender et al. also studied the effect of viscosity on the properties of Au NPs in the line of BMIM-[TFSI, PF_6_, BF_4_, tris(pentafluoroethyl)trifluorophosphate (FAP)] ILs [129]. They did not find a direct correlation of particle size with viscosity or surface tension, but it could be explained by the heating of the host liquid during the sputtering process. Moreover, here, the viscosity is changed by modifying to some extent the chemistry of the host liquid or the temperature. These two parameters may influence NP nucleation and growth.

**Figure 10:**
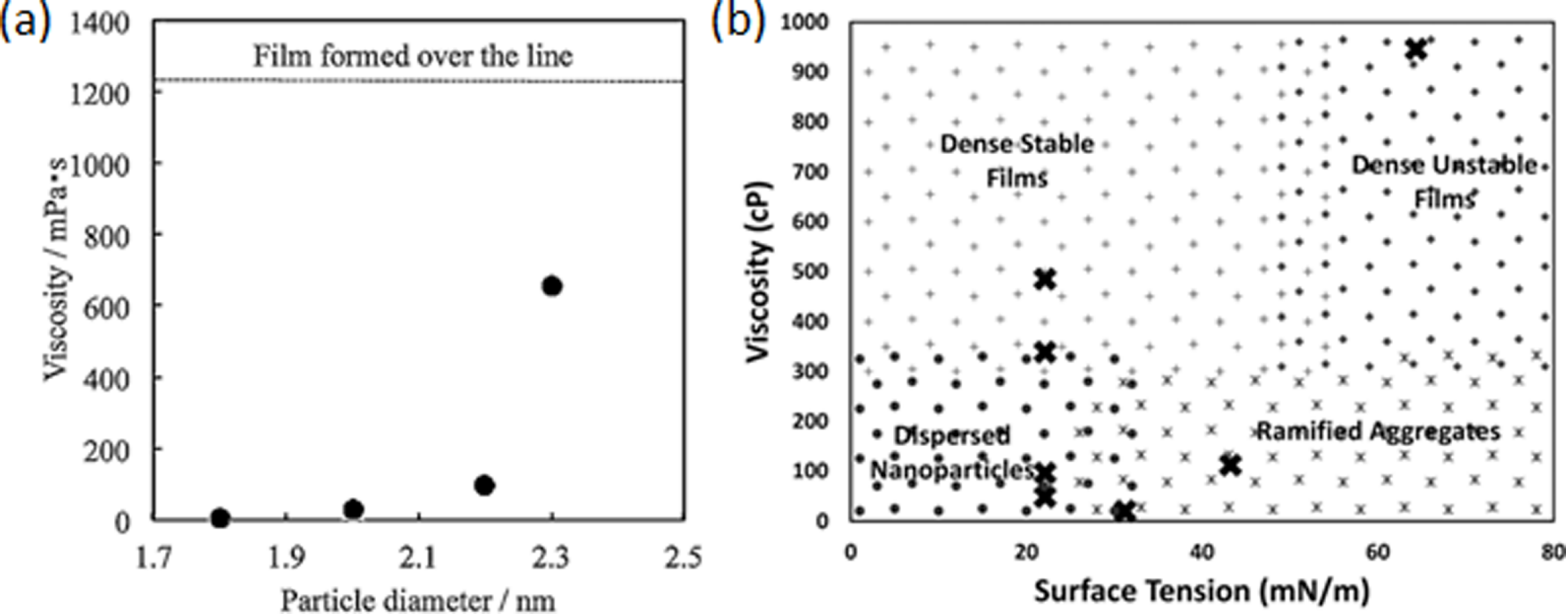
(a) Average diameters of Ag NPs as a function of the viscosity of PEMP controlled by temperature. Figure 10a was reprinted from [123], Colloids and Surfaces A: Physicochemical and Engineering Aspects, vol. 498, by Yohei Ishida, Satoshi Udagawa, Tetsu Yonezawa, “Growth of sputtered silver nanoparticles on a liquid mercaptan matrix with controlled viscosity and sputter rate”, pages 106-111, Copyright (2016), with permission from Elsevier. This content is not subject to CC BY 4.0. (b) Morphological phase diagram of the structures obtained by sputtering Ag and Au on different liquid substrates. Figure 10b was reprinted from [218], with the permission of AIP Publishing. This content is not subject to CC BY 4.0.

It is well known that viscosity is more temperature-dependent than surface tension. The relation between viscosity and temperature can be described by the Andrade equation [220]. This fact was used for controlling viscosity during the sputtering of Au onto C_4_mim-BF_4_ (= BMIM-BF_4_) [214]. It was shown that larger Au NPs can be generated when the viscosity of the IL is lower (high-temperature range). The same observation was made in the case of sputtering of Au onto heated PEG [127,216]. Again, the variation of substrate temperature from 20 to 100 °C during sputtering of Ag onto PEMP allowed for changing the host liquid viscosity and to obtain Ag NPs at low viscosity and thin silver films at high viscosity [123]. It was noticed that the NP diameter increased from 1.8 to 2.3 nm with viscosity and no individual particles were formed at higher viscosity since they could not diffuse inside the bulk solution (Figure 10a). A morphological phase diagram summarizing results obtained by De Luna and Gupta for DC sputtering of Ag and Au over different liquid substrates (Figure 10b) demonstrates that colloidal solutions of NPs form in media with low viscosity and surface tension [218].

The two approaches of viscosity control described above are not perfect. Changing the cation or anion in ILs not only slightly changes surface tension but also leads to a change in IL structure that affects stabilization properties. Changing the substrate temperature affects too many important parameters, that is, it modifies not only viscosity and surface tension of the host liquid but also the kinetics of nucleation and growth processes and the stability of final NPs. In this case, it is difficult to tell what exactly controls shape, size, and size distribution of NPs.

**2.1.2.6 Ionicity of the host liquid:** Although the authors of at least half of the SoL-related publications used ILs as substrate, there was no attempt to link the ionicity of the ILs with the size and stability of the obtained NPs. The “ionicity” parameter is a measure of the degree of dissociation of the cation and anion couple. It depends on the magnitude and balance of the intermolecular forces and correlates with different physicochemical properties of the solvent [221]. Ionicity can be qualitatively measured by different approaches that are summarized in [221]. For example, for [C_4_mim]-based ILs having different anionic structures, the ionicity increases as follows [NTf_2_ = TFSI] < [trifluoroacetate (tfa)] < [trifluoromethanesulfonate (TfO)] < PF_6_ < BF_4_. For [C*_n_*mim][NTf_2_], whilst the ionicity decreases with increasing the length of the alkyl chain (*n*) [221]. Basically, the ionicity is lower in the case of strong associations between cation and anion of the IL. For example, it was shown that in the case of the colloidal synthesis of Ru NPs, the NP size distribution becomes broader and the tendency to agglomerate increases when the ionicity is increased for series of phosphonium and imidazolium ILs [222]. We have analyzed the data from reports dealing with the sputtering of a given target over different ILs and found out that the size and the size distribution of Au NPs increased with increasing IL ionicity (decreasing length of alkyl chain) from 2.4 ± 0.6 nm for OMI-BF_4_ (*n* = 8) to 2.9 ± 0.8 nm for EMI-BF_4_ (*n* = 2) [126]. The size of Au NPs was smaller in [C_4_mim]NTf_2_ (low ionicity) than in [C_4_mim]BF_4_ (high ionicity): 2.0 ± 0.4 nm vs 2.6 ± 0.4 nm in [180] and 5.1 ± 0.8 nm vs 6.5 ± 0.7 nm in [181]. The same trend was observed for Ag NPs [185]. Also, post-sputtering diffusion-limited growth of Au NPs and the tendency to coagulate decrease with increasing ionicity (and increasing viscosity) in imidazolium ILs [115,183]. Thus, ionicity of ILs might be an important parameter in case the production of smallest NPs is desired.

**2.1.2.7 Stirring the host liquid during the deposition process:** Sputter deposition onto still liquids (without stirring) might lead to the formation of NPs with a broad size distribution due to slow diffusion of sputtered species into the solution and a big number of collisions between growing particles (in the overheated top layer). This problem might be solved by sputtering onto running liquids [161] or by introducing a stirring mechanism inside the vacuum chamber [211]. Yonezawa’s group has shown that stirring of the host liquid during sputtering of Au onto PEG helps to decrease the size of final Au NPs by almost a factor of two, from 7.4 ± 2.1 nm to 3.7 ± 0.9 nm [207]. Stirring was applied in [121,127,137,140,199,202–203,205–208,210,223–224]. It was noticed that an increase in the rotation speed leads to a decrease in the NP size and size distribution [127]. Thus, the introduction of stirring is especially important when a big volume of host liquid is used over long periods of sputtering time since stirring can prevent the surface from overheating and thus help to obtain particles with a narrower size distribution.

**2.1.3 Practical recommendations:** The analysis of the effects of sputtering and host liquid parameters has shown that the following factors must be controlled for obtaining reproducible results in SoL processes: (i) the flux of sputtered material (fixed power, current, voltage, working pressure, working distance, and target temperature), (ii) host liquid composition, (iii) substrate temperature, and (iv) the thickness of the host liquid layer and sputter time must be considered to avoid overheating of the surface.

The following recommendation can be made to prepare smaller NPs with narrower size distribution at fixed sputtering parameters: (i) usage of host liquids having a lower viscosity at room temperature, (ii) adding a ligand with high affinity to the sputtered material, and (iii) stirring the reaction mixture during the sputtering process.

#### 2.2 Mechanism of nanoparticle formation during the SoL process

SoL is considered as a unique method to prepare NPs. One of the most valuable advantages of this method is the chemical purity of the obtained NP solution. Additionally, this technique is versatile as it allows for the usage of a quite broad range of materials as sputtering targets [13]. The physics behind the SoL is the same as when creating thin films by PVD, the “only” difference is that the substrate is not a solid material but a liquid solution. Nowadays, the most reported media for the sputtering technique are ILs and PEGs [225]. Other liquids such as PEEL, silicon, and vegetable oils can be found as well in the literature (Figure 9).

A detailed mechanism of the formation of the NPs by SoL can be described as follows. The first part of the NP formation underlies the physical side associated with the PVD process, as described previously in section 1.2. The second part is related to a chemical side of the process associated with the colloidal synthesis described in section 1.3. Plasma ions bombard the negatively biased target and cause sputtering of the target atoms. After traveling through the chamber, the sputtered species reach the liquid surface. Then, diffusion on the surface or inside the liquid is expected. As the delivery of sputtered atoms continues, the local concentration of deposited atoms increases, and hence nucleation and coalescence of the sputtered species occur, ultimately resulting in the formation of NPs [124]. Besides the extended knowledge available on colloidal synthesis, the chemical reactions taking place between the sputtered species and the vacuum-proof liquids are still under investigation [13–14]. Indeed, the growth conditions of the NPs differ to some extent from the classic environments of colloidal synthesis. For example, the total concentration of the sputtered material in the host liquid is not constant such as in typical bottom-up approaches and linearly increases with the sputter time. The sputtered atoms, as well as the other plasma-borne species (electrons, photons, metastable atoms, ions, and radicals) may interact with the liquid surface and locally induce “uncontrolled” chemical reactions and heating. It is important to stress here that the presence of O_2_ molecules can be controlled, in the case of reactive sputtering, or cannot, if the vacuum level is not low enough. The magnitude of these “perturbations” and the growth of the NPs depend on the plasma but also on the physicochemical properties of the host liquid (e.g., surface tension, viscosity, coordination and stabilization capability, and heat capacity), which may vary substantially from one liquid to another. Therefore, it is difficult to know where the two main processes, that is, nucleation and growth, occur and what the characteristics of the obtained NPs, such as size, shape, or elemental composition, will be. Nevertheless, three growth mechanisms are accepted so far, including (i) the nucleation at the liquid surface followed by the diffusion into the liquid phase where the growth of NPs takes place; (ii) both processes occur at the liquid surface; or (iii) the sputtered species penetrate just below the liquid surface and both processes occur in the bulk liquid phase [13]. These hypotheses are supposed to explain both the formation of NPs and thin films observed in the literature [124].

Regrettably, little attention has been paid to these parameters in the first reports, and the deposition conditions were not described in detail, making any reasonable comparison between published results difficult. Also, the conditions applied were very different, and the results cannot be correlated because the formation mechanism can be sensitive to the parameters, such as, for example, the discharge voltage [124]. Consequently, some reports seem to be in contradiction [12,115,124–125,128,134,183]. Overall, the appearance of a particular growth behavior will be strongly and synergistically influenced by the sputtering conditions and the liquid properties. In other words, in one case, under specific sputtering conditions, the NPs could grow on the liquid surface, resulting in the formation of anisotropic NPs [128] or thin films [124,226] and, in other cases, they could grow in the bulk liquid phase [114–115,134].

Besides the confusing situation concerning the effect of the parameters on the formation of the NPs by SoL, it seems that there is a consensus regarding the nature of the host liquids, which plays a predominant role in controlling the NP formation. Indeed, regardless of the operative processes, the NP formation will strongly depend on the interaction between liquid surface and sputtered species. Depending on the affinity between the sputtered material and the liquids, one can form either NPs stabilized in the liquid or aggregates of NPs up to the observation of a thin film at the surface.

Readers interested in the stabilization mechanisms of NPs in liquid media, and in particular in ILs, should refer to the articles of Wegner and Janiak [227] or He and Alexandridis [228]. Generally, the stabilization of NPs occurs through electrostatic or steric stabilization, or a combination of these effects. Stabilization is synonymous to preventing NPs to interact and to prevent their agglomeration due to attractive van der Waals forces. The classic Derjaguin–Landau–Verwey–Overbeek theory (DLVO) was developed to quantitatively describe the electrostatic stabilization of colloidal suspensions by considering repulsive Coulomb and attractive van der Waals forces. If two particles surrounded by long alkyl chains approach each other, the chains will be compressed. This results in a repulsion which is termed as steric stabilization. For ILs, which are often used by research groups working with SoL processes (Figure 9), the stabilizing action can be sometimes twofold and considered as electrosteric. Indeed, besides the interparticle electrical repulsion induced by the cations and anions of the IL interacting with the surface of the NPs (formation of an ionic double layer), steric stabilization can also be promoted if the cation contains a long alkyl side chain. The interaction of cations and anions of ILs with the NP surfaces is a rather complex phenomenon and stems from a balance between electrostatic, van der Waals, and H-bond interactions. Knowing about the coordinative ability of ILs to NP surfaces helps to precisely tune the NP size through sputtering onto a mixture of ILs [188].

**Figure 11:**
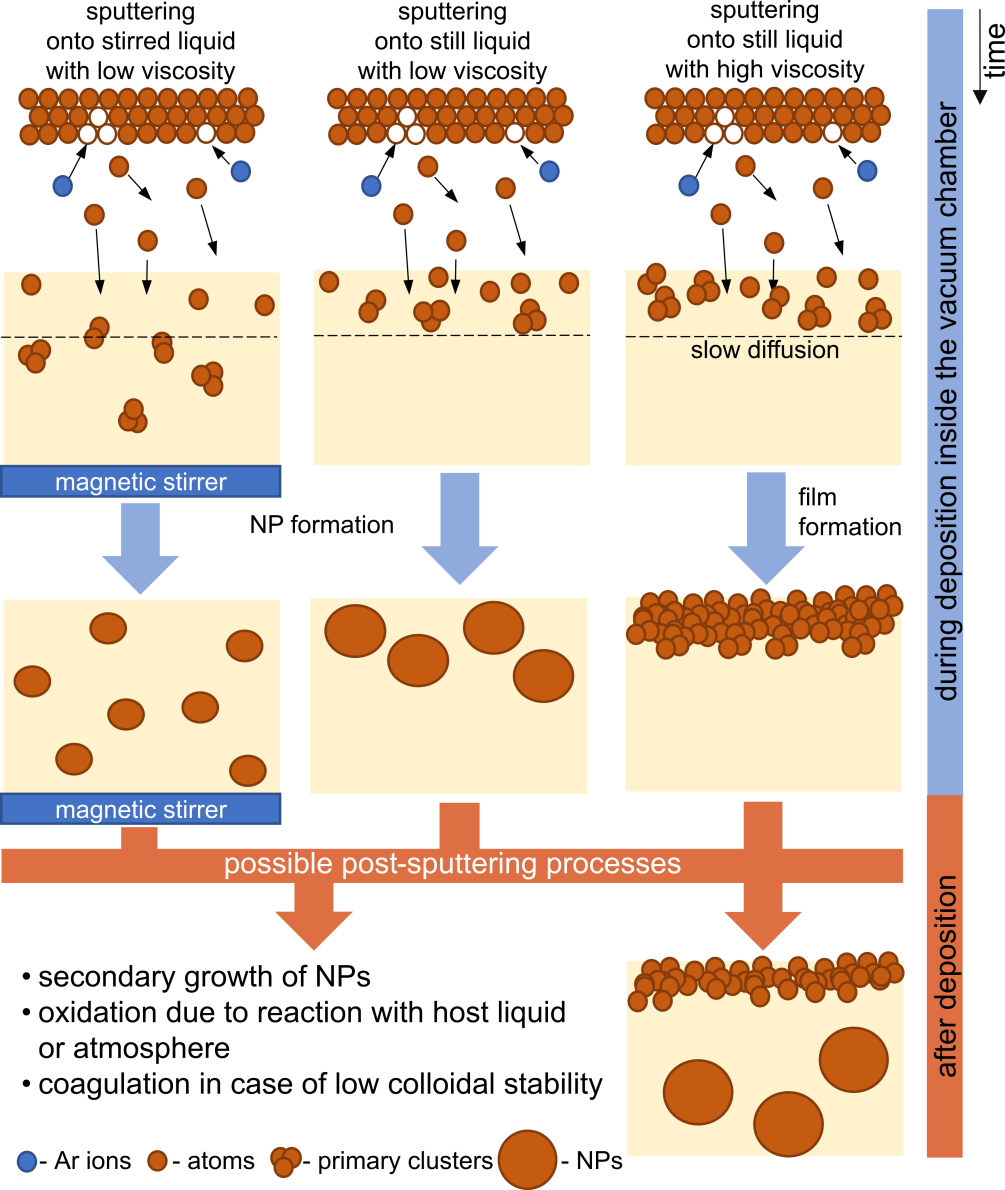
Schematic representation of NP and film growth during the SoL process.

Besides the general overview, the control of the growth kinetics by the liquid environment (surface or bulk) is still a topic of discussion. More experimental data are needed to fully understand the interactions between the host liquids and the sputtered species. In our opinion, NP formation depends mostly on the chemical interactions between sputtered species and the host liquids, the kinetic energy of sputtered species, and the solvent viscosity. The latter may change with temperature and determines the diffusion rate of sputtered species in the solution. If the kinetic energy of sputtered atoms is high enough, they penetrate the liquid surface and cause the heating of the liquid top layer (Figure 11). The formation of the first small clusters, i.e., nucleation, probably occurs in this overheated layer. The subsequent NP growth takes place in the bulk solution [115,132,179,219]. In the case of sputtering on a stirred liquid, there is no gradient of the concentration of the sputtered material in the solution. The solution is homogenized and the formed cluster collide with each other in the whole liquid volume, leading to the formation of small NPs with a narrow size distribution [127]. In the case of sputtering on a still liquid, the concentration of the sputtered material is inhomogeneous. Due to slow diffusion, the concentration of sputtered species is higher at the top liquid layer, which is hotter (Figure 5) and less viscous than the liquid bulk (Figure 11). Since more collisions between the initial clusters occur in the top part of the solution, bigger particles with broader size distribution form in contrast to sputtering onto a stirred liquid [211]. If the viscosity of the host liquid is very high, the particles formed in the top layer cannot diffuse into the bulk solution. This situation leads to the formation of a thin film [123].

Finally, secondary post-sputtering processes might also occur in the solution after the deposition (Figure 11). For example, in some cases, the oxidation of the sputtered species is observed inside the host liquid because of the chemical nature of the liquid itself and/or the presence of oxygen and water dissolved in it [174,189]. The initially formed film might react with the host liquid leading to the formation of NPs [179]. Aggregation of NPs is observed in the case of low colloidal stability due to weak stabilization of NPs by the host liquid [131,164]. Secondary growth processes might also take place in the solution [115,132,185]. So, ideally, the NP size should be characterized after the deposition and after several weeks of the storage.

#### 2.3 Materials produced by sputtering onto liquids

**2.3.1 Monometallic NPs:** A wide range of elements has been successfully sputtered onto liquids to create monometallic NPs, including Au [12,114–115,118,120,122,125–126,129,132,134,138–139,142–143,145–149,153,167,174–176,180–183,188–189,203–204,207–208,210–211,214,216,218,229–238], Ag [11,118–119,123,131,164,175,179,184–185,188,196,205–206,217–218,231–233,239], Cu [150,155,164,188–189,198,210,230,240–241], Pt [121,133,163,168–169,186,242–245], Pd [116,212,231,242,246–247], Fe [11,161–162], In [187], Ni [135] and Mo [201]. Most of the studies reporting the formation of monometallic NPs deal with the influence of synthesis parameters on the formation of NPs. These studies have been reported in section 2.1. Herein, we review the literature dealing with the properties of monometallic NPs obtained by SoL. A large amount of the reported results concerns the catalytic, luminescence, and biological properties. Other properties are also presented, such as the magnetic properties of the NPs and the conductivity of the liquid containing the monometallic NPs.

**Figure 12:**
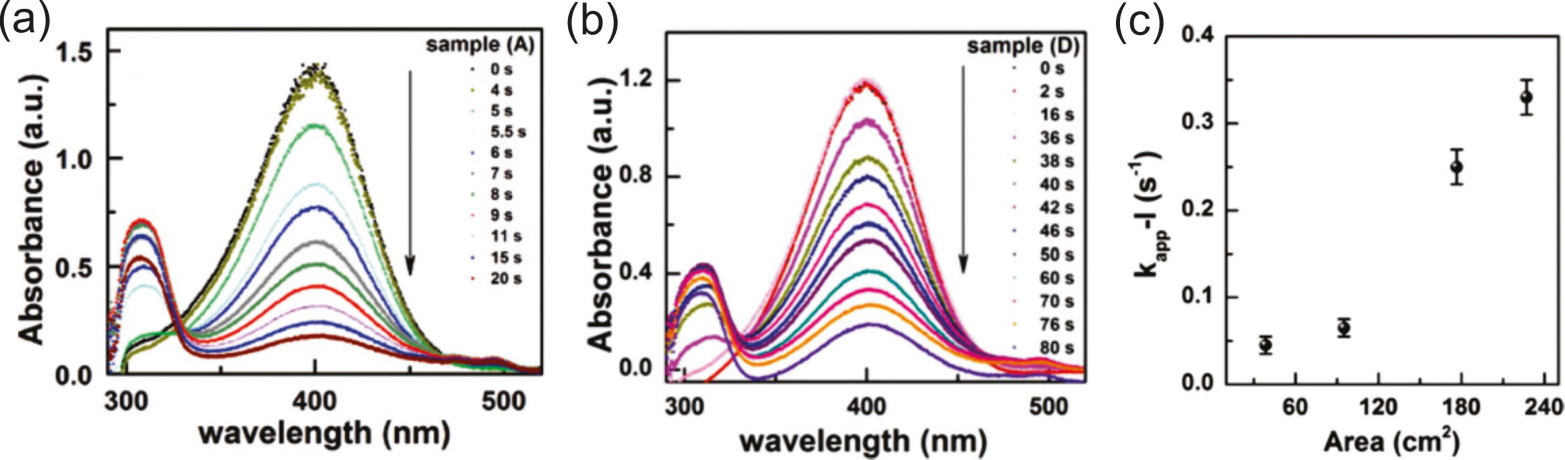
In situ UV–vis spectra for the reduction of 4-nitrophenol using Au NPs in DES solution after sputtering an Au target onto (a) a small and (b) a large surface area of the DES. (c) Evolution of *k*_app_-I as a function of the different DES surface areas. Figure 12 was adapted from [146]. Republished with permission of Walter de Gruyter and Company, from “Gold Nanoparticles in Novel Green Deep Eutectic Solvents: Self-Limited Growth, Self-Assembly & Catalytic Implications”, by O’Neill, M.; Raghuwanshi, V. S.; Wendt, R.; Wollgarten, M.; Hoell, A.; Rademann, K., ZEITSCHRIFT FÜR PHYSIKALISCHE CHEMIE, 229, 1-2, 2015; permission conveyed through Copyright Clearance Center, Inc. This content is not subject to CC BY 4.0.

**2.3.1.1 Catalytic properties:** The literature witnesses the rapid development of nanoscale catalysis for various applications such as oxygen reduction reaction (ORR), low-temperature oxidation of CO, partial oxidation of hydrocarbons (methanol or ethanol), water–gas shift reaction, and reduction of nitrogen oxides [248]. Briefly, in the presence of a catalyst, a reaction can run to completion with less external energy by lowering its activation energy [249]. In the search for more effective catalysts, due to their small size, NPs are playing an important role because of the enhanced reactivities and selectivities as compared to their bulk counterparts [248]. The origin of the higher catalytic activity of the NPs is still not fully understood. However, it is usually accepted that it originates from the presence of low coordination number atoms together with a higher open active surface when the size of the NPs decreases [249–250]. Besides the size, the composition of the NPs is also important to obtain high catalytic activity [251]. In this context, the catalytic properties of pure Pt and Pd NPs obtained by SoL have been first revealed by the group of Torimoto [133,246]. Pt NPs were obtained by sputtering a Pt target over ILs. The benefit of the aggregation of Pt NPs on the catalytic activity toward the ORR was highlighted. The aggregation of NPs was observed when increasing the temperature of the solution [133]. The catalytic reactions were optimized for two IL/NP suspensions under biphasic conditions [246]. Besides, Pd NPs successfully catalyzed the Suzuki–Miyaura reaction coupling of hydrophobic and hydrophilic aryl halides in water [246]. More recently, Cano et al. studied the catalytic properties of small Pd NPs (from less than 1 to 3 nm) obtained by sputtering onto different ILs. Besides the small size of the NPs, they reveal the importance of the dynamic behavior of the surface atoms controlled by the environment. More particularly, Pd NPs in ILs containing [NTf_2_] anions reveal an increasing of catalytic activity by a factor of two for the cyclopropanation of alkenes, as compared to compact NPs of similar size [247]. Then, the group of Radermann probed the catalytic activity of Au NPs obtained by SoL for the reduction of 4-nitrophenol [146]. In this study, Au NPs were synthesized by sputtering over a deep eutectic solvent (DES), namely a mix of choline chloride and urea. The catalytic activity of the Au NPs was reported to be higher (with a conversion reaction rate constant *k*_app_ = 0.34 s^−1^) than the one reported in the literature for Au NP networks (*k*_app_ ≈ 0.030 min^−1^) [146]. Moreover, they revealed the increase of catalytic performance for the reduction of 4-nitrophenol for non-self-assembled NPs as compared to self-assembled ones (Figure 12) [146]. The catalytic properties of monometallic NPs made by the SoL process have not been extensively studied. As for monometallic NPs, only a few studies report the catalytic properties of alloy NPs. The catalytic properties of alloy NPs are discussed in section 2.3.2. The properties of NPs supported by various nanostructures raised more interest [248]. Thus, more works in the field of SoL has been carried out towards the use of such NPs supported on (nano)materials (i.e., carbon nanostructures, metal, or metal oxide). An overview of the results concerning the combination of NPs prepared by the SoL approach and carbon, metal, or metal-oxide structures is developed in more details in section 2.3.6.

**Figure 13:**
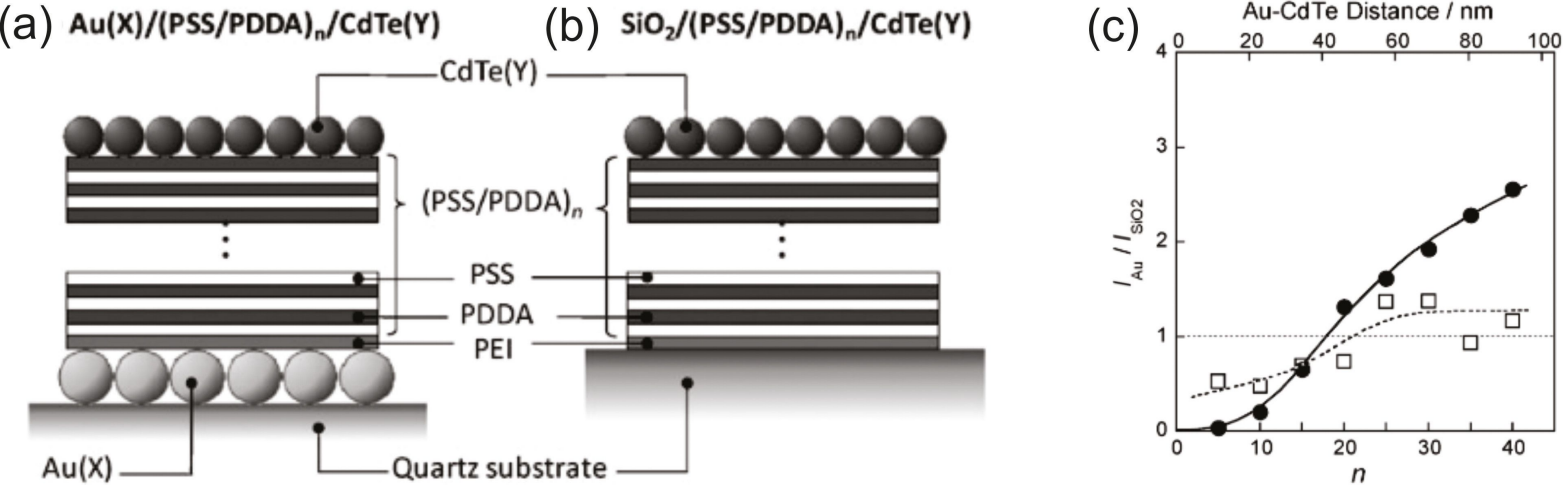
Schematic illustration of multilayer films made of (a) AuNPs/[poly(sodium 4-styrenesulfonate) (PSS)/poly(diallyldimethylammonium) (PDDA) chloride)]*_n_*/CdTe and (b) SiO_2_/(PSS/PDDA)*_n_*/CdTe films. (c) Dependence of the PL enhancement factor of CdTe on to the number of PSS/PDDA spacer layers (*n*) and for small Au NPs (open triangles) and large Au NPs (solid circles). Figure 13 was republished with permission of The Royal Society of Chemistry, from [176], (“Size control and immobilization of gold nanoparticles stabilized in anionic liquid on glass substrates for plasmonic applications” by T. Kameyama et al., in Physical chemistry chemical physics: PCCP, 12, 8, 2010); permission conveyed through Copyright Clearance Center, Inc. This content is not subject to CC BY 4.0.

**2.3.1.2 Luminescence properties:** Due to their small size, clusters (ca. 1 nm large NPs) reveal fascinating photoluminescence (PL) properties for applications in fields such as chemical sensing, bio-imaging, cell labeling, phototherapy, and drug delivery [252]. These clusters are considered to have characteristics lying between those of single metal atoms, which show discrete optical properties, and those of metal NPs, which show plasmon absorbance bands [211]. Monometallic clusters obtained by SoL indeed exhibit luminescence properties. For more detailed information regarding the PL properties of the NPs obtained by SoL, the reader may consult a dedicated review already available [211]. First, the team of Torimoto et al. produced a layer of Au NPs that enhanced the PL of the CdTe NPs deposited onto it (Figure 13a,b) [176]. The samples were made by spreading the IL solution containing the Au NPs over an imidazolium-modified quartz surface. The sample was then heated (or not) to tune the NP size and the optical properties of the sample (Figure 13c). The PL enhancement was improved compared to a pure Au NP layer, and the modulation strongly depended on the distance between the CdTe NPs and the Au NPs in the layer [176].

**Figure 14:**
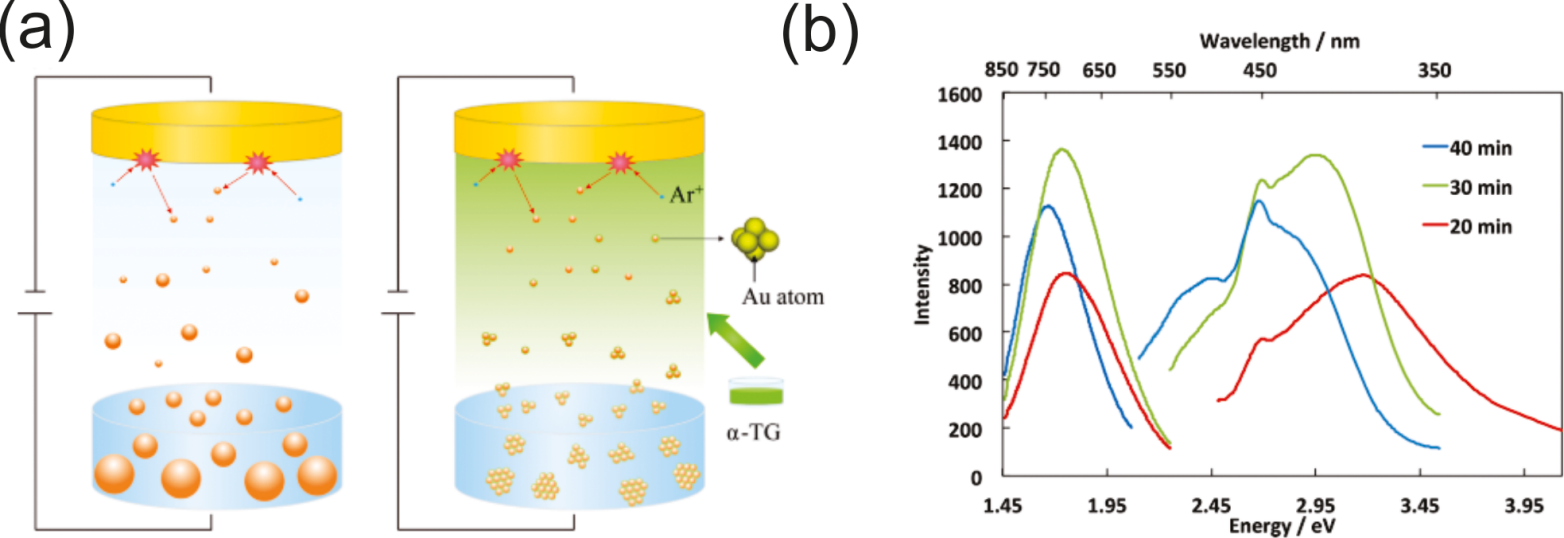
(a) Schematic illustration of the sputtering equipment (left) without α-thioglycerol and (right) with α-thioglycerol in the chamber. (b) Fluorescence and excitation spectra of Au NPs protected by α-thioglycerol in PEG after sputtering at a current of 10 mA and for different times. Figure 14 was adapted with permission from [120]. Copyright 2015 American Chemical Society. This content is not subject to CC BY 4.0.

Later, the team of Yonezawa extensively studied the PL of NPs [120,142,155,202,206,208–210,235]. First, they studied the fluorescence of Au NPs. They highlighted the influence of the solution where the NPs are sputtered and further dispersed [142,202,208,235]. For Au NPs fabricated in (6-mercaptohexyl)trimethylammonium bromide (6-MTAB), they observed a maximum of emission at 770 nm after dispersion in an aqueous medium. However, after dispersion in octadecyl isocyanide, the maximum emission peak moved to 470 nm [142]. In contrast, when the Au NPs were produced by sputtering onto ILs instead of 6-MTAB, the emission maximum was directly observed at 444 nm [235]. Moreover, compared to the previous study [142], a much smaller Stokes shift was reported. The Stokes shift after sputtering onto ILs equals 0.13 eV instead of 2.4 eV after sputtering onto 6-MTAB. The quantum yields (QYs) reported are 10^8^ higher than that of bulk Au and enhanced compared to Au NPs stabilized by thiolated ligands. This behavior is linked to the characteristic properties of IL molecules surrounding the Au NPs [235]. Indeed, after studying the influence of the size of NPs stabilized by thiolate ligands on the fluorescence of the Au NPs [202,208], they observed a NIR fluorescence of the Au NPs with a QY equal to 0.9% [202]. They highlighted that the NPs consisted of aggregates of tiny fluorescent clusters. This behavior is only reported for NPs made by SoL [208]. To increase the QYs of Au NPs obtained by sputtering, they proposed a novel methodology to synthesize small and highly fluorescent Au NPs [120]. By introducing a vapor of α-thioglycerol in the sputtering chamber, they were able to obtain Au clusters in the gas phase at 20 Pa pressure (Figure 14a). The plasmon absorption observed at 520 nm for larger NPs was not observed for the clusters obtained here. Instead, they found a new plasmon absorption peak at around 360 nm. Moreover, the fluorescence maximum of the Au clusters is in the near IR region and redshifts with increasing size of the NPs (Figure 14b). Using this technique, they were able to reach a QY of 16% [120].

**Figure 15:**
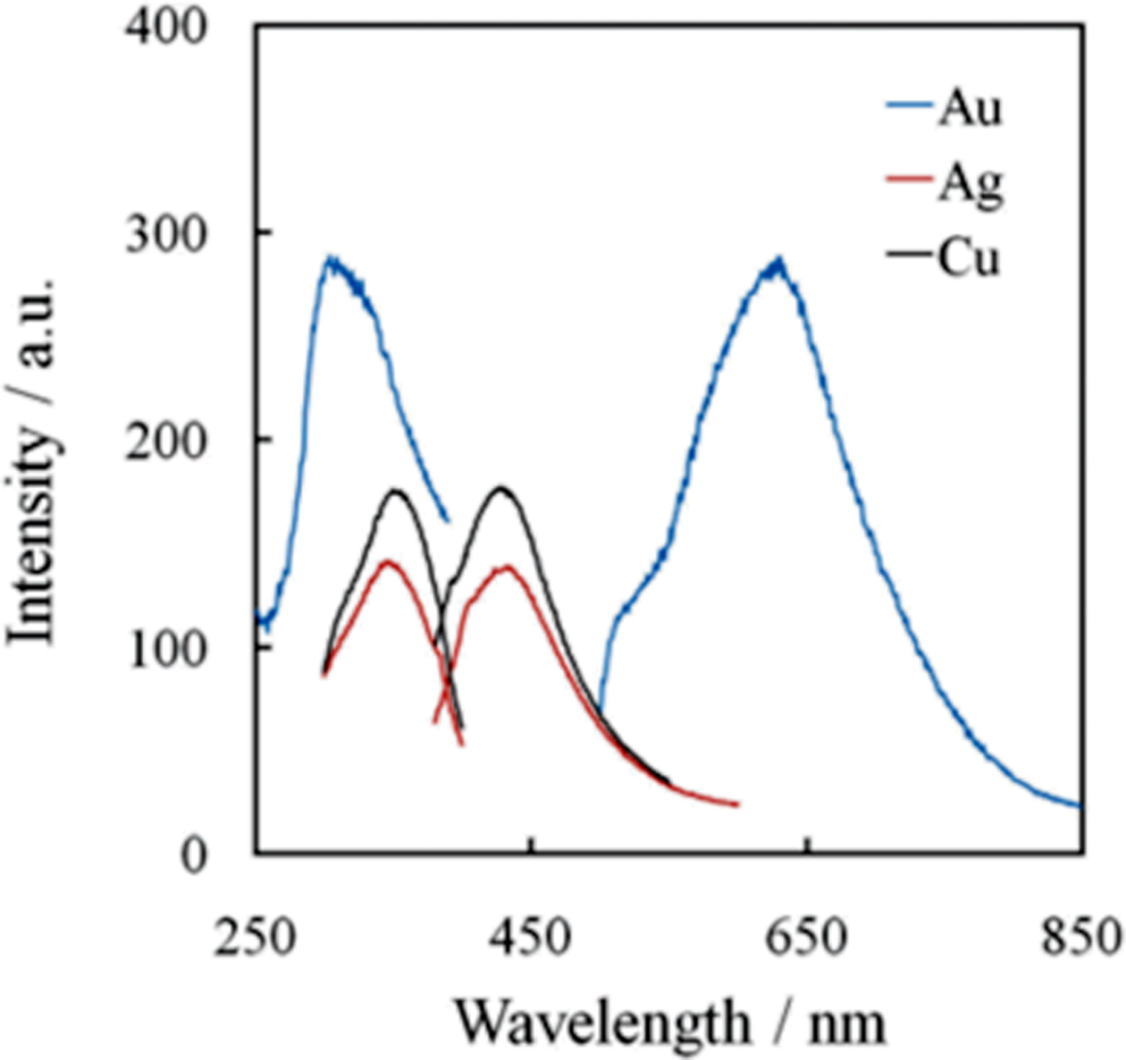
Photoluminescence spectra of Au, Ag, and Cu nanoclusters in (11-mercaptoundecyl)-*N*,*N*,*N*-trimethylammonium bromide (MUTAB)/PEG solution. Figure 15 was republished with permission of The Royal Society of Chemistry, from [210] (“Synthesis of cationically charged photoluminescent coinage metal nanoclusters by sputtering over a liquid polymer matrix”, by R. D. Corpuz et al., in New journal of chemistry, 41, 14, 2017); permission conveyed through Copyright Clearance Center, Inc. This content is not subject to CC BY 4.0.

After having studied extensively Au NPs, Yonezawa’s group further studied the PL of other metal NPs, such as Ag and Cu NPs [155,206,209–210]. The synthesis of the NPs was carried out by sputtering onto ILs in all studies. The Ag and Cu NPs exhibited a size smaller than 2 nm [210]. A blue emission with a maximum located at 432 nm and at 428 nm was reported for Ag and Cu NPs, respectively (Figure 15) [210]. For Cu NPs obtained by sputtering onto PEMP, a shift of the PL emission peak during storage was revealed. They explained this shift by the conversion from Cu NPs to copper oxide (Cu_2_O) NPs and, ultimately, to the formation of copper sulfide (Cu_2_S) NPs. This evolution is linked to the abundance of thiol groups together with the high reactivity of Cu_2_O NPs. Thus, with simple post-processing, they were able to tailor the structure and PL properties of Cu NPs in liquids [155]. Then, they sputtered Cu onto PEG containing MUA molecules. The change in MUA concentration had an impact on the PL intensity but not on the peak position [209]. Finally, the preparation of NIR fluorescent Ag NPs sputtered onto PEG containing anionic thiols (SMPs) was demonstrated. The SMPs were shown to influence the optical properties of the Ag NPs. By increasing the SMP concentration, the plasmonic absorption decreased while the NIR emission increased [206].

**Figure 16:**
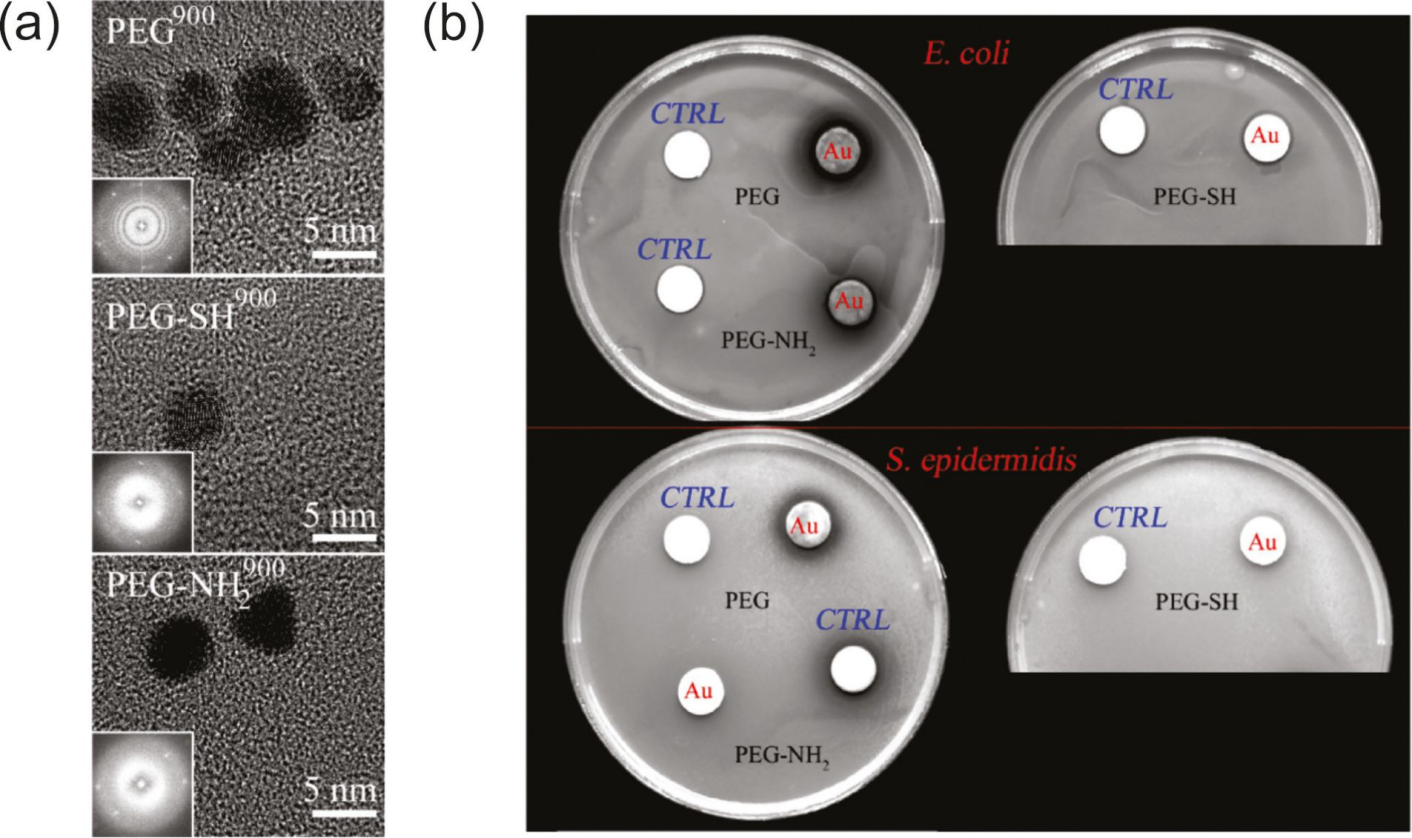
(a) HRTEM images with FFT and (b) photographs of the antibacterial disc of the PEGylated Au NPs prepared by sputtering of Au onto PEG^900^, PEG-SH^900^, and PEG-NH_2_^900^. Figure 16 was reprinted from [139] (Colloids and Surfaces A: Physicochemical and Engineering Aspects, 560, A. Reznickova, N. Slavikova, Z. Kolska, K. Kolarova, T. Belinova, M. Hubalek Kalbacova, M. Cieslar, V. Svorcik, “PEGylated gold nanoparticles: Stability, cytotoxicity and antibacterial activity”, 26-34), Copyright (2019), with permission from Elsevier. This content is not subject to CC BY 4.0.

**2.3.1.3 Biological properties:** The unique biological properties of NPs originate from their specific interactions with selected proteins to inhibit their activities [253]. Among others, the antimicrobial properties of NPs fabricated through the SoL process were studied. The antimicrobial properties strongly depend on the physicochemical characteristics of the NPs and the type of bacteria targeted [254]. First, Hamm et al. provided, via Ag sputtering over ILs, a sacrificial Ag^+^ reservoir for antimicrobial composite coatings [239]. The solution containing the NPs is called “ionosol”. This ionosol was incorporated into a silica-based sol–gel to create a nanocomposite. The antimicrobial activity of the SiO_2_/IL/Ag NPs nanocomposite was evaluated against *Pseudomonas aeruginosa* bacteria. They explained the good bactericidal activity of the coatings by the synergy between all the components. Moreover, they revealed the strong dependence of the bactericidal activity on the type of ILs used during sputtering [239]. Then, the team of Svorcik reported the antibacterial activity of Au, Ag, Pd, and Pt metal NPs prepared by sputtering onto glycerol [233,242]. The antibacterial activity of the NPs was examined against *E. coli* and *S. epidermis*. While the size of the Ag NPs did not influence the inhibition of both bacteria, Au NPs have a more pronounced inhibition capability as their size decreases [233]. Moreover, they reported that the Pd NPs exhibited considerable inhibitory potential against both bacteria, contrary to the Pt NPs, which show no antibacterial activity [242]. In contrast, the antimicrobial properties of NPs can be detrimental for applications such as drug delivery [139]. In this context, the team of Svorcik further reported that the solvation of the Au NPs in thiol- or amine-terminated PEG (PEGylated Au NPs) annihilates the antibacterial effect reported in their previous work (Figure 16) [139].

**2.3.1.4 Electrical and magnetic properties:** Monometallic NPs obtained by SoL have also been investigated for their conductivity and magnetic properties. Hamm et al. used Au NPs sputtered directly onto ILs to significantly enhance the ionic conductivity and capacitance of the liquid host. This property is highly valuable for electric double-layer capacitors (EDLCs). The unique interaction between the IL and the NPs obtained by sputtering has been a significant contributor towards enhancing the electrochemical properties of EDLCs [167]. Cigan et al. also revealed the superparamagnetic properties of Ni NPs sputtered into ILs. The effective magnetic moment of the Ni NPs was higher than that of the bulk counterpart. Moreover, they provided evidence of long-term magnetic stability over 32 months [135].

**2.3.2 Alloy NPs:** The increasing demand for multifunctional materials urges the development of NPs with enhanced properties. One efficient pathway to do so is to fabricate NPs from two or more metals. Using the synergy of the metals, the properties of NPs can be drastically enhanced. In this context, the production of NPs by SoL can be adapted to the synthesis of alloy NPs. Nowadays, a number approaches have been reported, which can be classified by the number of targets used simultaneously: (i) the single target sputtering approach or (ii) the multitarget approach (also called co-sputtering, Figure 3b). For more detailed information regarding the creation of alloy NPs by SoL, a dedicated review is available [14].

**Figure 17:**
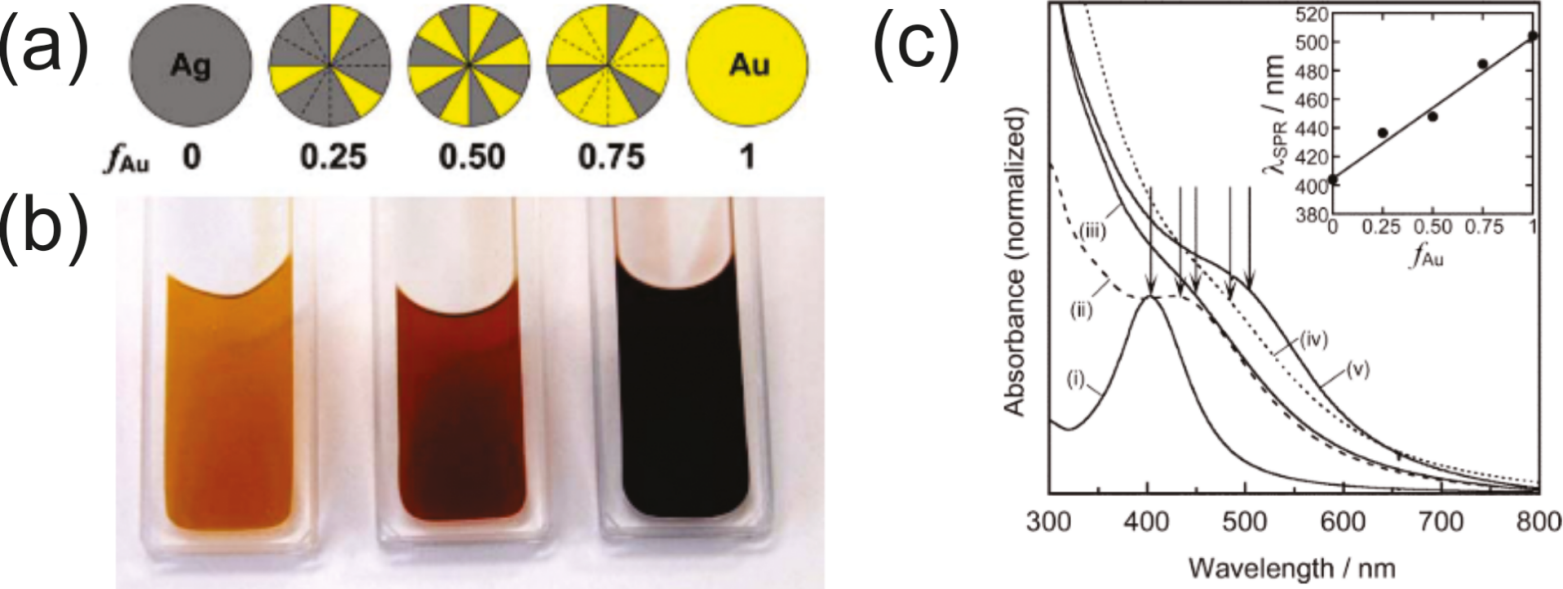
(a) Schematic illustration of Au/Ag binary targets with different gold fractions (*f*_Au_). (b) Photographs of the IL solutions obtained after the sputtering of (left) Ag, (middle) Au/Ag with *f*_Au_ = 0.5, and (right) Au. (c) Normalized absorption spectra of IL solutions after sputtering of Au/Ag binary targets with *f*_Au_ = 0 (i), 0.25 (ii), 0.50 (iii), 0.75 (iv), and 1.0 (v). The arrows highlight the SPR band peak for each solution. (Inset) The dependence of the peak wavelength of the SPR on *f*_Au_. Figure 17 was republished with permission of The Royal Society of Chemistry from [194] (“Single-step synthesis of gold-silver alloy nanoparticles in ionic liquids by a sputter deposition technique”, by K. I. Okazaki et al., in Chemical communications, Vol. 2008, Issue 6, 2008); permission conveyed through Copyright Clearance Center, Inc. This content is not subject to CC BY 4.0.

**2.3.2.1 Single target sputtering approach:** The first configuration uses a single target composed of two metal elements, that is, a bimetallic target. The first reported study using this configuration was made by the team of Torimoto. They performed the synthesis of Au/Ag alloy NPs using a sectored Au/Ag binary target (Figure 17) [194]. Varying the proportion of each element in the target enables the preparation of bimetallic alloy NPs with different compositions. The composition of the individual alloy NPs was probed by STEM-EDS after sputtering and compared to the SPR peak maximum of the solution. A linear correlation between the shift in the SPR signal and the Au content in the target was reported (Figure 17c). The setup using sectored targets has been extended to produce Au-based alloy NPs including Au/Pt [255], Au/Pd [152,190], and Au/Cu NPs [215]. These studies reveal the versatility of this approach to create alloy NPs for various bimetallic systems. For instance, immiscible alloys such as Au/Pt NPs can be easily synthesized as compared to chemical synthesis, which is complex. Another approach, not yet reported for SoL area, is to insert plugs made of a material A into the racetrack of a sputtering target made of a material B. The number of plugs inserted inside the target racetrack allows for controlling the A/B atom ratio.

**Figure 18:**
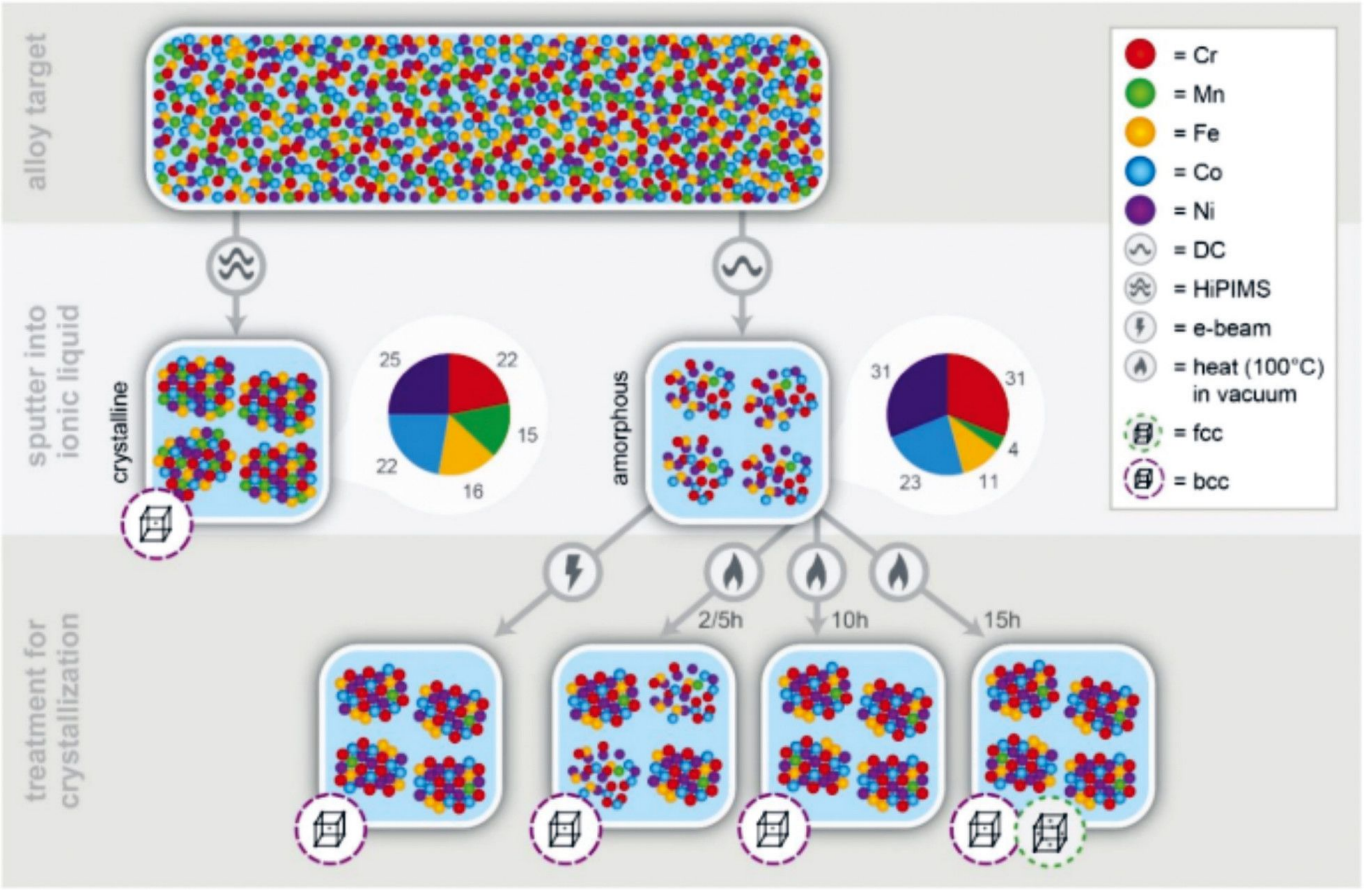
Schematic overview of the different routes used to synthesize amorphous and crystalline Cr/Mn/Fe/Co/Ni NPs. Figure 18 was reproduced from [173] (© 2018 A. Garzón-Manjón et al., distributed under the terms of the Creative Commons Attribution 4.0 International License, https://creativecommons.org/licenses/by/4.0).

The second configuration relies on the use of already alloyed targets instead of the abovementioned segmented bimetallic targets. This setup has been used mostly by the team of Wang. A first example is the sputtering of Au/Pd alloy targets with various compositions to synthesize Au/Pd NPs. The authors report a good correlation between the Au/Pd target composition and the composition of the NPs [193,213,256]. Similarly, they produced Pt/Ni alloy NPs by sputtering a Pt/Ni alloy target. In this case, the sputtering of a Pt-rich target was mandatory to produce a composition ratio of 1:1 in Pt/Ni alloy NPs [257]. The researchers monitored the composition of the NPs by STEM-EDS and inductively coupled plasma atomic emission spectroscopy (ICP-AES). More complex alloy NPs can also be synthesized through this method, such as Cr/Mn/Fe/Co/Ni NPs (Figure 18). The composition of the NPs, reported by STEM-EDS, shows that the concentration of Cr and Ni within individual NPs was varied to a large extent from one NP to another while the concentration of Mn, Fe, and Co remained relatively constant for every NP probed. When probing the overall composition of the NPs by inductively coupled plasma mass spectrometry (ICP-MS), the composition of the NPs was in good agreement with the composition from STEM-EDS analysis for most of the elements except for Cr and Ni. The discrepancy is explained by the difference in the techniques used for probing the composition. While, STEM-EDS is local (i.e., individual NPs are probed), the ICP-MS analysis is global and averages over all NPs present in the solution [173]. Later the authors highlighted, supported by HRTEM and STEM-EDS data, that the composition, the size, and the crystallinity of the NPs can differ substantially when using the same alloy target but different ILs [258]. Compared to a bimetallic target, using an alloy target is not advantageous for tailoring the NP composition. Indeed, varying the surface area of different metal sectors on a target remains easier than preparing a new alloy target. Moreover, it was noticed that this approach was not adapted for the synthesis of immiscible metals to make an alloy NPs.

**Figure 19:**
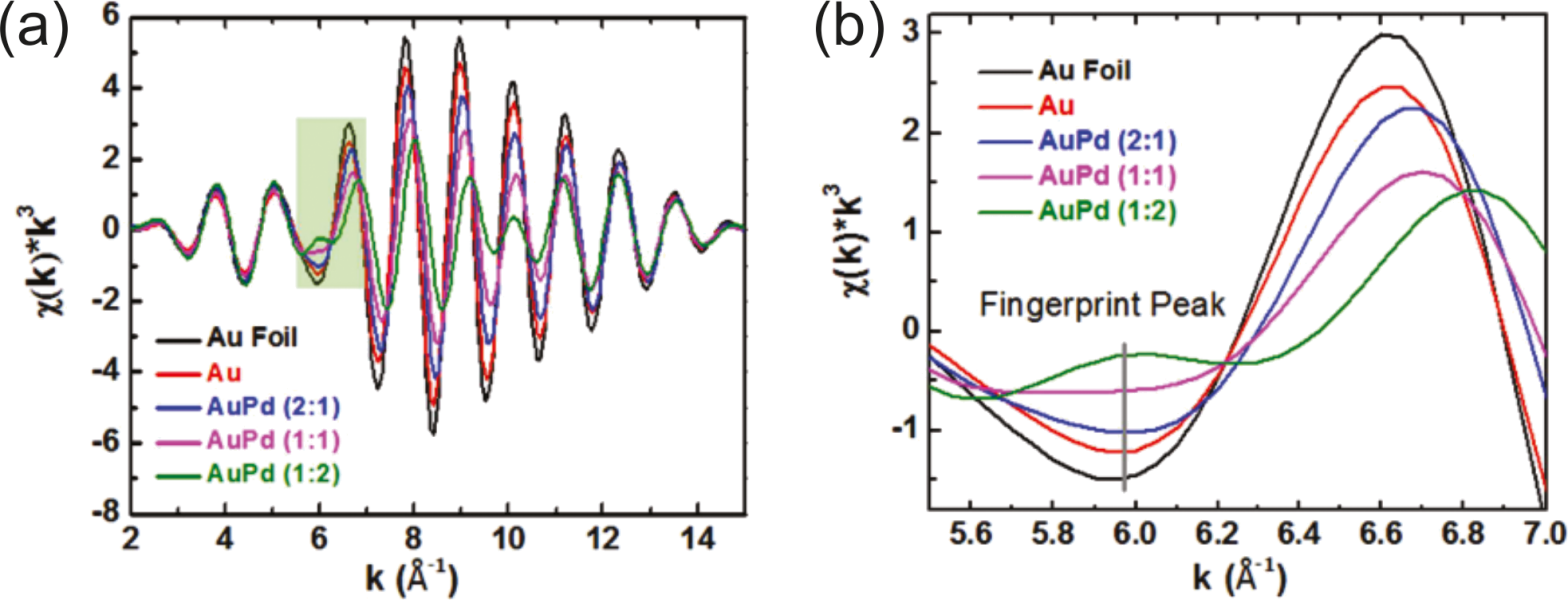
Au L_3_ EXAFS data for an Au metallic foil (reference) and Au/Pd alloy NPs with different Au/Pd ratios. (a) *k*^3^χ(*k*) from the backward Fourier transform and (b) zoom in a sub-range *k* region showing the fingerprint of the different alloy NPs. Figure 19 was adapted with permission from [136]. Copyright 2017 American Chemical Society. This content is not subject to CC BY 4.0.

The third setup is the sequential sputtering of monometallic targets. In this configuration, the synthesis of alloy NPs is based on the sputtering of one target, followed by the sputtering of a second target. This approach may eventually lead to the creation of core@shell NPs instead of alloy NPs and is therefore not the most appropriate if the synthesis of homogeneous alloy NPs is the focus of the work. More details about the synthesis of core@shell NPs can be found in the next section. Nevertheless, a successful example of alloy synthesis using this setup was reported by the team of Wang, who synthesized alloy Pd/Au NPs by the successive sputtering of Pd and Au targets. In particular, they evidenced the presence of both elements in a single NP with high-angle annular dark-field scanning TEM (HAADF-STEM) line profiles and by the shift of the X-ray diffraction (XRD) peaks [192]. Moreover, the extended X-ray absorption fine structure (EXAFS) measurements further revealed that the successive sputtering of Au and Pd targets leads to the creation of Au/Pd intermixed alloy NPs (Figure 19) [136]. In this setup the overall composition of the obtained Au/Pd alloy NPs is modified by tuning the sputtering time at each step. For example, if the NPs are synthesized with a Au/Pd ratio of 1:1 (equal time of sputtering of Pd and Au), the NPs composition, probed by STEM-EDS, ranges from 34 to 69 atom % Au for the same solution [136,191–192].

Moreover, the high versatility of SoL towards alloy NPs synthesis has been revealed by producing trimetallic NPs from the immiscible Au/Pt/Pd system [213]. To do so, the team of Wang used the sequential sputtering of a Pd/Au alloy target and a Pt target. The STEM-EDS analysis shows that the three elements form alloy NPs. Also, the global composition of the NPs present in the solution after the plasma treatment, probed by ICP-AES, correlated well with the sputtering time ratio and the alloy composition of each target [213]. Such an example of the formation of ternary noble metal alloy NPs highlights that sputtering has made significant progress in achieving extended control of the structure and composition of metal NPs.

Another method for synthesizing alloy NPs is by combining sputtering and galvanic reactions to create bimetallic Au/Ag alloy NPs [113]. Using this approach, the team of Torimoto sputtered an Ag target onto ILs containing HAuCl_4_ salt. Consequently, the sputtered Ag atoms and clusters acted to reduce Au ions via a galvanic reaction. The subsequent alloying of Au and Ag occurred and resulted in Au/Ag alloy NPs. The successful creation of alloy was highlighted by the observation of the SPR of Au/Ag alloy NPs. The alloy NPs composition was manipulated simply by varying the concentration of HAuCl_4_ in the IL [113]. This synthesis approach can be extended to various bimetallic systems since the galvanic reaction can be applied to other pairs of metals.

**Figure 20:**
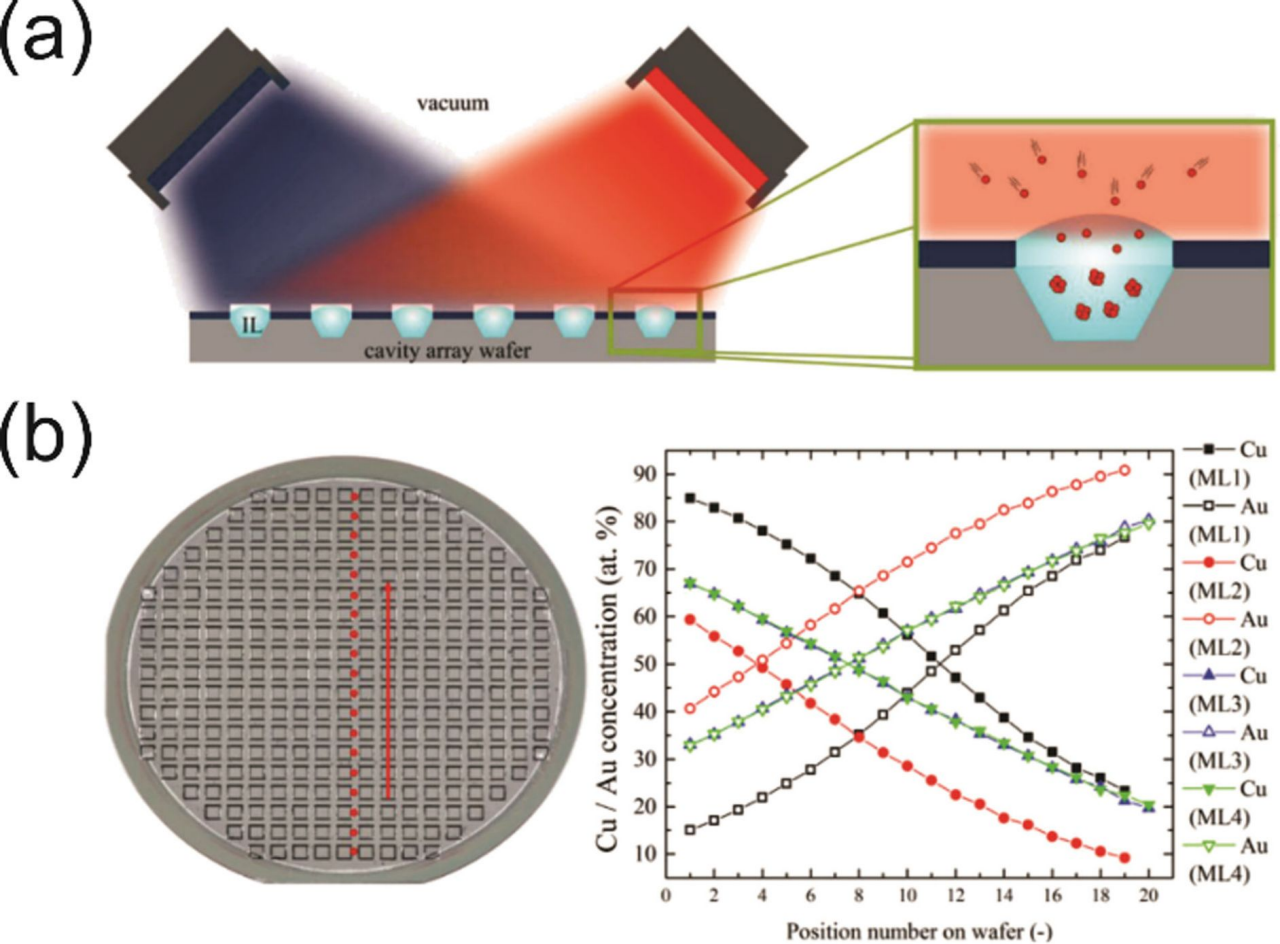
(a) Left: schematic of the co-sputtering process into a cavity array substrate filled with an IL. Right: Schematic of the proposed formation process of NPs in an IL. (b) Left: Photograph of the cavity array substrate. The red dots illustrate the measurement points, whereas the red arrow represents the direction in which the EDS line scan analysis was performed. Right: Results of the EDS screening for four material libraries. Figure 20 was reprinted from [259], D. König et al. “Throughput Fabrication of Au–Cu Nanoparticle Libraries by Combinatorial Sputtering in Ionic Liquids", Adv. Funct. Mater., with permission from John Wiley and Sons. Copyright © 2013 WILEY-VCH Verlag GmbH & Co. KGaA, Weinheim. This content is not subject to CC BY 4.0.

**2.3.2.2 Multitarget sputtering approach (co-sputtering):** In the first studies dealing with the synthesis of alloy NPs via SoL, single targets or sequential sputtering setups were mainly used. However, such setups require fabricating different targets to vary the composition of the resulting NPs. Moreover, there is also a limitation related to the composition of the target and the composition of the obtained NPs. This composition may vary because of different atom transport efficiencies in the gas phase, for example. It is also challenging to create alloy NPs using sequential sputtering since most NPs will exhibit a core@shell structure. To overcome these issues, the simultaneous sputtering of multiple targets, a process referred to as co-sputtering, is an appealing alternative [259]. This approach has gained substantial interest recently since most of the papers report the synthesis of alloy NPs by co-sputtering onto liquids (co-SoL). From a fundamental point of view, co-SoL provides more freedom in forming binary structures of NPs. In 2014, Ludwig et al. reported the synthesis of binary alloy NPs by co-sputtering Cu and Au targets [259]. The sputtering was done onto an array of small cavities (small compared to the target to substrate distance) containing an IL (Figure 20a). The sputtering of the two separated targets over a static substrate resulted in the formation of Au/Cu NPs with location-dependent composition ranging from Au-rich to equimolar Au/Cu to Cu-rich NPs (Figure 20b). It is important to stress here that the composition was probed by EDS analysis on the substrate plate holding the cavities and thus by measuring the composition of the thin film deposited on the plate between each cavity. Hence, the presented composition does not correspond to the NP composition. This approach is attractive since a wide range of NP compositions, so-called “NPs libraries”, is formed in a single synthesis batch [259]. Recently the co-SoL of Au and Cu targets was studied by Chauvin et al. [174]. They showed, supported by STEM-EDS analysis of individual NPs, an increase in Au concentration in the overall NP population when increasing the power applied on the Au target. In line with the findings of Ludwig et al. [259], a large dispersion of composition was reported [174].

**Figure 21:**
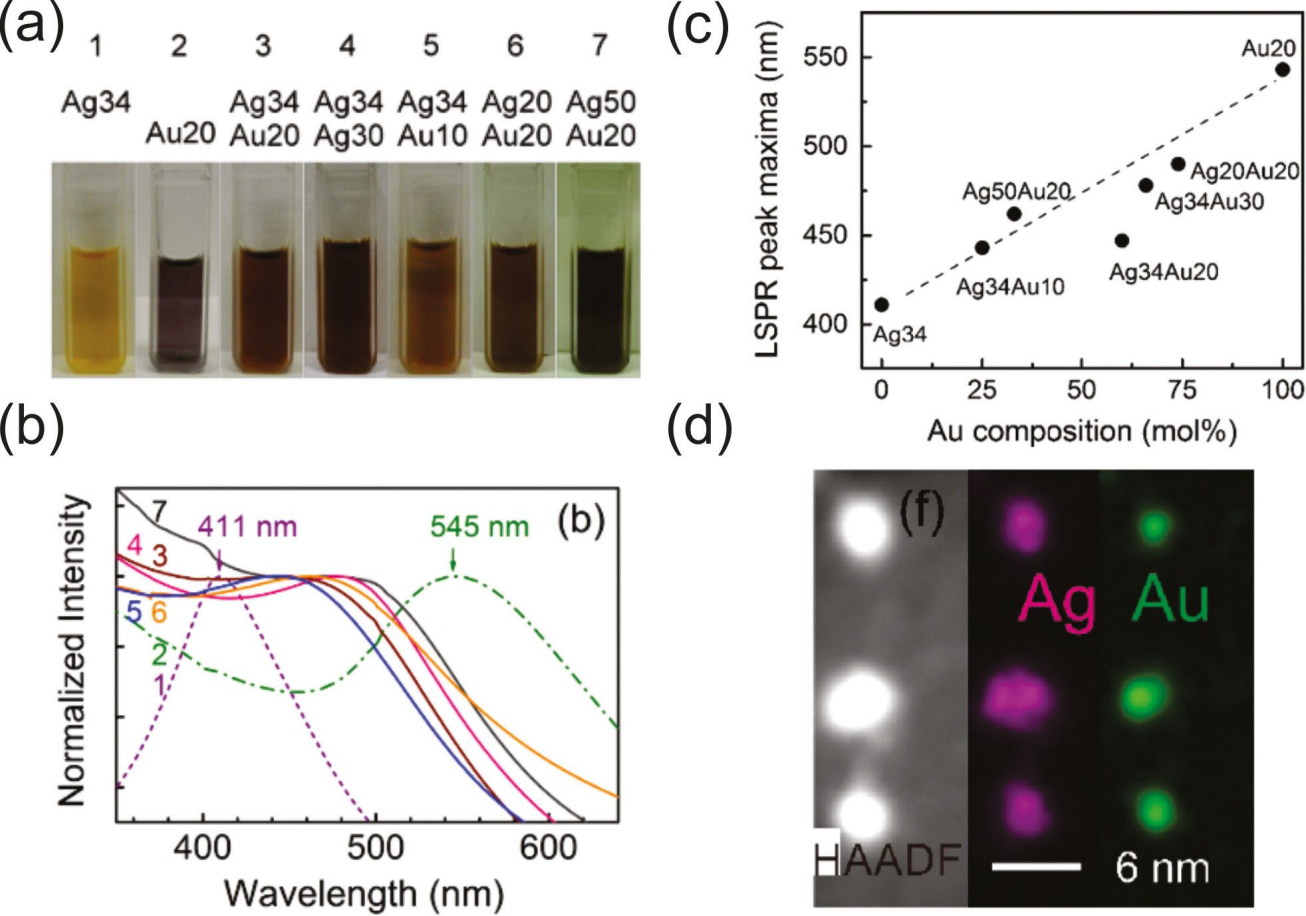
(a) Photograph of solutions containing alloy NPs obtained with different applied currents on Au and Ag target. (b) UV–vis spectra of Ag, Au, and Au/Ag alloy NPs obtained for each sample. (c) LSPR peak maxima versus Au content measured by EDS. (d) HAADF and elemental mapping of NPs from sample 4. Figure 21 was adapted with permission of Elsevier, from [224], "Double target sputtering into liquid: A new approach for preparation of Ag–Au alloy nanoparticles”, by Nguyen, M. T.; Yonezawa, T.; Wang, Y.; Tokunaga, T., in Materials Letters, 171, 75-78, Copyright (2016), permission conveyed through Copyright Clearance Center, Inc. This content is not subject to CC BY 4.0.

Cha et al. reported the production of Pt/Ni alloy NPs via co-sputtering of Pt and Ni targets [169]. The successful formation of alloy NPs has been deduced from XAFS and photoelectron spectroscopy analysis. Moreover, the composition of the NPs determined from STEM-EDS is close to the power ratio between the two targets [169]. Later, Yonezawa’s group performed co-SoL of Au and Ag [119,224]. They varied the sputtering conditions of the two targets to tune the composition of the alloy NPs over a wide range, from Ag-rich to Au-rich through equimolar Au/Ag NPs (Figure 21a). The linear relationship between the SPR peak position and the NP composition supports the effective formation of bimetallic Au/Ag alloys of varying compositions (Figure 21b,c). To further confirm their analysis, they provided STEM-EDS and elemental mappings of single NPs (Figure 21d). Then, they used the same approach to create Au/Cu alloy NPs of various controllable compositions at room temperature [174,223,260]. To the contrary of the work of Ludwig et al., the papers from Cha et al. and from the Yonezawa group report a smaller discrepancy in composition dispersion of the alloy NPs for one given condition. In the article from Yonezawa’s group [224], the deposition was made onto a stirred liquid substrate, hence mitigating the eventual spatial dispersion that could occur during co-sputter deposition. Cha et al. [169] also carried out the deposition by co-sputtering two targets, which were placed 20 cm away from the substrate holder, with the pressure set to 10 mTorr (1.3 Pa). Despite no information is provided on the geometry of the deposition setup, it can be assumed that under this condition, the product of pressure times target–substrate distance is such that both kinds of sputtered metal atoms underwent many collisions in the gas phase. Consequently, their spatial distribution is homogenized above the liquid surface, hence promoting a homogeneous chemical composition of the produced NPs.

The team of Yonezawa performed, first, the synthesis of Pd/Cu alloy NPs with a wide range of composition by varying the intensity on the targets during co-SoL [199]. However, they reported an unexpectedly high content of Pd within the alloy NPs together with a large dispersion of NPs composition under the same conditions measured by STEM-EDS as compared to XPS and XRD analysis. They suspect that Cu atoms in the alloy NPs oxidized and further dissolved, leaving the Pd/Cu alloys NPs with an excess of Pd [199]. The same year, they synthesized Pt/Au NPs with a wide range of composition from 20 to 100 atom % of Pt by co-SoL. They succeeded in synthesizing these alloy NPs even though the Pt and Au bulk metals are immiscible. The modification of the NP composition has been shown by XRD, HRTEM, and STEM-EDS by tuning the current on each target [140]. They reported a rather small discrepancy between the composition of the NPs sputtered on ILs, probed by STEM-EDS, and the composition of the thin film sputtered under the same conditions and evaluated by mass measurements. Later, a similar research has been made for Pt/Ag NPs [261]. However, the dispersion in the composition of the NPs present in the solution is large (i.e., from 16 up to 70%) [261]. Over the years, the co-SoL method has been successfully used to synthesize other bimetallic alloy NPs, such as Fe/Ni [135], or Fe/Al [200]. Moreover, the synthesis of more complex alloy NPs has been carried out, such as Cr/Mn/Fe/Co/Ni NPs, by the simultaneous sputtering of five pure metal targets. The compositional homogeneity of these quinary alloy NPs has been highlighted by STEM-EDS analysis [262].

It is important to notice here that the composition of the NPs made by co-SoL may differ from the composition of a thin film deposited under the same conditions. This discrepancy is linked to different formation pathways of thin film and NPs [260]. In the case of SoL the NP formation may take place inside the bulk solution [115,132,179,219]. Moreover, as previously said, the analysis technique used to evaluate the chemical composition is also a key parameter. ICP-AES will provide an averaged composition for the NP population present in the liquid mixture whereas spatially resolved measurements with, for example, STEM-EDS, will allow for selecting NPs one by one. In the latter case, if the sputtering process is not spatially homogeneous, such as in the case of co-sputtering (see section 1.2), discrepancies between NPs might appear.

**Figure 22:**
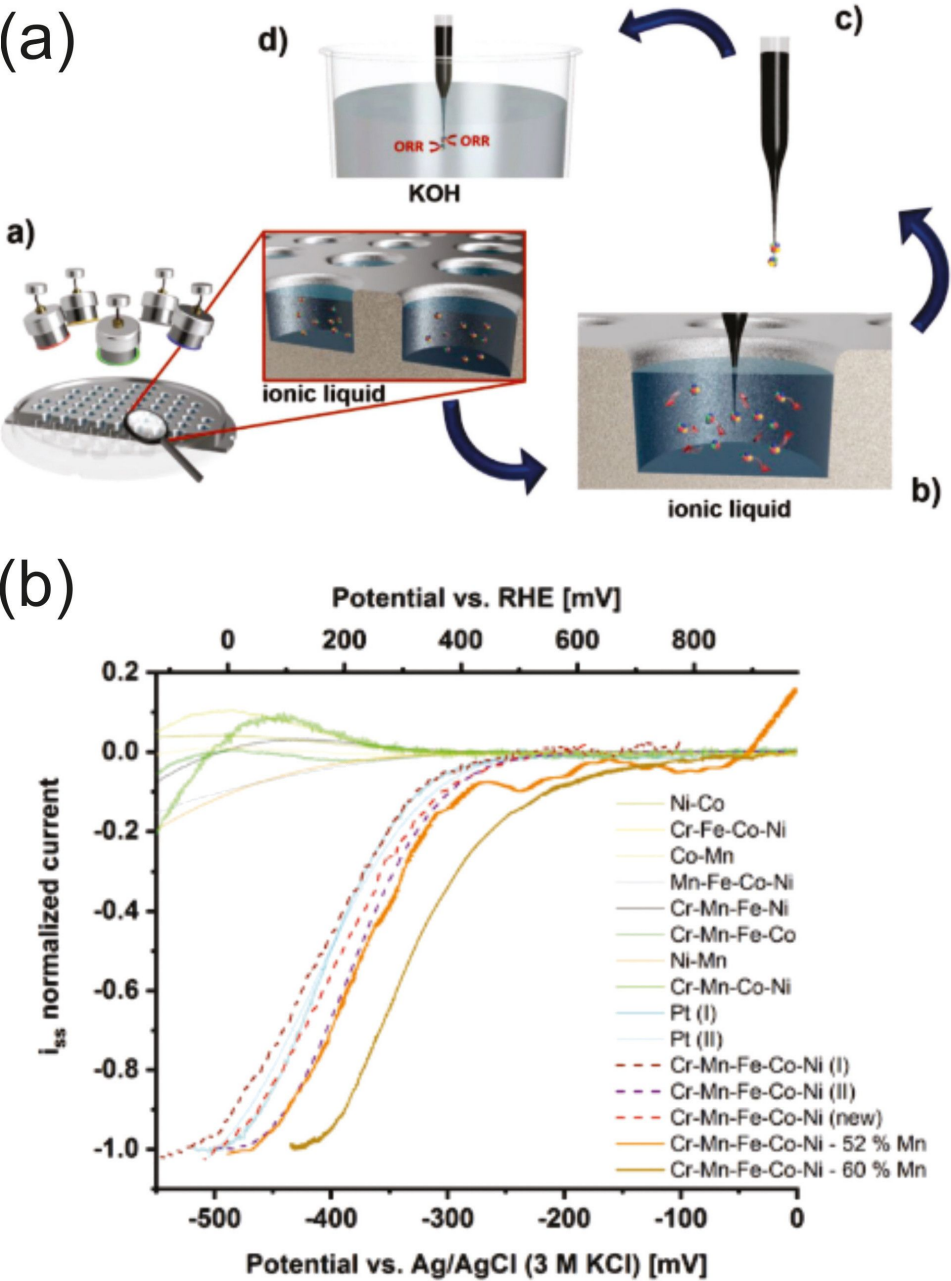
(a) Schematic of the strategy used to evaluate the intrinsic activity of alloy NPs. More details can be found in [262]. (b) Comparison of the intrinsic electrocatalytic activity of Cr/Mn/Fe/Co/Ni NPs upon the increase in Mn content. Alloy NPs close to equiatomic composition are shown with dashed lines. Curves with a Mn content of 52 and 60 atom % are shown in orange and dark yellow, respectively. All other catalytic systems are partially transparent. Figure 22 was reprinted from [262], T. Löffler et al. “Discovery of a Multinary Noble Metal–Free Oxygen Reduction Catalyst", Adv. Energy Mater., with permission from John Wiley and Sons. Copyright © 2018 WILEY-VCH Verlag GmbH & Co. KGaA, Weinheim. This content is not subject to CC BY 4.0.

**2.3.2.3 Application of multimetallic NPs:** With two or more metal elements in a single NP in the form of an alloy, multimetallic NPs obtained by SoL are promising candidates for catalysis and optical device applications [14,78]. Moreover, compared with single-metallic NPs, multimetallic NPs exhibit smaller sizes [14]. The surface area is thus higher (for an identical amount of material), exposing more active species to the surface. In this part, the properties of alloy NPs are discussed and the properties of supported alloy NPs, for instance, supported on graphene sheets, are described in the next section. Like monometallic NPs, alloy NPs have also been studied for their catalytic properties. Alloy NPs often outperform their monometallic NP counterparts. For example, Au/Cu NPs are more active than Au and Cu NPs for CO oxidation [263] and propene epoxidation [264–265]. The group of Torimoto showed that Pd/Au alloy NPs, produced by successive sputtering over ILs, exhibited higher catalytic performance regarding the oxidation of ethanol than the monometallic ones [191]. Moreover, the group of Wang revealed that trimetallic Pd/Au/Pt NPs [213], produced in the same way, yield a much higher catalytic activity for methanol oxidation than Pd/Au alloy NPs. The catalytic properties of alloy NPs have also been revealed by using non-noble metal alloy Cr/Mn/Fe/Co/Ni NPs [262]. These alloy NPs have been synthesized by co-sputtering over ILs (Figure 22a). The intrinsic electrocatalyst activity towards ORR and the relevance of the interaction between all elements have been highlighted. Moreover, the Mn content was tuned between 19 and 60 atom % to highlight the influence of the elemental composition on the electrocatalytic performance (Figure 22b) [262]. Finally, the ORR activity of these Cr/Mn/Fe/Co/Ni NPs was reported to be comparable to that of Pt [262]. This result demonstrates the usefulness of such complex NP systems for such important catalytic reactions.

**Figure 23:**
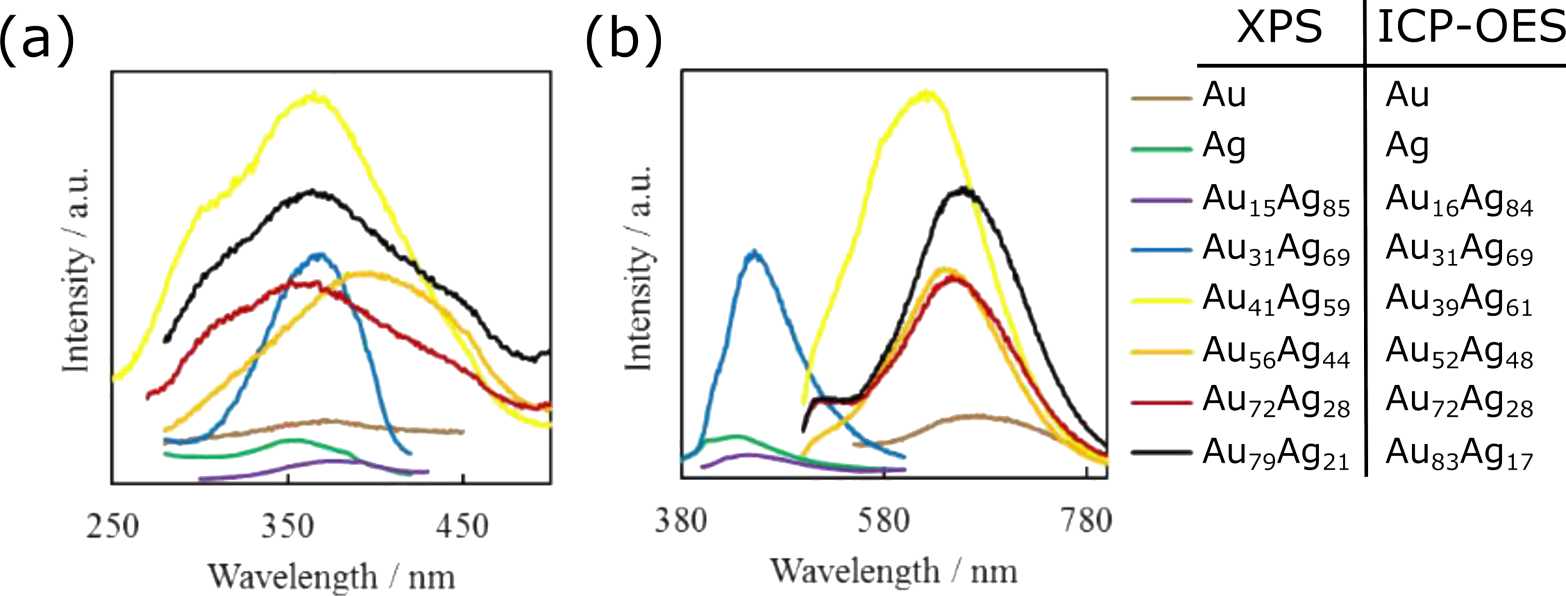
PL spectra with (a) excitation and (b) emission of Au NPs, Ag NPs, and Au/Ag alloy NPs. Figure 23 was adapted with permission from [119]. Copyright 2017 American Chemical Society. This content is not subject to CC BY 4.0.

In addition to catalytic activity, alloy NPs were found to show tunable optical properties. Many studies report a shift in the localized surface plasmon resonance (LSPR) peak position by varying the composition of the produced NPs due to the mix of both constituents at the nanoscale [113,194,200,215,224,259]. In addition to the LSPR shift, Corpuz et al. reported a novel PL emission of Au/Ag alloy NPs made by co-sputtering onto PEG containing MUTAB molecules used as a capping ligand [119]. The PL properties of the Au/Ag alloy NPs showed a broad emission tunability, from blue to NIR regions, which is correlated to their composition (Figure 23) [119].

Finally, the magnetic properties of Ni/Fe alloy NPs have been reported by Cigan et al. [135]. These NPs were produced by co-sputtering over ILs. The magnetic moment of the Ni/Fe alloy NPs was found to be similar to or even smaller than that of Ni NPs (µ_eff_ of ca. 3 µB and ca. 2 µB). Indeed, the magnetic moment is mostly driven by the strong interaction between the Ni atoms and the ILs [135].

**2.3.3 Core–shell and hollow NPs**

**Figure 24:**
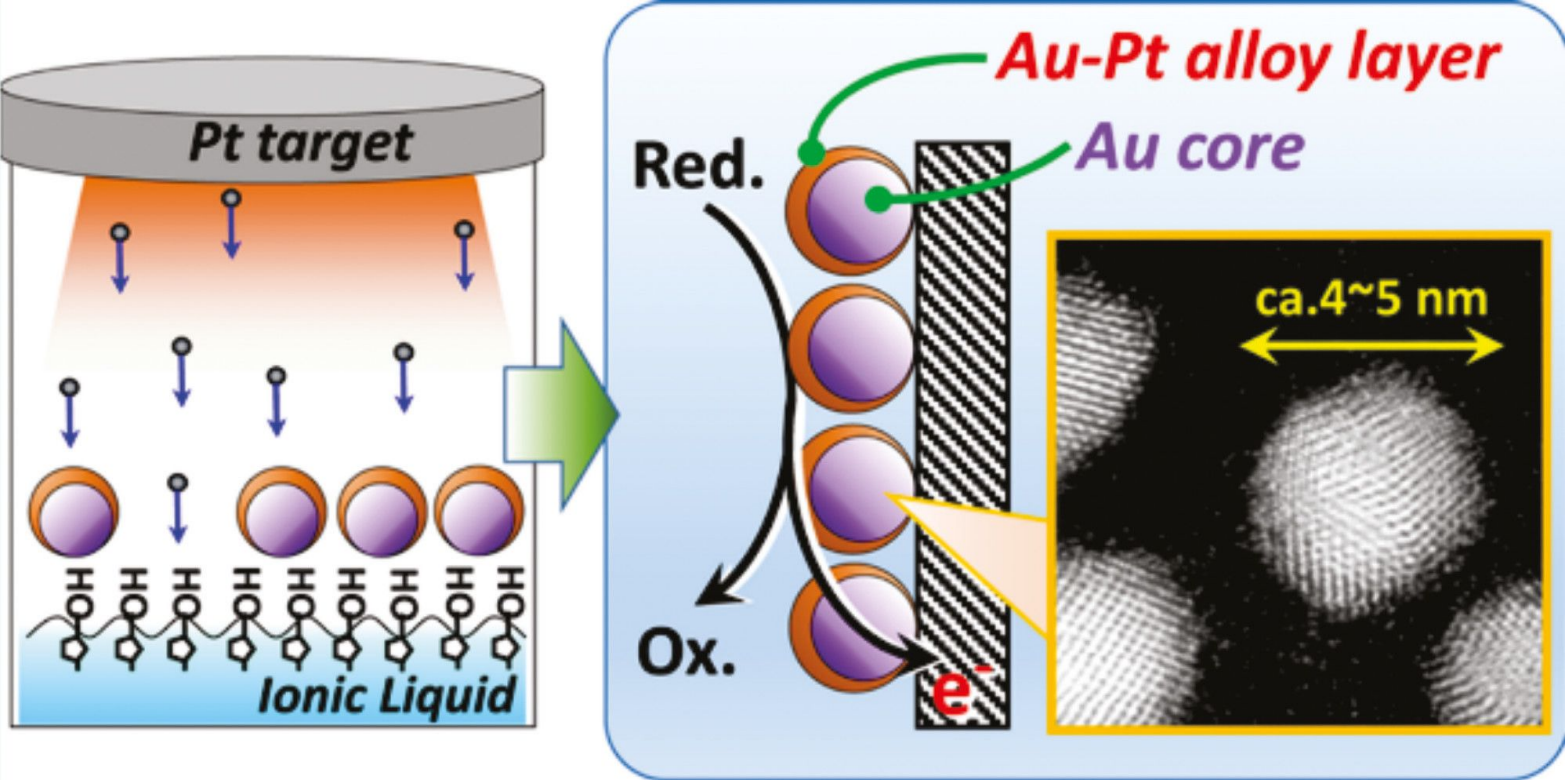
(Left) Schematic of the sequential sputtering of Pt over Au NPs stabilized on a functionalized ILs surface for the creation of Au@Pt NPs. (Right) Scheme of the electrocatalytic activity of the Au@Pt NPs monolayer and TEM image of the NPs. Figure 24 was reprinted with permission from [151]. Copyright 2016 American Chemical Society. This content is not subject to CC BY 4.0.

**2.3.3.1 Sequential sputtering with monometallic targets:** The sequential sputtering presented previously was also used to form NPs with a metal core coated with a metal (or alloy) shell [151–152,266]. The team of Wang first sputtered Ag and subsequently sputtered a second metal target such as Au and Pd. The resulting NPs were composed of an Ag core and a thin Au or Pd shell. The shell reveals various crystallographic orientations and local alloying of Ag and Au at the interface [266]. Later, Torimoto et al. used the same process to create In_2_O_3_ shells over Au, Au/Pd, and Ag NP cores. First, Au, Ag, or Au/Pd was sputtered over ILs. Then, the sputtering of In over the IL solution containing the NPs induced the creation of a thin In_2_O_3_ shell around the metallic core [152]. They also synthesized Au@Pt NPs by sputtering Pt over an Au NP monolayer floating on the surface of the liquid. This Au@Pt NPs monolayer exhibited superior electrocatalytic activity for methanol oxidation compared to pure Au or Pt NPs (Figure 24) [151]. This approach shows the high versatility of SoL for the synthesis of complex NPs, compared to chemical methods with which this type of NP structure is difficult to obtain.

**Figure 25:**
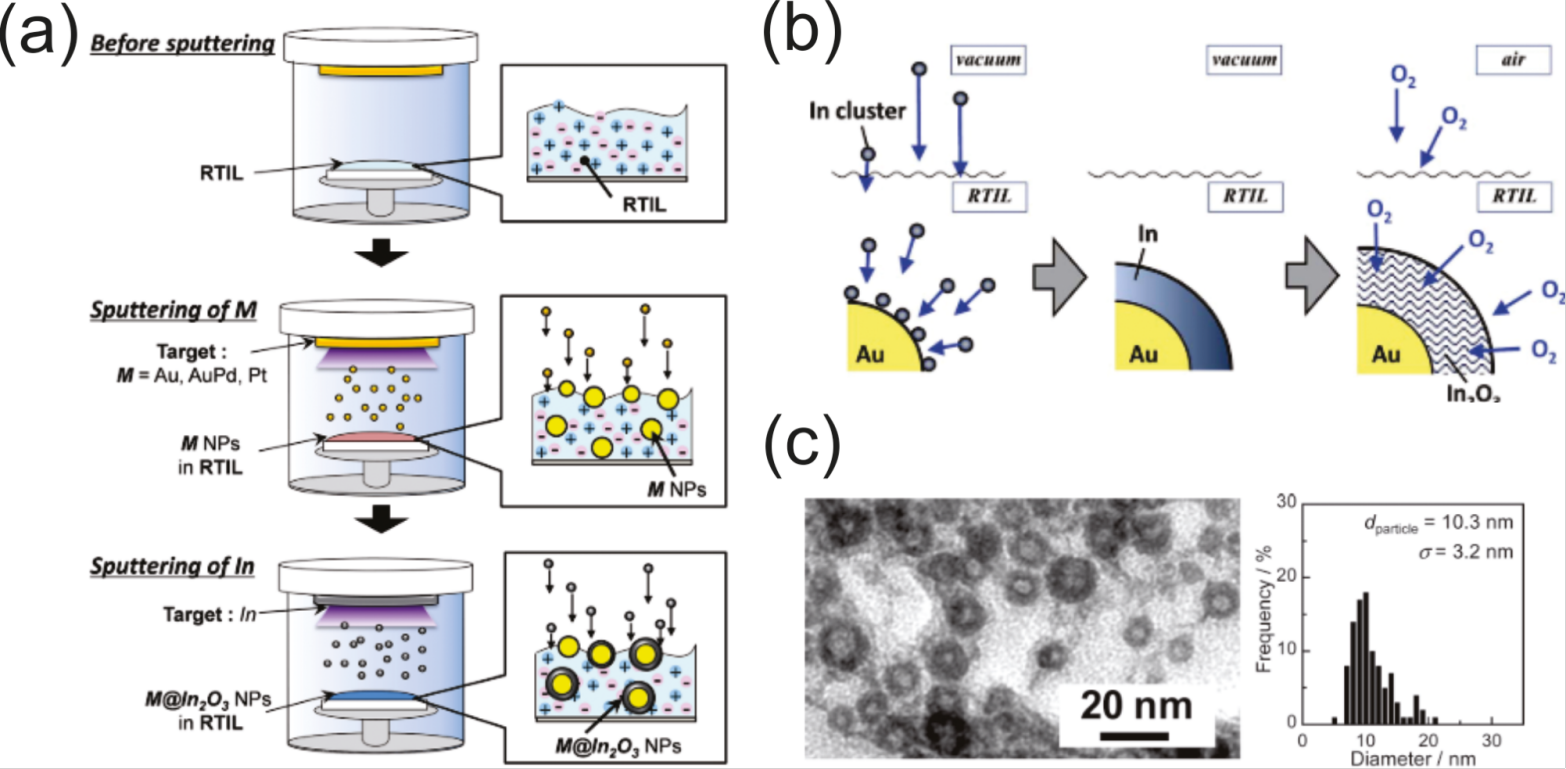
(a) Schematic of the process used for the synthesis of a noble metal (M) core with an In_2_O_3_ shell (M@In_2_O_3_ with M=Au, Au/Pd, or Pt). (b) Schematic of the formation of Au@In_2_O_3_ NPs via oxidation of In metal sputtered on a Au NP solution. Figure 25a,b was reproduced from [152] (“Ultrathin oxide shell coating of metal nanoparticles using ionic liquid/metal sputtering“, © 2015 T. Torimoto et al., published by the Royal Society of Chemistry, distributed under the terms of the Creative Commons Attribution 3.0 Unported License, https://creativecommons.org/licenses/by/3.0/). (c) Typical TEM images of In_2_O_3_ NPs prepared by heat treatment at 523 K in BMI-BF_4_ IL. Figure 25c was adapted with permission from [187]. Copyright 2010 American Chemical Society. This content is not subject to CC BY 4.0.

**2.3.3.2 Chemical reduction and Kirkendall effect:** Combining chemical reduction with SoL allows for achieving fascinating structures such as Au nanoframes [157], core@shell NPs [267], and hollow NPs [187]. The team of Torimoto performed the sputtering of an Au target onto an IL solution containing cubic Ag NPs. The selective assembly of Au NPs over the Ag nanocubes allowed forming cubic Au/Ag composites. Chemical etching of Ag from the resulting Au/Ag binary nanocomposites resulted in the formation of Au nanoframes [157]. Furthermore, Au@Pt NPs were prepared via the sputtering of Au onto ILs followed by the chemical reduction of a Pt salt on the Au NPs [267]. Finally, the use of the Kirkendall effect led to the synthesis of hollow In_2_O_3_ NPs. Briefly, the Kirkendall effect relies on the different diffusion rates of two metals upon annealing. This difference of diffusion leads to the creation of vacancies at the interface and ultimately to the creation of hollow structures [268]. An indium target was sputtered onto an IL and heat treatment was applied to the In NPs to provoke the Kirkendall effect and create hollow In_2_O_3_ NPs (Figure 25) [187]. All the approaches described here indicate that, in combination with chemical methods, SoL is a way of building up interesting nanostructures.

**2.3.4 Oxide NPs:** The SoL approach can be used for the synthesis of oxide NPs [197,201]. In 2010, the team of Torimoto sputtered W, Mo, Nb, and Ti over ILs to synthesize highly dispersed WO*_x_*, MoO*_x_*, NbO*_x_*, and TiO*_x_* NPs [197]. Later, they focused their studies on the synthesis of MoO*_x_* NPs with a controllable oxidation state by sputtering onto ILs followed by a heat treatment [201]. In other studies, the oxidation during the storage of Cu and Ti NPs has been shown. The NPs were obtained by sputtering of a Cu or a Ti target over PEEL [117,174,179]. This behavior has been highlighted by the disappearance of the SPR peak of the metal over time. TiO_2_ NPs were obtained by sputtering of Ti in argon plasma on PEEL. Most likely, oxidation of the formed Ti film deposited on the surface of the liquid occurred during venting the sample, or through the interaction of oxygen-bearing molecules in the liquid host [179].

**2.3.5 Thin films:** SoL has been used to produce thin films as well. As presented in the previous section, the formation of a film instead of NPs depends on the synthesis conditions. Indeed, in 2011, Wender et al. showed how, by tuning the sputtering parameters and the liquid substrate, one could create an Ag thin film over the liquid or a solution containing Ag NPs [124]. The morphology and properties of thin films obtained by SoL have been reported in a few studies. In 1996, Ye et al. studied the structural and electrical properties of a rough Ag thin film deposited on silicon oil by RF sputtering [10,171]. They presented for the first time that liquid surfaces can also be used as substrates just as solids. They studied the microstructure and the growth mechanism of Ag and Al films deposited under the same conditions [172]. They highlighted the difference in the resistivity evolution with the thickness between thin films deposited on a solid and a liquid substrate. Moreover, they discussed that the growth rate is sensitive to the liquid temperature, and the microstructure of the film mainly depends on the characteristics of the oil surface [170,172]. Then, they performed the deposition of a Au and a Fe thin film by thermal evaporation over silicon oil [269–272]. The structural and electrical properties of those films were similar to those in previous studies on Ag and Al films [269–271]. They also showed the coercivity behavior of the Fe film and its temperature dependence [272]. The observation of the Fe thin film morphology deposited by sputtering over silicon oil painted onto a frosted glass surface has been further investigated [130,141,273]. The appearance of disk-shaped structures has been reported [141]. Moreover, the impact of annealing on the microstructure and properties of a Fe thin film has been studied [273]. In 2007, the team of Worden also studied the reflectivity of an Ag film sputtered onto an IL for the creation of a refractive lunar telescope [226].

**2.3.6 Nanocomposite structures**

**Figure 26:**
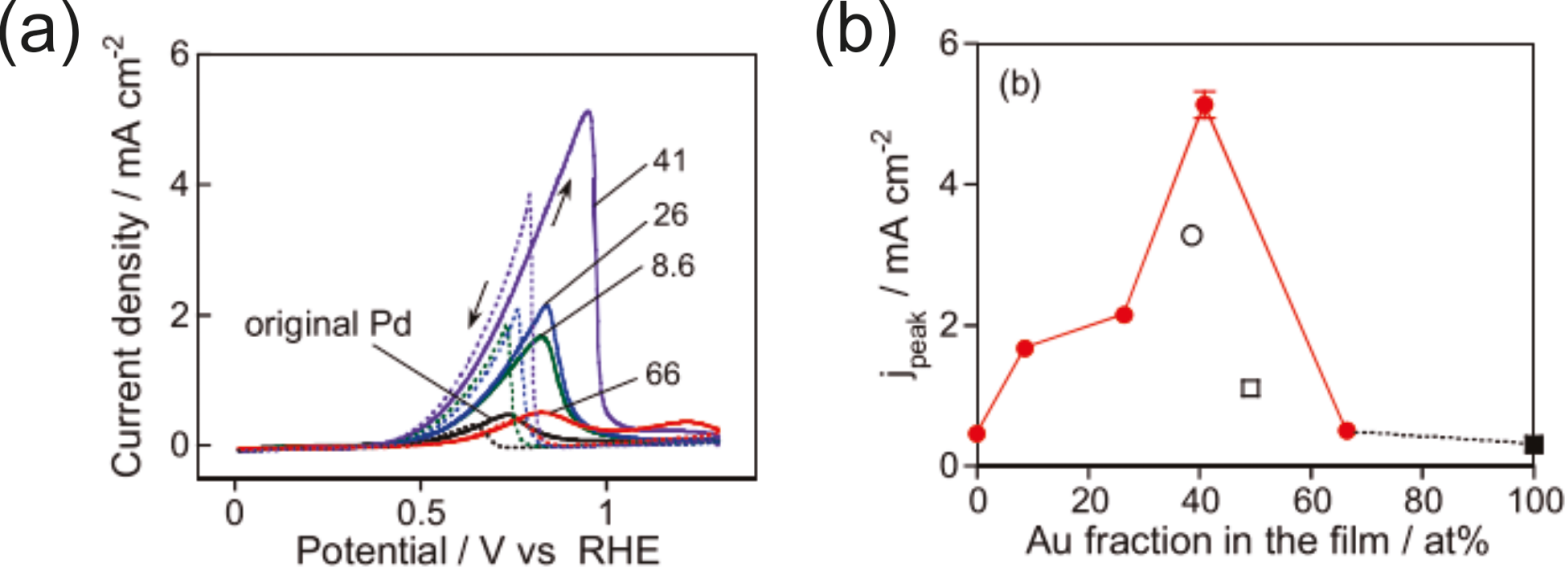
(a) Cyclic voltammograms of Pd/Au bimetallic NPs film in KOH solution. (b) Relationship between peak current density of ethanol oxidation reaction (EOR) and Au fraction of the NPs forming the films. The results are shown for Pd/Au NPs (solid circles), pure Au NPs (solid square), Au/Pd NPs prepared by the opposite sputtering sequence (open circle), and a bilayer film made of a Pd NP monolayer and a Au NP monolayer (open square). Figure 26 was adapted with permission from [191]. Copyright 2017, the Chemical Society of Japan. This content is not subject to CC BY 4.0.

**2.3.6.1 Nanoparticle stabilization onto carbon nanostructures:** The dispersion of NPs over a solid surface has been extensively studied to create carbon-based nanocomposites. Torimoto et al. reported the immobilization of sputtered Au and Au/Pd NPs on highly ordered pyrolytic graphite (HOPG). They first sputtered Au or Au/Pd targets over ILs, then spread the solution containing the NPs over HOPG, and finally performed heat treatment at different temperatures in vacuum [153]. The NP size was controlled by the temperature [153]. They also used other carbon-based surfaces for the stabilization of the NPs, such as carbon nanotubes. The synthesis of composites of Pt NPs and single-walled carbon nanotubes (SWNTs) was achieved by sputtering Pt over ILs and further adding SWNTs in the solution. The authors demonstrated the role of “glue” played by ILs in successfully synthesizing Pt NP–SWNT nanocomposites [186,243]. Later, the team of Wang showed the synthesis of different metal and alloy NPs by direct sputtering over an IL solution containing the carbon-based surfaces. They demonstrated the successful self-assembly of metal NPs (i.e., Au, Ag, and Pd), bimetallic NPs (Ag@Au and Ag@Pd), alloy NPs (Pt/Ni and Au/Pd), and trimetallic NPs (Pd/Au/Pt) on diverse carbon nanostructures [136,193,212–213,231,257,266]. This highlights that sputtering is a universal and straightforward approach to make carbon-based hybrids. Kaito et al. reported the effective stabilization Au@Pt NPs by carbon black [267]. The core@shell NPs were prepared via sputtering of Au NPs onto an IL, then carbon black was impregnated with the Au NPs before finally chemically reducing a Pt salt on the Au NPs [267]. Cha et al. demonstrated that this approach can be extended to liquid PEG substrates. They successfully synthesized metal and alloy NPs (i.e., Pt and Pt/Ni) on carbon supports [168–169]. Moreover, they proposed a mechanism underlying the chemical bonding between carbon black and Pt NPs inside ILs [168]. Overall, studies reveal the ease of separating and purifying the NPs from the liquid by introducing carbon-based nanostructures into the liquid during SoL. Moreover, the synergy of the NPs and the carbon support is very profitable for catalysis applications. Among the most commonly reported carbon surfaces to increase the catalytic activity of NPs are graphite, graphene, and carbon black. The catalytic activity of Pt NPs embedded in a glassy carbon plate was investigated [133]. In this study, the IL containing the Pt NPs was heated in the presence of a glassy carbon plate to induce the attachment. The stability and the chemical activity toward ORR of the Pt NP–glassy carbon nanocomposites were underlined [153]. Then, the catalytic properties of a Au/Pd NP–HOPG nanocomposite regarding ethanol electro-oxidation were studied. First, a sectored target of Au and Pd was sputtered onto an IL. As previously done with Pt NPs, a heating treatment was carried out to provoke the attachment between HOPG and Au/Pd NPs. The optimum activity was found for an Au fraction of 0.61 in the NPs [190]. However, due to the heating step, the NPs cannot be smaller than 7 nm, and this step can induce modifications of the NPs [190]. Accordingly, the same experiment was carried out in which the alloy NPs were synthesized by the sequential sputtering of Pd followed by Au onto the IL [191]. Then, the Au/Pd NPs stabilized on the surface of the IL were transferred by the lift-off technique on HOPG. This approach was used to avoid the heating step. The best electrocatalytic activity was obtained in this case for an Au fraction of 0.41 in the NPs (Figure 26) [191].

**Figure 27:**
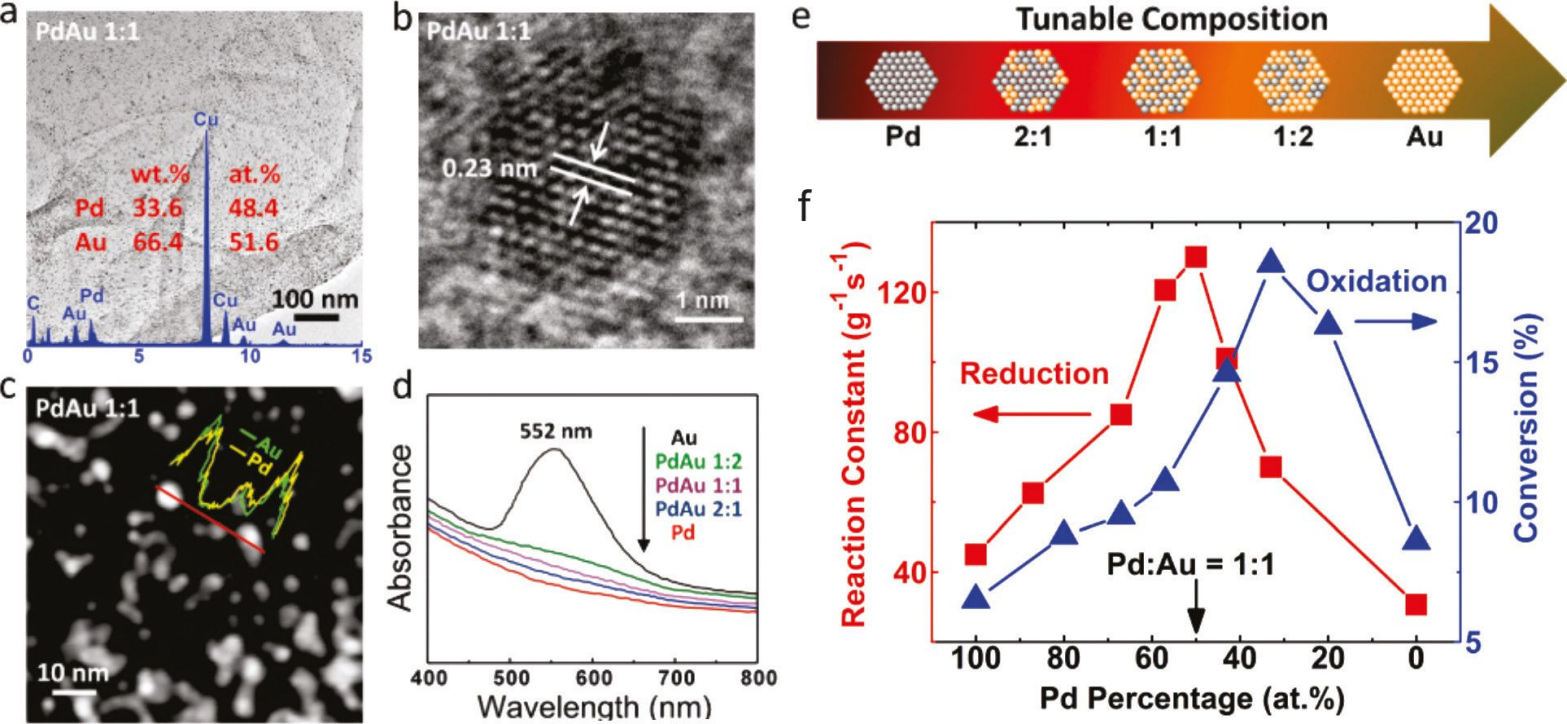
(a) TEM image with the corresponding EDS spectrum, (b) HRTEM image, and (c) HAADF-STEM image with EDS crossline profiles for graphene-supported Pd/Au alloy NPs with a Pd/Au ratio of 1:1. (d) UV–vis absorption spectra of graphene-supported Pd/Au NPs with different Pd/Au ratios. (e) Scheme showing bimetallic composition tuning of Pd/Au NPs. (f) Dependence of the catalytic activity towards cis-cyclooctene oxidation (triangle) and reduction of 4-nitrophenol (square) on the Pd fraction in graphene-supported Pd/Au alloy NPs. Figure 27 was republished with permission of The Royal Society of Chemistry, from [256] (“Controlled synthesis and synergistic effects of graphene-supported PdAu bimetallic nanoparticles with tunable catalytic properties”, by C. H. Liu et al., in Nanoscale, 7 (issue 14), 2015); permission conveyed through Copyright Clearance Center, Inc. This content is not subject to CC BY 4.0.

Au/Pd alloy NPs deposited on graphene were further studied by the group of Wang. Graphene was first dispersed in ILs and the sputtering of Au/Pd alloy targets with different compositions was performed subsequently (Figure 27a–d) [193]. They found that Au/Pd NP–graphene nanocomposites with an Au/Pd molar ratio of 1:3 exhibited superior catalytic activity and stability for ethanol electro-oxidation compared to Pd NP–graphene or Au/Pd NP–graphene nanocomposites with other compositions (Figure 27e,f) [193]. Furthermore, they revealed the high catalytic performance of these Au/Pd alloy NPs and also of Ag@Au and Ag@Pd NPs deposited on graphene for the reduction of 4-nitrophenol [256,266]. Kaito et al. pointed out the high catalytic activity of Au@Pt-NPs – carbon black nanocomposites towards ORR. Indeed, the ORR activity is approximately twice as high than that of a commercial carbon-supported Pt NP electrocatalyst [267]. The good electrocatalytic activity of carbon black-supported Pt NPs prepared by the SoL approach has been also reported by Orozco-Montes et al. [163] and Sasaki and co-workers [244].

**Figure 28:**
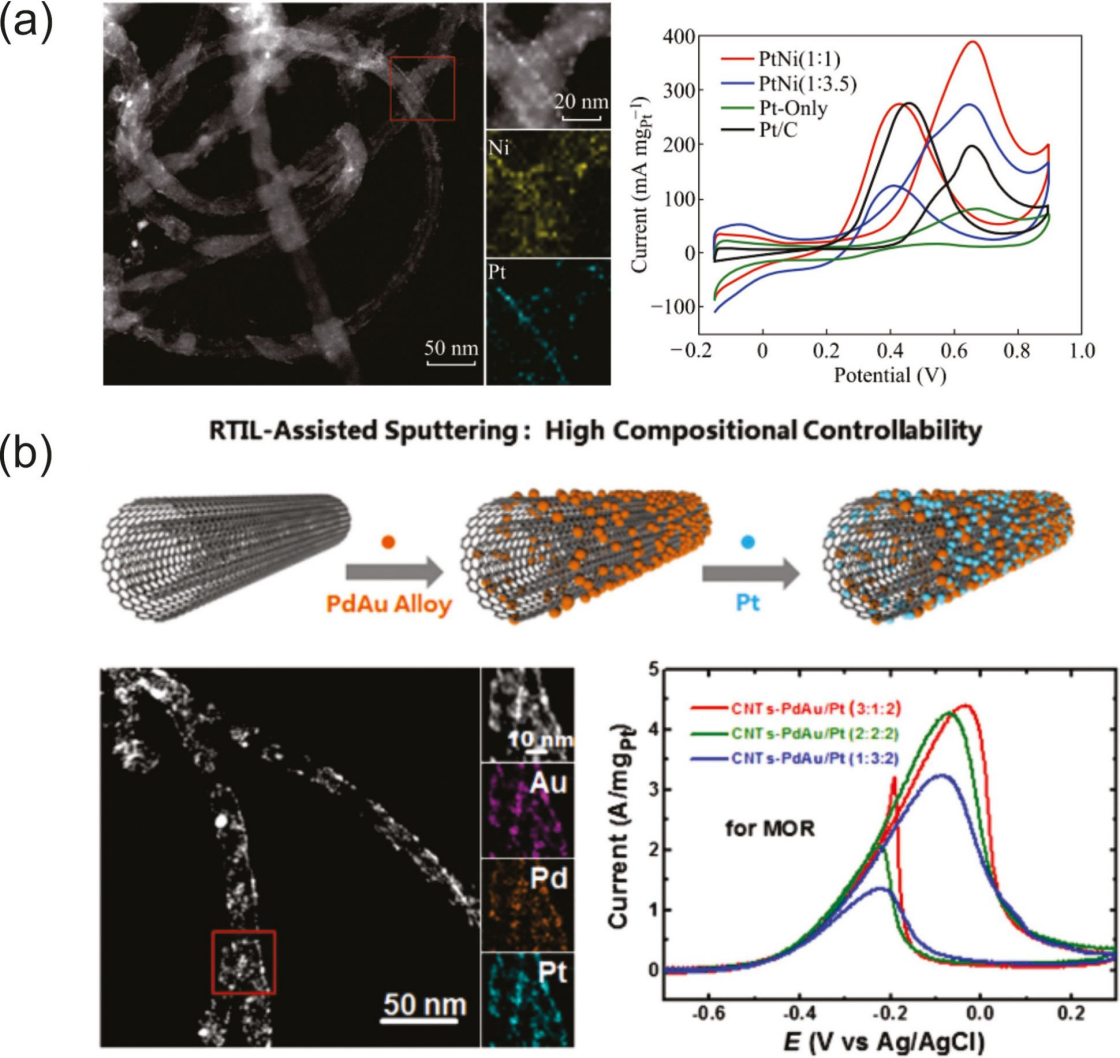
(a) Left: HAADF-STEM images of Pt/Ni NPs on MWNTs with the corresponding EDS mapping of Pt and Ni. Right: Cyclic voltammetry curves of Pt NPs and Pt/Ni NPs on MWNTs compared to a commercial Pt/C catalyst. Figure 28a was adapted from [257] (© 2016 Y. Y. Zhou et al., published by Springer Nature, distributed under the terms of the Creative Commons Attribution 4.0 International License, https://creativecommons.org/licenses/by/4.0). (b) Top: schematic of the process used to prepare Pd/Au/Pt NP–carbon nanotube composites, left: HAADF-STEM images and elemental mapping of Au, Pd, and Pt; right: cyclic voltammetry curves for the methanol oxidation reaction (MOR) using Pd/Au/Pt NP–carbon nanotube composites with different Pd/Au/Pt ratios. Figure 28b was reproduced from [213] (© 2017 X. L. Cai et al., published by Springer Nature, distributed under the terms of the Creative Commons Attribution 4.0 International License, https://creativecommons.org/licenses/by/4.0).

The performance of carbon nanotube-stabilized NPs for catalytic purposes has also been examined. First, the team of Wang studied Pt/Ni alloy NPs stabilized with multiwalled carbon nanotubes (MWNTs). The nanocomposites were obtained by sputtering a Pt/Ni alloy target over ILs containing the MWNTs. They demonstrated the high catalytic activity and long-term stability for methanol electro-oxidation (Figure 28a) [257]. Later, they revealed the higher electrocatalytic activity of Pd/Au/Pt alloy NPs stabilized with SWNTs for the methanol electro-oxidation reaction (Figure 28b) [213]. The NPs were obtained by the sequential sputtering of a Pd/Au alloy target and a Pt target [213]. The group of Torimoto also reported that Pt NP–SWNT nanocomposites provide a high and durable electrocatalytic performance for ORR [243]. Cha et al. underlined the higher activity of Pt/Ni NP–carbon black nanocomposites as compared to Pt–carbon black for ORR [169]. The alloy NPs were synthesized by co-sputtering Pt and Ni targets onto PEG containing carbon black. Moreover, they compared the catalytic properties of NPs co-sputtered onto PEG and an IL. In the case of the IL medium, no catalytic activity was detected [169].

**Figure 29:**
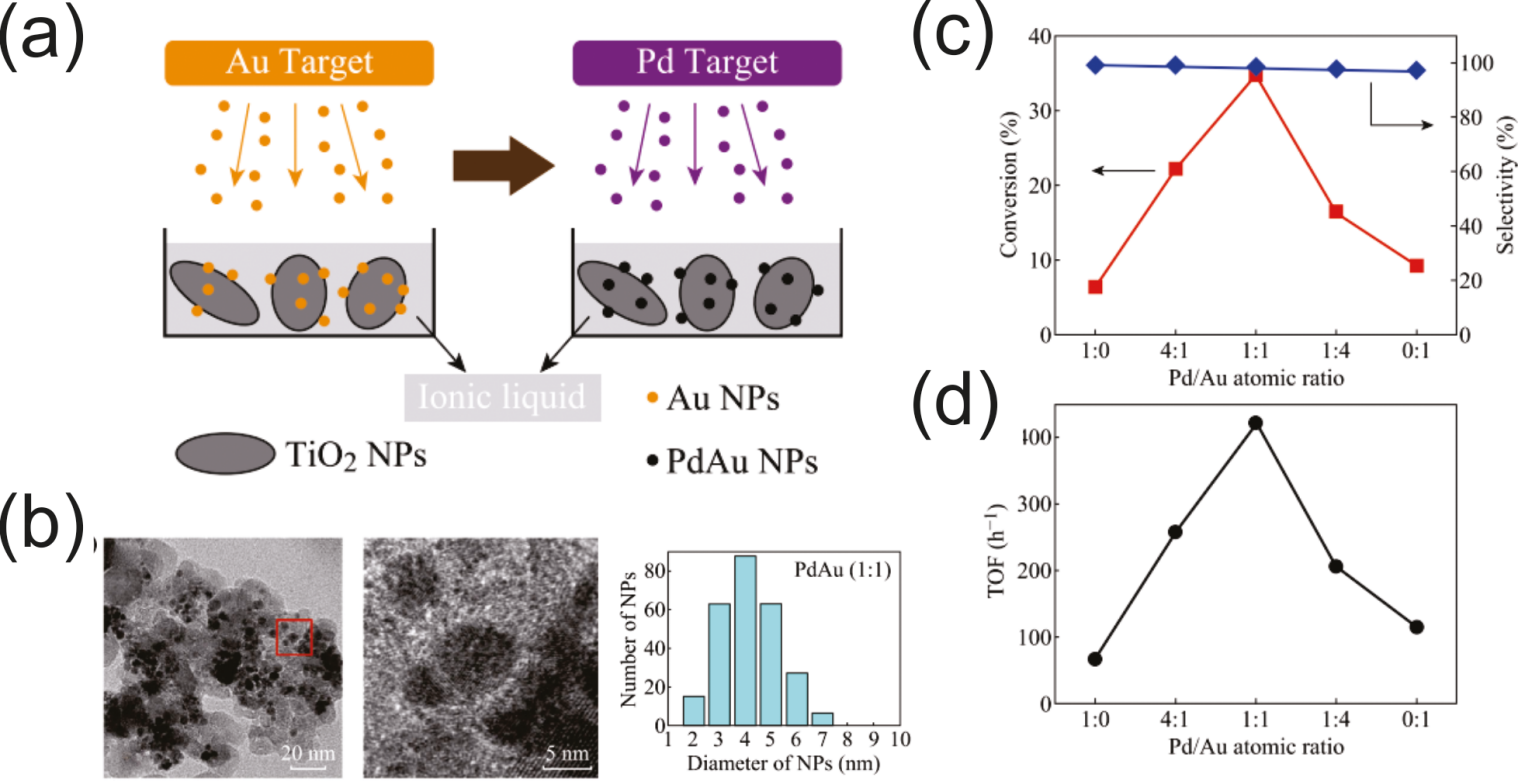
(a) Schematic of the process used for the preparation of Pd/Au NP–TiO_2_ nanocomposites by successively sputtering Au and Pd onto ILs with pre-dispersed TiO_2_ NPs. (b) TEM images and NP size distribution of Pd/Au (1:1) on TiO_2_. Dependence of catalytic activity for 1-phenylethanol oxidation as a function of the Pd/Au atomic ratio for TiO_2_-supported alloy NPs; (c) (square) conversion and (diamond) selectivity; (d) turnover frequency. Figure 29 was adapted from [192] (© 2015 J. B. Chang et al., published by Springer Nature, distributed under the terms of the Creative Commons Attribution 4.0 International License, https://creativecommons.org/licenses/by/4.0).

**2.3.6.2 Nanoparticle stabilization onto a metal or metal-oxide structures:** NPs are mainly stabilized on solid carbon surfaces for the improvement of their catalytic activity. However, a few studies also describe the stabilization of NPs on oxide or metallic nanostructures. In most cases, the stabilization is used to simplify the separation and purification of the NPs but also to increase the catalytic activity. Richter et al. first reported the coating of ZnO structures by Cu NPs. They dispersed ZnO in ILs before sputtering copper to stabilize the Cu NPs [230]. The team of Torimoto selectively assembled Au NPs, obtained by sputtering, over the edges and vertices of Ag nanocubes. They further dissolved the Ag nanocubes by chemical etching to create Au/Ag nanocubes and Au nanoframes [157]. The team of Torimoto also showed the adsorption of Au NPs on a TiO_2_(110) substrate with the IL encapsulating the NPs on the TiO_2_ surface [180]. Through a post-treatment, they immobilized the Au NPs and removed the IL layers, resulting in self-assembled Au NP arrays on the TiO_2_(110) substrate [180]. Later, the team of Wang reported the successive sputtering of an Au and a Pd target onto ILs containing TiO_2_ NPs. They reported the high catalytic activity of the Pd/Au alloy NPs deposited on TiO_2_ (Figure 29) [192].

**Figure 30:**
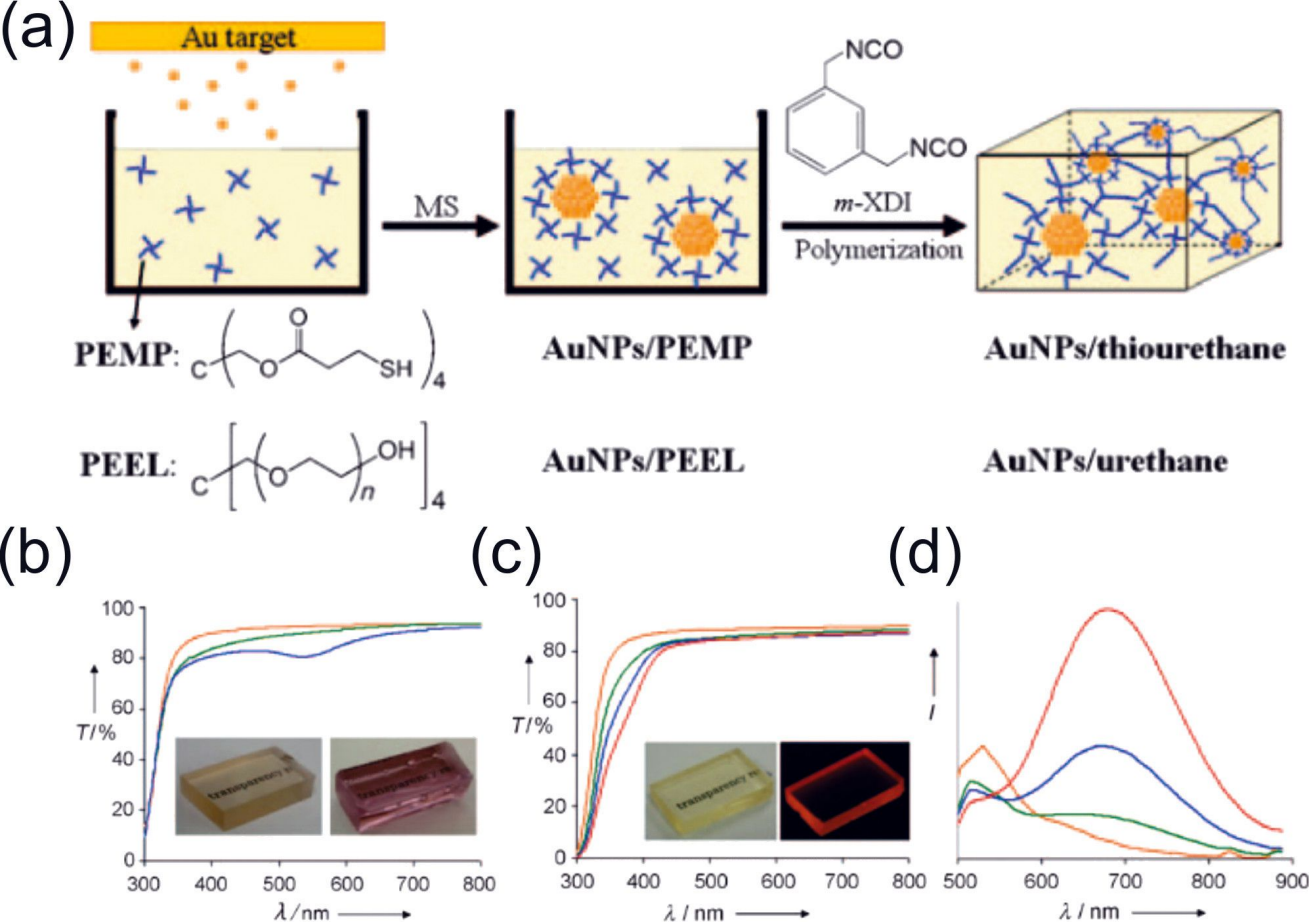
(a) Schematic of the process used for the preparation of resins containing Au NPs. (b) UV–vis transmittance spectra of (orange) urethane, (green) fresh Au NP–urethane resin, and (blue) matured AuNP–urethane resin; inset: photographs of fresh (left) and matured (right) AuNP–urethane resin. (c) Transmittance and (d) PL of AuNPs in thiourethane resin for the different sputtering times of the Au target: 0, 5, 10, and 15 min for orange, green, blue, and red curves, respectively. Inset: photograph of the AuNP–thiourethane resin made by 15 min of sputtering under daylight (left) and 365 nm UV irradiation (right). Figure 30 was adapted from [143], Y. Shishino et al. “Preparation of Optical Resins Containing Dispersed Gold Nanoparticles by the Matrix Sputtering Method", Angewandte Chemie International Edition, with permission from John Wiley and Sons. Copyright © 2011 WILEY-VCH Verlag GmbH & Co. KGaA, Weinheim. This content is not subject to CC BY 4.0.

**2.3.7 Nanoparticle stabilization in monomer solutions or other matrices:** The sputtering onto cross-linkable monomers is a robust and straightforward way to embed NPs in a solid polymer matrix. This method is advantageous over other techniques used for the dispersion of NPs in a polymer matrix since it allows for easier, faster, and purer synthesis, because the method is a physical process that utilizes only pristine materials of the constituents [147]. Ozaki and coworkers developed this method to fabricate metal NPs dispersed inside liquid crystal (LC) suspensions [147]. Using LC molecules that possess a vapor pressure smaller than 1 Pa at room temperature is sufficient to carry out the sputtering deposition process. The extinction spectrum of matrix and NPs revealed that the coloring of the LC is attributed to the local surface plasmon resonance of Au [147]. Then, the team of Yonezawa presented the sputtering of Au and Ag over PEEL and PEMP, followed by their polymerization into a thiourethane and urethane resin (Figure 30a) [123,143,196]. The obtained Au NP–resin composite exhibits luminescence at 690 nm. The authors also emphasized that, by tuning the interaction between Au NPs and the matrix together with the size and shape of the Au NPs, one can adjust the surface plasmon absorption of the Au NP–resin while keeping a good transparency (Figure 30b,c) [143]. Moreover, they reported the fluorescence properties of Ag NPs embedded in a thiourethane matrix. They discussed the dependence of the extinction spectra and fluorescence spectra as a function of the aggregation of the NPs (Figure 30d) [123,196].

## Conclusion

Sputtering onto liquids, SoL, is a unique synthetic approach that bridges the fields of physical vapor deposition and colloidal chemistry. From this review article, the following main advantages of the SoL technique can be highlighted: (i) SoL is an automatized approach that provides a very good reproducibility of the synthetic procedures. (ii) Colloidal solutions of NPs produced by SoL have a high purity since they contain only two components (target material and host liquid). (iii) SoL allows for producing small clusters with a diameter of less than 1 nm. (iv) SoL is a straightforward approach to make carbon-supported hybrid nanomaterials. (v) The (magnetron) sputtering technique allows for the deposition of a very wide range of existing solid materials. Theoretically, the SoL approach should be applicable for the preparation of colloidal solutions of NPs that are difficult to obtain by classical colloidal synthesis or by top-down techniques.

It is worth mentioning that the properties of products obtained by SoL depend on the physicochemical interactions between host liquid, plasma, and sputtered material. Changing the working parameters as well as changing the sputter device will lead to different plasma characteristics and particle fluxes towards the liquid surface, which will ultimately affect the properties of the final products. We would like to encourage authors working with the SoL approach to provide a full set of experimental parameters, including detailed information about their sputter devices and deposition rates. This will allow for comparing the fluxes of sputtered material and other plasma species and will facilitate reproducing the published synthetic protocols in different apparatuses, such as compact sputter coaters and full-size PVD chambers.

Of course, a series of systematic in situ studies of the SoL process is required to fully understand the relationship between the experimental parameters and the mechanism of NP formation. We believe that implementing in situ SAXS and/or UV–vis measurements during sputtering onto various host liquids will enable the detailed analysis of nucleation and growth steps, such as it was done for classical colloidal synthesis during the last few decades.

Tables containing the most important sputtering parameters (target material and target diameter, working distance, working gas composition, working gas pressure, current, voltage, and sputter time), host liquid parameters (liquid composition and volume or weight, vessel size, and temperature) and the size of obtained monometallic or oxide NPs for 89 references published on SoL can be found in the Supporting Information.

## Supporting Information

Tables containing the most important sputtering parameters (target material and target diameter, working distance, working gas composition, working gas pressure, current, voltage, and sputter time), host liquid parameters (liquid composition and volume or weight, vessel size, and temperature) and the size of obtained monometallic or oxide NPs for 89 references published on SoL can be found in the Supporting Information.

File 1 Sputtering onto liquids: Table with experimental parameters.

File 2 Comparison the sizes of the metal NPs prepared by magnetron sputtering onto similar host liquids.
