# Supplementary material for: Sputtering onto liquids: a critical review
Source: Beilstein J Nanotechnol. 2022 Jan 4;13:10–53. doi: 10.3762/bjnano.13.2 (PMC8744456; doi:10.3762/bjnano.13.2)
Supplement: File 1 — Sputtering onto liquids: Table with experimental parameters. [file Beilstein_J_Nanotechnol-13-10-s001.pdf]

BEILSTEIN JOURNAL OF NANOTECHNOLOGY

Supporting Information

for

Sputtering onto liquids: a critical review

Anastasiya Sergievskaya, Adrien Chauvin and Stephanos Konstantinidis

Beilstein J. Nanotechnol. **2022,** *13,* 10–53. doi:10.3762/bjnano.13.2

Sputtering onto liquids: Table with experimental parameters

License and Terms: This is a supporting information file under the terms of the Creative Commons Attribution License (https://creativecommons.org/licenses/by/4.0). Please note that the reuse, redistribution and reproduction in particular requires that the author(s) and source are credited and that individual graphics may be subject to special legal provisions.
The license is subject to the *Beilstein Journal of Nanotechnology* terms and conditions: (https://www.beilstein-journals.org/bjnano/terms)

Experimental parameters

Target: target material

Liquid: host liquid

Amount of liquid substrate: volume or weight of the host liquid plus parameters of the vessels

d_T,cm: diameter of the target, centimeter

WD, cm: working distance, centimeter

***p*, Pa:** pressure, Pascal

***P*, W:** power, Watt

***V*, V:** voltage, volt

***I*, mA:** current, milliampere

t_s, min: sputter time, minute

***T*s, °C:** temperature, degree Celsius

d_{NP}, nm: diameter of nanoparticles according to TEM, nanometer

Ref.: reference

Acronyms

EMIM – 1-ethyl-3-methylimidazolium

TMPA – N,N,N-trimethyl-n-propylammonium

BMIM – 1-butyl-3-methylimidazolium

HyEMIM – 1-(2-hydroxyethyl)-3-methylimidazolium

(BCN)MIM – 1-butyronitril-methylimidazolium

(MeOE)MIM – 1-methoxyethyl-methylimidazolium

(HSE)MIM – 1-thioethyl-methylimidazolium

PP13 – 1-methyl-1-propylpiperidinium

TFSI – bis(trifluoromethanesulfonyl)imide (=NTf_2)

OTf – trifluoromethanesulfonate

FSA – bis(fluorosulfonyl)amide

OTD – 1-octadecane-thiol

PEMP – pentaerythritol tetrakis(3-mercaptopropionate)

PVP – polyvinylpyrrolidone

PEEL – pentaerythritol ethoxylate

PEG – polyethylene glycol

SMP – 3-mercaptopropionate

MUA – 11-mercaptoundecanoic acid

DES – deep eutectic solvents

Table S1: Table with experimental parameters.

					IONIC LIQUID: EMIM – BF_4							
**Target**	**Liquid**	**Amount of liquid substrate**	***d_T*, *cm***	**WD, cm**	***p*, Pa**	***P*, W**	***V*, V**	***I*, mA**	***t_s*, min**	***T_s*, C**	***d_{NP}*, nm**	**Ref.**
Au	EMIM – BF_4	0.60 cm^3 on 20 cm^2 glass plate	5	3.5	20 (air)	-	-	4.0	30	RT	5.5±0.86	[1]
Au	EMIM – BF_4	80 mm^3 on 4 cm^2 glass plate	5	3.5	20 (Ar)	-	-	10	5	RT	2.9±0.8	[2]
Au	EMIM – BF_4	0.60 cm^3 on 10 cm^2 glass plate	4.9	2.5	2 (Ar)	-	-	10	10	RT	2.2	[3]
W<br>Mo<br>Nb<br>Ti	EMIM – BF_4	0.60 cm3 on 10 cm^2 glass slide	4.9	2	2 (Ar)	-	-	40	10	RT	WO_x -3.2 -4.8<br>MoO_x - 2.7 - 3.7<br>NbO_x - 1.9 - 2.9<br>TiO_x - 3.4 - 4.6	[4]
In	EMIM – BF_4	0.60 cm3 on 10 cm^2 glass slide	4.9	2	2 (Ar)	-	-	10	10	RT	In@In_2O_3: 8±2	[5]
					IONIC LIQUID: TMPA – TFSI							
**Target**	**Liquid**	**Amount of liquid substrate**	***d_t*, *cm***	**WD, cm**	***p*, Pa**	***P*, W**	***V*, V**	***I*, mA**	***t_s*, min**	***T_s*, C**	***d_{NP}*, nm**	**Ref.**
Au	TMPA – TFSI	0.6 cm^3 on 20 cm^2 glass plate	5	3.5	20 (air)	-	-	4.0	30	RT	1.9±0.46	[1]
Au	TMPA – TFSA	0.8 cm^3 on 4 cm^2 glass plate	5	3.5	20 (Ar)	-	-	10	5	RT	2.2±0.4	[2]
Au	TMPA – TFSI	0.6 cm^3 on 10 cm^2 glass plate	5	3.5	20 (Ar)	-	-	40	5	RT	2.3±0.3	[6]
Au<br>Pt	TMPA –TFSI	? cm^3 on a glass plate 10 cm^2	5	2	10 (Ar)	-	500	40	5	RT	Au: 2.9 ± 0.7<br>Pt: 1.0 ± 0.3	[7]
Pt	TMPA – TFSI	0.4 cm^3 on a glass plate (2.5 x 2.5 cm)	5.7	4.5	7 (Ar)<br>7 (N_2)	-	-	40	5	RT	(Ar): 2.24±0.36<br>(N_2): 3.28±0.60	[8]
Pt	TMPA – TFSI	0.2 cm^3 on a glass plate (2.5 x 2.5 cm)	5.7	4.5	7 (Ar)	-	-	40	5; 15; 30	RT	2.28; 2.37; 2.27	[9]
					IONIC LIQUIDS: BMIM – BF_4							
**Target**	**Liquid**	**Amount of liquid substrate**	***d_t*, *cm***	**WD, cm**	***p*, Pa**	***P*, W**	***V*, V**	***I*, mA**	***t_s*, min**	***T_s*, C**	***d_{NP}*, nm**	**Ref.**
Au	BMIM – BF_4	0.8 cm^3 on a glass plate 4 cm^2	5	3.5	20 (Ar)	-	-	10	5	RT	2.5±0.6	[2]
Au	BMIM – BF_4	0.6 cm^3 on a glass plate (26mm x 38mm)	5	3.0	20 (Ar)	-	-	40	5	RT	2.6 ± 0.4	[10]
Au	BMIM – BF_4	2.0 cm^3 on a stainless plate 15.9 cm^2	5	2.5	12-13 (Ar)	-	1000	20	50	20 → 80	(SAXS): 0.6 → 3.5	[11]
Au	BMIM – BF_4	2.0 cm^3 on a stainless plate 15.9 cm^2	5	2.5; 5.0; 7.5	13-30 (Ar)		700→1000	20 → 40	50	20	From 1 to 3	[12]
Au	BMIM – BF_4	1.23 g in a cylindrical glass support (3 cm)	5	5	2.1 (Ar)	-	335	40	2.5	RT	3.6 ± 0.4	[13]
In	BMIM – BF_4	0.60 cm3 on 10 cm^2 glass slide	4.9	2	2 (Ar)	-	-	10	10	RT	In@In_2O_3: 9.7±2.0	[5]

Table S1: Table with experimental parameters.

Pt	BMIM – BF_4	0.4 cm^3 on 6.3 cm^2 glass plate		2.5	9.5 (Ar)	-	-	40	15	RT	1.2	[3]
IONIC LIQUID: BMIM – PF_6												
**Target**	**Liquid**	**Amount of liquid substrate**	***d_t*, *cm***	**WD, cm**	***p*, Pa**	***P*, W**	***V*, V**	***I*, mA**	***t_s*, min**	***T_s*, C**	***d_{NP}*, nm**	**Ref.**
Au	BMIM – PF_6	1.23 g in a cylindrical glass support (3 cm)	5	5	2.1 (Ar)	-	335	40	2.5	RT	3.7 ± 0.4	[13]
Au	BMIM – PF_6	? cm^3 on 10 cm^2glass plate	5	3.5	20 (Ar)	-	-	40	5	RT	2.6 ± 0.3	[14]
Au	BMIM – PF_6	0.6 cm^3 on a glass plate (26mm x 38mm)	5	3.0	20 (Ar)	-	-	40	5	RT	2.3 ± 0.4	[10]
Au	BMIM – PF_6	0.60 cm^3 on 5.7 cm^2 glass slide	5	3.5	20 (air)	-	-	40	5	RT	2.5	[15]
Au	BMIM – PF_6	0.60 cm^3 on 10 cm^2 glass slide	5	3.5	20 (Ar)	-	-	40	5	RT	2.6±0.6	[16]
Au	BMIM – PF_6	1.0 cm^3 on 6.1 cm^2 Teflon plate	5	3.5	20 (Ar)	-	-	40	5	RT	Au: 2.6±0.3	[17]
Au	BMIM – PF_6	2.0 cm^3 on 15.9 cm^2 stainless plate	5	2.5	12-14 (Ar)	-	1000	20	50	20 → 80	(SAXS): 0.5 → 3	[18]
Au	BMIM – PF_6	2.0 cm^3 on 15.9 cm^2 stainless plate	5	2.5	16-19 (Ar)	-	1000	20	50	RT	(SAXS): 1.0–1.5	[19]
Ag	BMIM – PF_6	0.6 cm^3 on 10 cm^2 glass plate	5	8.5<br>8.5	5 (Ar)<br>5 (Ar)	-	-	10<br>40	5→45<br>5	RT<br>RT	5.7±1.8<br>11	[20]
Ag	BMIM – PF_6	1.0 cm^3 on 6.1 cm^2 Teflon plate	5	3.5	20 (Ar)	-	-	40	5	RT	Ag: 6.0±1.5	[17]
Ag	BMIM – PF_6	multiple cavity holder (40 µL per cavity)	3.81		0.5 (Ar)	30	-	-	15	RT	6±3	[21]
Pd	BMIM – PF_6	0.2 cm^3 on 2.5 x 2.5 cm glass plate	5.7	4.5	8 (Ar)	-	-	40	5	RT	3.0	[22]
IONIC LIQUID: BMIM – TFSI												
**Target**	**Liquid**	**Amount of liquid substrate**	***d_t*, *cm***	**WD, cm**	***p*, Pa**	***P*, W**	***V*, V**	***I*, mA**	***t_s*, min**	***T_s*, C**	***d_{NP}*, nm**	**Ref.**
Au	BMIM – TFSI	0.6 cm^3 on a glass plate (26mm x 38mm)	5	3	20 (Ar)	-	-	40	5	RT	2.0 ± 0.4	[10]
Au	BMIM – TFSI	2.0 cm^3 on 15.9 cm^2 stainless plate	5	2.5	12-14 (Ar)	-	1000	20	50	20 → 80	(SAXS): 0.5 → 3	[18]
Au	BMIM – TFSI	1.23 g in a cylindrical glass support (ϕ = 3cm)	5	5	2.1 (Ar)	-	299<br>322<br>335<br>358<br>410<br>335<br>335<br>335	20<br>30<br>40<br>60<br>110<br>40<br>40<br>40	2.5<br>2.5<br>2.5<br>2.5<br>2.5<br>5.0<br>7.5<br>10	RT<br>RT<br>RT<br>RT<br>RT<br>RT<br>RT<br>RT	3.2 ± 0.5<br>3.4 ± 0.5<br>3.5 ± 0.6<br>3.9 ± 0.8<br>4.6 ± 0.7<br>4.0 ± 0.9<br>3.9 ± 0.8<br>4.0 ± 0.8	[13]
Au	BMIM – TFSI	0.6 cm^3 on 10 cm^2 glass plate	5	3.5	20 (Ar)	-	-	40	5	RT	2.3±0.3	[6]

Table S1: Table with experimental parameters.

Au	BMIM – TFSI	multiple cavity holder (40 µL per cavity)	3.81		0.5 (Ar)	30	-	-	30	RT	Au: 2.1 ± 0.7	[23]
Cu	BMIM – TFSI	multiple cavity holder (30 µL per cavity)	3.81		0.5 (Ar)	30	-	-	120	RT	Cu: 2.8 ± 1.1	[23]
						IONIC LIQUID: BMIM – TFSI						
Cu	BMIM - TFSI	50 ml of IL in a petri dish (110 mm inner diameter × 20 mm height) with 30 rpm	3.81	9	0.5 (Ar)	30	405	75	24 hours	RT	2.6±1	[24]
Pd	BMIM – TFSI	0.2 cm^3 on 2.5 x 2.5 cm glass plate	5.7	4.5	8 (Ar)	-	-	40	5	RT	2.2	[22]
Ag	BMIM – TFSI	4.0 cm^3 into a cylindrical ceramic crucible (ϕ =4.5 cm)	7.5	15	1.3 (Ar)	20	-	-		RT	5-20	[25]
Ag	BMIM – TFSI	multiple cavity holder (40 µL per cavity)	3.81		0.5 (Ar)	30	-	-	15	RT	8±4	[21]
						OTHER IONIC LIQUIDS						
**Target**	**Liquid**	**Amount of liquid substrate**	***d_t*, *cm***	**WD, cm**	***p*, Pa**	***P*, W**	***V*, V**	***I*, mA**	***t_s*, min**	***T_s*, C**	***d_{NP}*, nm**	**Ref.**
Au	HyEMIM – BF_4<br>OMIM – BF_4<br>HyEMIM – TFSA<br>EMIM – TFSA<br>Ch – TFSA<br>SBMI – TFSA<br>EMIM – EtSO4<br>EMIM – Ac<br>BMIM – SCN<br>BMIM – actate<br>HyEA – formate	0.8 cm^3 on a glass plate 4 cm^2	5	3.5	20 (Ar)	-	-	10	5	RT	5.1±0.7<br>2.4±0.8<br>4.3±0.8<br>2.9±0.6<br>5.5 ±0.7<br>2.2±0.6<br>2.5±0.6<br>3.8±1.3<br>2.8±0.7<br>3.0±0.7<br>5.2±1.3	[2]
Au	HyEMIM – BF_4	0.80 cm^3 on 4.0 cm^2 glass plate	5	3.5	20 (Ar)	-	-	10	2.5	RT	4.2	[26]
Au	(BCN)MIM – TFSI	1.2 g on a Petri plate (ϕ =3 cm)	5	5	2 (Ar)	2→45	275 → 410	-	2.5	RT	8.7→5.1	[27]
Au	[PY1,1O1] – BF_4<br>[N122,1O2] – BF_4	0.6 cm^3 on a glass plate (26mm x 38mm)	5	3	20 (Ar)	-	-	40	5	RT	2.7 ± 0.5<br>3.5 ± 0.7	[10]
Au	AMIM – TFSI<br>AMIM – BF_4	Layer of IL on Si(111)	-	-	3 (Ar)	50	-	-	5 sec	RT	5.1 ± 0.8<br>6.5 ± 0.7	[28]
Au	EMIM – $EtSO_4$	1.5 ml on a plate (ϕ =13 cm)	5.1	15.2	0.53 (Ar)	30 (RF)	-	-	9	RT	1.3 ± 0.7	[29]
Au	C_nMIM – BF_4 (n=2,4,8)<br>C_nMIM – OTf (n=2,4,6)<br>BMIM – FSA	2.0 cm^3 on 15.9 cm^2 stainless plate	5	2.5	12-14 (Ar)	-	1000	20	50	20 → 80	(SAXS): 0.5 → 3	[18]
Au	C_nMIM – BF_4 (n=2,4,8)	1 cm^3 on 6.1 cm^2 Teflon plane	5	2.5	10-15 (Ar)	-	1000	5	12 → 36	RT	(SAXS): 0.75 → 3.5	[30]

Table S1: Table with experimental parameters.

Au	BMIM – TFSI<br>BMIM – $N(CN)_2$<br>BMIM – BF_4<br>BMIM – PF_6	3.5 cm^3 in a PTFE container (ϕ =4 cm)	5	4	10 (Ar)	-	-	50	1	RT	<2.5	[31]<br>[32]
Au	PP13-TFSI	2.4 ml on a laboratory glass dish	5		3 (Ar)	-	-	20	60	RT	2.3±1.0	[33]
Au	BMIM – FAP	1.23 g in a cylindrical glass support (3 cm)	5	5	2.1 (Ar)	-	335	40	2.5	RT	4.9 ± 0.9	[13]
					OTHER IONIC LIQUIDS							
Ag	BMIM – $(Pf)_2N$<br>EMIM – TFSI<br>HMIM – TFSI<br>BuPy – TFSI<br>BmPyr – TFSI	multiple cavity holder (40 µL per cavity)	3.81		0.5 (Ar)	30	-	-	15	RT	7±4<br>7±3<br>7±3<br>4±3<br>9±8	[21]
Ag	(BCN)MIM – TFSI<br>(MeOE)MIM – TFSI<br>(HSE)MIM – TFSI	3 g into a Petri dish (ϕ=3 cm)	5	5	2 (Ar)	-	335	40	2.5	RT	5.0 (1.8-14.3)<br>8.2 (4.0-15.5)<br>8.7 (5.9-12.0)	[34]
Pt	[Me_3PrN, Et_3OctN, MePrPyr, MeBuPyr, EtMeIm, AllyEtIm] - TFSI	0.4 cm^3 on 2.5 x 2.5 cm glass plate		4.5	7 (Ar)	-	-	40	15	RT	1.4 - 2.4	[35]
Pt	HyEMIM – BF_4	0.80 cm^3 on 4.0 cm^2 glass plate	5	3.5	20 (Ar)	-	-	10	2.5	RT	1.0	[26]
Pd	(BCN)MIM – TFSI<br>(MeOE)MIM – TFSI<br>(HSE)MIM – TFSI	3 g into a Petri dish (ϕ=3 cm)	5	5	2 (Ar)	-	-	80 →<br>320	5	RT	1.4-4.6<br>1.3-6.4<br>2.4-7.3	[36]
In	AMIM – BF_4<br>AEI-BF_4	0.60 cm^3 on 10 cm^2 glass slide	4.9	2	2 (Ar)	-	-	10	10	RT	In@In_2O_3:18.2±4.0<br>In@In_2O_3:16.8±5.1	[5]
					PEEL							
**Target**	**Liquid**	**Amount of liquid substrate**	d_t, ***cm***	**WD, cm**	***p*, Pa**	***P*, W**	***V*, V**	***I*, mA**	t_s**, min**	T_s**, C**	d_{NP}**, nm**	**Ref.**
Cu	PEEL	7 g on a glass Petri plate (ϕ = 6 cm)			2 (Ar)	-	-	10 →<br>100	8 and 60	20	3 →10	[37]
Ti	PEEL	4.0 cm^3 into a cylindrical ceramic crucible (ϕ=4.5 cm)	5.1	15	0.7 (Ar)	20	-	-			TiO_2: 30-150	[25]
Ti	PEEL	2.5g on a Petri plate (5.7 cm^2)		6	2 (Ar)	-	310-265	400	60-180		(Ti→TiO_2): 2.5-5	[38]
Au	PEEL			4	10 (air)	-	-	40	3	RT	2.1±0.7	[39]
Au<br>Ag<br>Cu	PEEL	2.5 ml into a watch glass	7.62	13	0.5	Au-20<br>Ag-17<br>Cu-75	-	60<br>49<br>175	5	RT	Au -3<br>Ag-16±8<br>CuOx – 1.2±0.8	[40]
Au<br>Cu	PEEL	2.5 ml into a watch glass that was rotated (5 rpm)	7.62	13	0.5	17<br>75	-	49<br>175	5	RT	2.8<br>1.8 (CuOx)	[41]
					PEGs (mostly PEG with MW~600)							
**Target**	**Liquid**	**Amount of liquid substrate**	d_t, ***cm***	**WD, cm**	***p*, Pa**	***P*, W**	***V*, V**	***I*, mA**	t_s**, min**	T_s**, C**	d_{NP}**, nm**	**Ref.**

Table S1: Table with experimental parameters.

Au	PEG	2.0 cm^3 on a stainless plate 15.9 cm^2		2.5	16-19 (Ar)	-	1000	20	50	20 → 60	2 → 8	[42]
Au<br>Ag	PEG (200,400,600)	2 ml of PEG		4.5-6.0	8 (Ar)	-	-	10-60	100-600 sec		Up to 50	[43]
Au<br>Ag	PEG	10 cm^3 on a Petri plate (ϕ = 3.0 cm)	5	7.5	2 (Ar)	-	-	20<br>34	30	25	Au: 5.3±1.6<br>Ag: 5.2±1.7	[44]
Cu	PEG	10 cm^3 on a Petri plate (ϕ = 6.3 cm)		6	2 (Ar)	-	-	50	30	30	growth 3.1 → 4.1<br>Cu → CuO	[45]
Au	PEG	10 cm^3 on a Petri plate (ϕ = 6.3 cm)	5	11	2 (Ar)	-	-	10<br>20	30	30	2.6 ± 0.8<br>2.7 ± 0.9	[46]
					PEGs (mostly PEG with MW~600)							
Cu	PEG	10 cm^3 in 6.3cmϕ Petri plate	5	11	2 (Ar)	-	-	40<br>50	30	30	1.0± 0.3<br>1.5 ± 0.4	[46]
Pt	PEG	10 cm^3 on a Petri plate (ϕ = 6.3 cm)	5	5	2 (Ar)	-	-	5 →50	30	30	0.9 → 2.7	[47]
Au<br>Pt	PEG	2 ml on a Petri plate		5	8 (Ar)	-	-	30		RT	Au: 6.3<br>Pt: 3.9	[48]
Au	PEG-600	2 ml		5	8 (Ar)	-	-	30	1.67→8.33	20	About 10 nm	[49]
Ag	PEG<br>PEG (+MUA)		0.5	5	2 (Ar)	-	-	30	20	RT	7.4±3.6<br>2.2±0.5	[50]
Ag	PEG (+SMP)	10 g in a glass petri dish (ϕ = 6.5 cm)		5	2 (Ar)	-	-	30	20	RT	2.7±0.5	[51]
Au	PEG	10 g in a glass petri dish (ϕ = 6.5 cm)		5	2 (Ar)	-	-	20	60		7.4±2.1 without and 3.7±0.9 with stirring	[52]
Au	PEG+thiocholine	10 g in a glass petri dish (ϕ = 6.5 cm)		5	2 (Ar)	-	-	30	20	30	1.7 ± 0.6	[53]
Au	PEG<br>PEG-SH<br>PEG-S_2H_2	2 ml on a Petri plate (ϕ = 4 cm)		5	10 (Ar)	-	420-430	30	5	RT	5.6±1.8<br>4.2±0.8<br>3.6±0.6	[54]
Au	PEG<br>PEG-SH<br>PEG-NH_2	2 ml on a Petri plate (ϕ = 4 cm)		5	10 (Ar)	-	420-430	30	5	RT	5.6±1.9<br>1.5±0.3<br>2.5±0.8	[55]
Cu	PEG<br>PEG (+MUA)	7 g		6		-	-	20	60	40	2.6 ± 0.6<br>1.6 ± 0.3	[56]
Au<br>Ag	PEG (+MUTAB)	10.152 g of PEG + 0.04 g MUTAB			(Ar)	-	-	20<br>34	15		1.1 ± 0.3<br>1.4 ± 0.4	[57]
Au<br>Ag<br>Cu	PEG (+MUTAB)	3.384 g PEG + 0.02 g of MUTAB on a Petri plate (ϕ = 6.3 cm)		5	2 (Ar)	-	-	10	10		Au 1.2 ± 0.6<br>Ag 1.3 ± 0.6<br>Cu 1.0 ± 0.3	[58]
Au	PEG<br>PEG (+α-Thioglycerol)	10 cm^3 in 6.5 cm ϕ Petri plate (+0.05 g α-T)		2.5	2 -30 (air)	-	About 200	10→30	10→40	RT	no α-T: 4.7 - 5.5<br>α-T: 2.2-3.2	[59]
						GLYCEROL						
**Target**	**Liquid**	**Amount of liquid substrate**	***d_t*, cm**	**WD, cm**	***p*, Pa**	***P*, W**	***V*, V**	***I*, mA**	***t_s*, min**	***T_s*, C**	***d_{NP}*, nm**	**Ref.**
Au	Diglycerol (+thiocholine chloride)	12.8 g in 6.5cmϕ Petri plate		2.5	20 (Ar)	-	-	30	30	25	6.7±3.2<br>(2.0±0.7)	[60]

Table S1: Table with experimental parameters.

Au	Diglycerol<br>+SH<br>+NH_2<br>+C(O)OH	10 g on a Petri plate (ϕ = 6cm)		5	2 (Ar)	-	-	20	20	RT	6.7±3.2<br>4.6±0.9<br>3.0±0.8<br>2.1±0.8	[61]
Au	Glycerol		5.08	3.5	1.33 (Ar)	-	-	10	60, 120	RT	3.5 → 6.4	[62]
Pd<br>Pt	Glycerol	5 ml on a Petri plate (ϕ = 4cm)		5	4-6 (Ar)	-	420-430	40	5	20	Pd=2.4±0.4<br>Pt=1.7±0.3	[63]
Au<br>Ag	Glycerol	3 ml in 4cm ϕ Petri plate		5	7 (Ar)	-	-	40	5	20	4-12	[64]
GLYCEROL												
Au<br>Ag	Glycerol	2 ml on a Petri plate (ϕ = 4cm)		5	7 (Ar)	-	-	30	5	RT	3.5 ±1.5<br>3.5 ±2.4	[65]
Ag	Glycerol +1% of PVP	3 cm^3 on a Petri plate (ϕ = 4cm)			0.06 (Ar)	-	280 - 305	30	6	5 → 40	12.8 → 21.2	[66]
VEGETABLE OILS												
**Target**	**Liquid**	**Amount of liquid substrate**	***d_t*, *cm***	**WD, cm**	***p*, Pa**	***P*, W**	***V*, V**	***I*, mA**	***t_s*, min**	***T_s*, C**	***d_{NP}*, nm**	**Ref.**
Ag	Castor oil<br><br>Canola oil	1 cm^3 into a glass support (ϕ =3 cm)		5	2 (Ar)	13<br><br>38	320<br><br>420	-<br><br>-	2.5<br><br>2.5	RT	5.5±1.0<br><br>1.4 – 8.1	[67]
Au	Castor oil	1.15 cm^3 into a glass support (ϕ =3 cm)		5	2 (Ar)	13<br>13	330<br>330<br>260→450	-	2.5<br>10<br>2.5	RT	3.6 ± 1.0<br>3.8 ± 1.1<br>2.4 → 3.7	[68,69]
Ag	Castor oil	5 ml into plastic beaker (ϕ =2.8 cm)	5.1	20	0.067; 0.67; 2	20;40;60;80			1	RT	From 0.4 to 8 nm for primary NPs	[70]
Au	Castor oil	4 g into plastic beaker (ϕ =2.8 cm)	5.1	20	0.067; 0.67; 2	20;40;60;80			1→10	RT	2.4 – 3.2	[71]
Au	Sunflower oil	2 ml into a glass dish (ϕ =5 cm)		12	43	-	-	41	5<br>15<br>30	RT	2.9 ± 0.5<br>3.7 ± 0.7<br>3.4 ± 0.7	[72]
SILICONE OILS												
**Target**	**Liquid**	**Amount of liquid substrate**	***d_t*, *cm***	**WD, cm**	***p*, Pa**	***P*, W**	***V*, V**	***I*, mA**	***t_s*, min**	***T_s*, C**	***d_{NP}*, nm**	**Ref.**
Ag	Silicone oil	3 mm in diameter pure silicone oil drop on a piece of glass	81	6	0.2 (Ar)	15, 30, 50 (RF)	-	-		17 → 100	film	[73]
Ag	Silicone oil	2 mm in diameter pure silicone oil drop on a piece of glass		8	0.2 (Ar)	50 (RF)	-	-		RT	film	[74]
Ag<br>Fe	Silicone oils<br>Silicone oil +Koratine (80)	Running liquid substrate		8	1→30 (Ar)	Ag: 500<br>Fe:1000	-	-	Up to 240		Ag: 5-20	[75–77]
Al<br>Ag	Silicone oil	2 mm in diameter pure silicone oil drop on a piece of glass	81	6	0.2 (Ar)	-	-	30		18→120	film	[78]

Table S1: Table with experimental parameters.

Fe	Silicone oil	resulting oil substrate with an area of about 25×18 mm^2 thickness of about 0.5 mm	6	9	0.8 (Ar)	10 → 100	-	-	0.16→12		film	[79]
Fe	Silicone oil	resulting oil substrate with an area of about 25×18 mm^2 thickness of about 0.5 mm	6	9	0.8 (Ar)	10→120	-	-	0.08→12		film	[80]
Au	silicone oil<br>silicon oil +OTD<br>molten OTD	1.0 g on a Petri plate (ϕ = 3cm)		2.5	20 (Ar)	-	200	30	30	50	4.9±1.4<br>1.9±0.4<br>1.3±0.3	[81]

PEMP

**Target**	**Liquid**	**Amount of liquid substrate**	***d_t*, *cm***	**WD, cm**	***p*, Pa**	***P*, W**	***V*, V**	***I*, mA**	***t_s*, min**	***T_s*, C**	***d_{NP}*, nm**	**Ref.**
Cu	PEMP	7g			2 (Ar/air)	-	-	20	120	25	1.1 ± 0.2	[82]
Ag	PEMP	7 g on a glass Petri plate (ϕ =6 cm)		5	2 (Ar)	-	-	50	20	0 → 100	1.8-3.4	[83]
Ag	PEMP	13 g on a glass Petri plate (ϕ = 6cm)		4	10 (Ar)	-	-	30	20	RT	2.5	[84]
Au	PEMP			4	10 (air)	-	-	40	5 -15	RT	Less 1 nm	[39]

OTHER HOST LIQUIDS

**Target**	**Liquid**	**Amount of liquid substrate**	***d_t*, *cm***	**WD, cm**	***p*, Pa**	***P*, W**	***V*, V**	***I*, mA**	***t_s*, min**	***T_s*, C**	***d_{NP}*, nm**	**Ref.**
Au	MTAB	0.15 g of molten 6-MTAB in a 20 mm vessel		2.3	15 (air)	-	-	40	5 → 20	110	1.3 ± 0.3	[85]
Au	DES		5.7	3.5	5 (Ar)	-	450	20	0.5→5		5.0 ± 0.5	[86–88]
Au	Liquid crystals 4-pentyl-40-cyanobiphenyl	0.3 g in a cylindrical glass container (ϕ=15mm)			20 (oxygen)	-	1000	-	20	RT	2.9±0.6	[89]

References

(1) Torimoto, T.; Okazaki, K. I.; Kiyama, T.; Hirahara, K.; Tanaka, N.; Kuwabata, S. *Appl. Phys. Lett.* **2006**, *89*, 243117. doi:10.1063/1.2404975

(2) Sugioka, D.; Kameyama, T.; Kuwabata, S.; Torimoto, T. *Phys. Chem. Chem. Phys.* **2015**, *17*, 13150–13159. doi:10.1039/c5cp01602a

(3) Torimoto, T.; Ohta, Y.; Enokida, K.; Sugioka, D.; Kameyama, T.; Yamamoto, T.; Shibayama, T.; Yoshii, K.; Tsuda, T.; Kuwabata, S. *J. Mater. Chem. A* **2015**, *3*, 6177–6186. doi:10.1039/C4TA06643J

(4) Suzuki, T.; Suzuki, S.; Tomita, Y.; Okazaki, K. I.; Shibayama, T.; Kuwabata, S.; Torimoto, T. *Chem. Lett.* **2010**, *39*, 1072–1074. doi:10.1246/cl.2010.1072

(5) Suzuki, T.; Okazaki, K.; Suzuki, S.; Shibayama, T.; Kuwabata, S.; Torimoto, T. *Chem. Mater.* **2010**, *22*, 5209–5215. doi:10.1021/cm101164r

(6) Okazaki, K. I.; Kiyama, T.; Suzuki, T.; Kuwabata, S.; Torimoto, T. *Chem. Lett.* **2009**, *38*, 330–331. doi:10.1246/cl.2009.330

(7) Suzuki, S.; Suzuki, T.; Tomita, Y.; Hirano, M.; Okazaki, K.; Kuwabata, S.; Torimoto, T. *CrystEngComm* **2012**, *14*, 4922. doi:10.1039/c2ce25235j

(8) Tsuda, T.; KURIHARA, T.; HOSHINO, Y.; KIYAMA, T.; OKAZAKI, K.; TORIMOTO, T.; KUWABATA, S. *Electrochemistry* **2009**, *77*, 693–695. doi:10.5796/electrochemistry.77.693

(9) Tsuda, T.; Yoshii, K.; Torimoto, T.; Kuwabata, S. *J. Power Sources* **2010**, *195*, 5980–5985. doi:10.1016/j.jpowsour.2009.11.027

(10) Suzuki, S.; Ohta, Y.; Kurimoto, T.; Kuwabata, S.; Torimoto, T. *Phys. Chem. Chem. Phys.* **2011**, *13*, 13585–13593. doi:10.1039/c1cp20814d

(11) Hatakeyama, Y.; Takahashi, S.; Nishikawa, K. *J. Phys. Chem. C* **2010**, *114*, 11098–11102. doi:10.1021/jp102763n

(12) Hatakeyama, Y.; Onishi, K.; Nishikawa, K. *RSC Adv.* **2011**, *1*, 1815–1821. doi:10.1039/c1ra00688f

(13) Wender, H.; De Oliveira, L. F.; Migowski, P.; Feil, A. F.; Lissner, E.; Prechtl, M. H. G.; Teixeira, S. R.; Dupont, J. *J. Phys. Chem. C* **2010**, *114*, 11764–11768. doi:10.1021/jp102231x

(14) Khatri, O. P.; Adachi, K.; Murase, K.; Okazaki, K. I.; Torimoto, T.; Tanaka, N.; Kuwabata, S.; Sugimura, H. *Langmuir* **2008**, *24*, 7785–7792. doi:10.1021/la800678m

(15) Okazaki, K. I.; Sakuma, J.; Yasui, J. I.; Kuwabata, S.; Hirahara, K.; Tanaka, N.; Torimoto, T. *Chem. Lett.* **2011**, *40*, 84–86. doi:10.1246/cl.2011.84

(16) Kameyama, T.; Ohno, Y.; Kurimoto, T.; Okazaki, K.; Uematsu, T.; Kuwabata, S.; Torimoto, T. *Phys. Chem. Chem. Phys.* **2010**, *12*, 1804–1811. doi:10.1039/B914230D

(17) Okazaki, K.; Kiyama, T.; Hirahara, K.; Tanaka, N.; Kuwabata, S.; Torimoto, T. *Chem. Commun.* **2008**, No. 6, 691–693. doi:10.1039/B714761A

(18) Hatakeyama, Y.; Judai, K.; Onishi, K.; Takahashi, S.; Kimura, S.; Nishikawa, K. *Phys. Chem. Chem. Phys.* **2016**, *18*, 2339–2349. doi:10.1039/c5cp04123f

(19) Iimori, T.; Hatakeyama, Y.; Nishikawa, K.; Kato, M.; Ohta, N. *Chem. Phys. Lett.* **2013**, *586*, 100–103. doi:10.1016/j.cplett.2013.09.010

(20) SUZUKI, T.; OKAZAKI, K.; KIYAMA, T.; KUWABATA, S.; TORIMOTO, T. *Electrochemistry* **2009**, *77*, 636–638. doi:10.5796/electrochemistry.77.636

(21) Meyer, H.; Meischein, M.; Ludwig, A. *ACS Comb. Sci.* **2018**, *20*, 243–250. doi:10.1021/acscombsci.8b00017

(22) Oda, Y.; Hirano, K.; Yoshii, K.; Kuwabata, S.; Torimoto, T.; Miura, M. *Chem. Lett.* **2010**, *39*, 1069–1071. doi:10.1246/cl.2010.1069

(23) Meischein, M.; Garzón-Manjón, A.; Frohn, T.; Meyer, H.; Salomon, S.; Scheu, C.; Ludwig, A. *ACS Comb. Sci.* **2019**. doi:10.1021/acscombsci.9b00140

(24) Meischein, M.; Ludwig, A. *J. Nanoparticle Res.* **2021**, *23*, 1–15. doi:10.1007/s11051-021-05248-8

(25) Carette, X.; Debièvre, B.; Cornil, D.; Cornil, J.; Leclère, P.; Maes, B.; Gautier, N.; Gautron, E.; El Mel, A. A.; Raquez, J. M.; Konstantinidis, S. *J. Phys. Chem. C* **2018**, *122*, 26605–26612. doi:10.1021/acs.jpcc.8b06987

(26) Sugioka, D.; Kameyama, T.; Kuwabata, S.; Yamamoto, T.; Torimoto, T. *ACS Appl. Mater. Interfaces* **2016**, *8*, 10874–10883. doi:10.1021/acsami.6b01978

(27) Wender, H.; Teixeira, S. R.; Migowski, P.; Feil, A. F.; Dupont, J.; Prechtl, M. H. G.; Machado, G.; de Oliveira, L. F.; Leal, R. *Phys. Chem. Chem. Phys.* **2011**, *13*, 13552. doi:10.1039/c1cp21406c

(28) Calabria, L.; Fernandes, J. A.; Migowski, P.; Bernardi, F.; Baptista, D. L.; Leal, R.; Grehl, T.; Dupont, J. *Nanoscale* **2017**, *9*, 18753–18758. doi:10.1039/C7NR06167F

(29) Hamm, S. C.; Basuray, S.; Mukherjee, S.; Sengupta, S.; Mathai, J. C.; Baker, G. A.; Gangopadhyay, S. *J. Mater. Chem. A* **2014**, *2*, 792–803. doi:10.1039/C3TA13431H

(30) Hatakeyama, Y.; Okamoto, M.; Torimoto, T.; Kuwabata, S.; Nishikawa, K. *J. Phys. Chem. C* **2009**, *113*, 3917–3922. doi:10.1021/jp807046u

(31) Vanecht, E.; Binnemans, K.; Patskovsky, S.; Meunier, M.; Seo, J. W.; Stappers, L.; Fransaer, J. *Phys. Chem. Chem. Phys.* **2012**, *14*, 5662. doi:10.1039/c2cp23677j

(32) Vanecht, E.; Binnemans, K.; Seo, J. W.; Stappers, L.; Fransaer, J. *Phys. Chem. Chem. Phys.* **2011**, *13*, 13565–13571. doi:10.1039/c1cp20552h

(33) Yoda, M.; Takashima, T.; Akiyoshi, K.; Torimoto, T.; Irie, H. *J. Chem. Phys.* **2020**, *153*, 014701. doi:10.1063/5.0010100

(34) Qadir, M. I.; Kauling, A.; Calabria, L.; Grehl, T.; Dupont, J. *Nano-Structures and Nano-Objects* **2018**, *14*, 92–97. doi:10.1016/j.nanoso.2018.01.015

(35) Yoshii, K.; Tsuda, T.; Arimura, T.; Imanishi, A.; Torimoto, T.; Kuwabata, S. *RSC Adv.* **2012**, *2*, 8262–8264. doi:10.1039/c2ra21243a

(36) Qadir, M. I.; Kauling, A.; Ebeling, G.; Fartmann, M.; Grehl, T.; Dupont, J. *Aust. J. Chem.* **2019**, *72*, 49. doi:10.1071/CH18183

(37) Nakagawa, K.; Narushima, T.; Udagawa, S.; Yonezawa, T. *J. Phys. Conf. Ser.* **2013**, *417*. doi:10.1088/1742-6596/417/1/012038

(38) Porta, M.; Nguyen, M. T.; Yonezawa, T.; Tokunaga, T.; Ishida, Y.; Tsukamoto, H.; Shishino, Y.; Hatakeyama, Y. *New J. Chem.* **2016**, *40*, 9337–9343. doi:10.1039/c6nj01624c

(39) Shishino, Y.; Yonezawa, T.; Udagawa, S.; Hase, K.; Nishihara, H. *Angew. Chemie* **2011**, *123*, 729–731. doi:10.1002/ange.201005723

(40) Chauvin, A.; Sergievskaya, A.; Fucikova, A.; Antunes Corrêa, C.; Veselý, J.; Cornil, J.; Cornil, D.; Dopita, M.; Konstantinidis, S. *Nanoscale Adv.* **2021**. doi:10.1039/D1NA00222H

(41) Chauvin, A.; Sergievskaya, A.; El Mel, A.-A.; Fucikova, A.; Antunes Corrêa, C.; Vesely, J.; Duverger-Nédellec, E.; Cornil, D.; Cornil, J.; Tessier, P.-Y.; Dopita, M.; Konstantinidis, S. *Nanotechnology* **2020**, *31*, 455303. doi:10.1088/1361-6528/abaa75

(42) Hatakeyama, Y.; Morita, T.; Takahashi, S.; Onishi, K.; Nishikawa, K. *J. Phys. Chem. C* **2011**, *115*, 3279–3285. doi:10.1021/jp110455k

(43) Slepička, P.; Elashnikov, R.; Ulbrich, P.; Staszek, M.; Kolská, Z.; Švorčík, V. *J. Nanoparticle Res.* **2015**, *17*. doi:10.1007/s11051-014-2850-z

(44) Nguyen, M. T.; Yonezawa, T.; Wang, Y.; Tokunaga, T. *Mater. Lett.* **2016**, *171*, 75–78. doi:10.1016/j.matlet.2016.02.047

(45) Chau, Y. R.; Deng, L.; Nguyen, M. T.; Yonezawa, T. *MRS Adv.* **2019**, *4*, 305–309. doi:10.1557/adv.2019.55

(46) Nguyen, M. T.; Zhang, H.; Deng, L.; Tokunaga, T.; Yonezawa, T. *Langmuir* **2017**, *33*, 12389–12397. doi:10.1021/acs.langmuir.7b03194

(47) Deng, L.; Nguyen, M. T.; Yonezawa, T. *Langmuir* **2018**, *34*, 2876–2881. doi:10.1021/acs.langmuir.7b04274

(48) Pišlová, M.; Kalbáčová, M. H.; Vrabcová, L.; Slepička, P.; Kolská, Z.; Švorčík, V. *Dig. J. Nanomater. Biostructures* **2018**, *13*, 1035–1044

(49) Fajstavr, D.; Karasová, A.; Michalcová, A.; Ulbrich, P.; Kasálková, N. S.; Siegel, J.; Švorčík, V.; Slepička, P. *Nanomaterials* **2021**, *11*, 1–17. doi:10.3390/nano11061434

(50) Ishida, Y.; Nakabayashi, R.; Matsubara, M.; Yonezawa, T. *New J. Chem.* **2015**, *39*, 4227–4230. doi:10.1039/c5nj00294j

(51) Ishida, Y.; Nakabayashi, R.; Corpuz, R. D.; Yonezawa, T. *Colloids Surfaces A Physicochem. Eng. Asp.* **2017**, *518*, 25–29. doi:10.1016/j.colsurfa.2017.01.022

(52) Ishida, Y.; Akita, I.; Sumi, T.; Matsubara, M.; Yonezawa, T. *Sci. Rep.* **2016**, *6*, 1–14. doi:10.1038/srep29928

(53) Ishida, Y.; Morita, A.; Tokunaga, T.; Yonezawa, T. *Langmuir* **2018**, *34*, 4024–4030. doi:10.1021/acs.langmuir.8b00067

(54) Rezníčková, A.; Slepicka, P.; Slavikova, N.; Staszek, M.; Svorcik, V. *Colloids Surfaces A Physicochem. Eng. Asp.* **2017**, *523*, 91–97. doi:10.1016/j.colsurfa.2017.04.005

(55) Rezníčková, A.; Slavikova, N.; Kolska, Z.; Kolarova, K.; Belinova, T.; Hubalek Kalbacova, M.; Cieslar, M.; Svorcik, V. *Colloids Surfaces A Physicochem. Eng. Asp.* **2019**, *560*, 26–34. doi:10.1016/j.colsurfa.2018.09.083

(56) Porta, M.; Nguyen, M. T.; Ishida, Y.; Yonezawa, T. *RSC Adv.* **2016**, *6*, 105030–105034. doi:10.1039/c6ra17291a

(57) Corpuz, R. D.; Ishida, Y.; Nguyen, M. T.; Yonezawa, T. *Langmuir* **2017**, *33*, 9144–9150. doi:10.1021/acs.langmuir.7b02011

(58) Corpuz, R. D.; Ishida, Y.; Yonezawa, T. *New J. Chem.* **2017**, *41*, 6828–6833. doi:10.1039/c7nj01369h

(59) Sumi, T.; Motono, S.; Ishida, Y.; Shirahata, N.; Yonezawa, T. *Langmuir* **2015**, *31*, 4323–4329. doi:10.1021/acs.langmuir.5b00294

(60) Ishida, Y.; Lee, C.; Yonezawa, T. *Sci. Rep.* **2015**, *5*, 8–13. doi:10.1038/srep15372

(61) Akita, I.; Ishida, Y.; Yonezawa, T. *Bull. Chem. Soc. Jpn.* **2016**, *89*, 1054–1056. doi:10.1246/bcsj.20160187

(62) Lee, S. H.; Jung, H. K.; Kim, T. C.; Kim, C. H.; Shin, C. H.; Yoon, T.-S.; Hong, A.-R.; Jang, H. S.; Kim, D. H. *Appl. Surf. Sci.* **2018**, *434*, 1001–1006. doi:10.1016/j.apsusc.2017.11.008

(63) Staszek, M.; Siegel, J.; Kolářová, K.; Rimpelová, S.; Švorčík, V. *Micro Nano Lett.* **2014**, *9*, 778–781. doi:10.1049/mnl.2014.0345

(64) Siegel, J.; Kolářová, K.; Vosmanská, V.; Rimpelová, S.; Leitner, J.; Švorčík, V. *Mater. Lett.* **2013**, *113*, 59–62. doi:10.1016/j.matlet.2013.09.047

(65) Siegel, J.; Kvítek, O.; Ulbrich, P.; Kolská, Z.; Slepička, P.; Švorčík, V. *Mater. Lett.* **2012**, *89*, 47–50. doi:10.1016/j.matlet.2012.08.048

(66) Staszek, M.; Siegel, J.; Polívková, M.; Švorčík, V. *Mater. Lett.* **2017**, *186*, 341–344. doi:10.1016/j.matlet.2016.10.036

(67) Wender, H.; Gonçalves, R. V.; Feil, A. F.; Migowski, P.; Poletto, F. S.; Pohlmann, A. R.; Dupont, J.; Teixeira, S. R. *J. Phys. Chem. C* **2011**, *115*, 16362–16367. doi:10.1021/jp205390d

(68) Wender, H.; De Oliveira, L. F.; Feil, A. F.; Lissner, E.; Migowski, P.; Meneghetti, M. R.; Teixeira, S. R.; Dupont, J. *Chem. Commun.* **2010**, *46*, 7019–7021. doi:10.1039/c0cc01353f

(69) Castro, H. P. S.; Wender, H.; Alencar, M. A. R. C.; Teixeira, S. R.; Dupont, J.; Hickmann, J. M. *J. Appl. Phys.* **2013**, *114*, 183104. doi:10.1063/1.4831679

(70) Sergievskaya, A.; O'Reilly, A.; Chauvin, A.; Veselý, J.; Panepinto, A.; De Winter, J.; Cornil, D.; Cornil, J.; Konstantinidis, S. *Colloids Surfaces A Physicochem. Eng. Asp.* **2021**, *615*, 126286. doi:10.1016/j.colsurfa.2021.126286

(71) Sergievskaya, A.; O'Reilly, A.; Alem, H.; De Winter, J.; Cornil, D.; Cornil, J.; Konstantinidis, S. *Front. Nanotechnol.* **2021**, *3*. doi:10.3389/fnano.2021.710612

(72) Michels, F. S.; Gonçalves, P. J.; Nascimento, V. A.; Oliveira, S. L.; Wender, H.; Caires, A. R. L. *Nanomaterials* **2021**, *11*, 1668. doi:10.3390/nano11071668

(73) Feng, C.-M.; Ge, H. L.; Tong, M.-R.; Ye, G. X.; Jiao, Z.-K. *Thin Solid Films* **1999**, *342*, 30–34. doi:10.1016/S0040-6090(98)01151-1

(74) Ye, G. X.; Feng, C. M.; Zhang, Q. R.; Ge, H. L.; Zhang, X. J. *Chinese Phys. Lett.* **1996**, *13*, 772–774. doi:10.1088/0256-307X/13/10/016

(75) Wagener, M.; Günther, B. High Pressure DC-Magnetron Sputtering on Liquids: A New Process for the Production of Metal Nanosuspensions. In *Structure, Dynamics and Properties of Disperse Colloidal Systems*; Steinkopff: Darmstadt, 1998; Vol. 111, pp 78–81

(76) Wagener, M.; Murty, B. S.; Günther, B. *MRS Proc.* **1996**, *457*, 149. doi:10.1557/PROC-457-149

(77) Wagener, M.; Günther, B. *J. Magn. Magn. Mater.* **1999**, *201*, 41–44. doi:10.1016/S0304-8853(99)00055-4

(78) Ge, H. L.; Feng, C.; Ye, G. X.; Ren, Y.; Jiao, Z. *J. Appl. Phys.* **1997**, *82*, 5469–5471. doi:10.1063/1.365574

(79) Zhang, Y. J.; Yu, S. J. *Int. J. Mod. Phys. B* **2009**, *23*, 3147–3157. doi:10.1142/S0217979209049772

(80) YU, S.-J.; Zhang, Y. J.; CHEN, J.-X.; Ge, H. L. *Surf. Rev. Lett.* **2006**, *13*, 779–784. doi:10.1142/S0218625X06008840

(81) Ishida, Y.; Sumi, T.; Yonezawa, T. *New J. Chem.* **2015**, *39*, 5895–5897. doi:10.1039/c5nj01011j

(82) Porta, M.; Nguyen, M. T.; Tokunaga, T.; Ishida, Y.; Liu, W.-R.; Yonezawa, T. *Langmuir* **2016**, *32*, 12159–12165. doi:10.1021/acs.langmuir.6b03017

(83) Ishida, Y.; Udagawa, S.; Yonezawa, T. *Colloids Surfaces A Physicochem. Eng. Asp.* **2016**, *498*, 106–111. doi:10.1016/j.colsurfa.2016.03.044

(84) Ishida, Y.; Udagawa, S.; Yonezawa, T. *Colloids Surfaces A Physicochem. Eng. Asp.* **2016**, *504*, 437–441. doi:10.1016/j.colsurfa.2016.05.035

(85) Shishino, Y.; Yonezawa, T.; Kawai, K.; Nishihara, H. *Chem. Commun.* **2010**, *46*, 7211. doi:10.1039/c0cc01702g

(86) Raghuwanshi, V. S.; Ochmann, M.; Hoell, A.; Polzer, F.; Rademann, K. *Langmuir* **2014**, *30*, 6038–6046. doi:10.1021/la500979p

(87) Raghuwanshi, V. S.; Ochmann, M.; Polzer, F.; Hoell, A.; Rademann, K. *Chem. Commun.* **2014**, *50*, 8693–8696. doi:10.1039/c4cc02588a

(88) O’Neill, M.; Raghuwanshi, V. S.; Wendt, R.; Wollgarten, M.; Hoell, A.; Rademann, K. *Zeitschrift für Phys. Chemie* **2015**, *229*, 221–234. doi:10.1515/zpch-2014-0644

(89) Yoshida, H.; Kawamoto, K.; Kubo, H.; Tsuda, T.; Fujii, A.; Kuwabata, S.; Ozaki, M. *Adv. Mater.* **2010**, *22*, 622–626. doi:10.1002/adma.200902831
