# Supplementary material for: Sputtering onto liquids: a critical review
Source: Beilstein J Nanotechnol. 2022 Jan 4;13:10–53. doi: 10.3762/bjnano.13.2 (PMC8744456; doi:10.3762/bjnano.13.2)
Supplement: File 2 — Comparison the sizes of the metal NPs prepared by magnetron sputtering onto similar host liquids. [file Beilstein_J_Nanotechnol-13-10-s002.pdf]

BEILSTEIN JOURNAL OF NANOTECHNOLOGY

Supporting Information

for

Sputtering onto liquids: a critical review

Anastasiya Sergievskaya, Adrien Chauvin and Stephanos Konstantinidis

Beilstein J. Nanotechnol. **2022,** *13,* 10–53. doi:10.3762/bjnano.13.2

Comparison the sizes of the metal NPs prepared by magnetron sputtering onto similar host liquids

License and Terms: This is a supporting information file under the terms of the Creative Commons Attribution License (https://creativecommons.org/licenses/by/4.0). Please note that the reuse, redistribution and reproduction in particular requires that the author(s) and source are credited and that individual graphics may be subject to special legal provisions.
The license is subject to the *Beilstein Journal of Nanotechnology* terms and conditions: (https://www.beilstein-journals.org/bjnano/terms)

Depositions were done in the sputter coaters with 2-inch metal targets, at temperatures of 20–25 °C. Parameters: *p*-pressure (Pa), *WD* – working distance (mm), *V*- voltage (V), *I* -current (mA), t_s – duration of deposition (min), NP size – diameter of NPs determined by TEM (nm).

Table S1: Sputtering onto ionic liquid TMPA-TFSI.

Target	Amount of liquid substrate	*p*, Pa	*WD*, mm	*V*, V	*I*, mA	t_s, min	NP size (nm)	Ref.
Pt	0.4 cm^3 of IL on a glass plate (2.5 x 2.5 cm)	7	45		40	5	2.24±0.36	[1]
Pt	0.2 cm^3 of IL on a glass plate (2.5 x 2.5 cm)	7	45		40	5 → 30	2.3-2.4	[2]
Pt<br>AuPt<br>Au	IL was spread on a glass plate 10 cm^2	10	20	500	40	5	1.0 ± 0.3<br>1.5 ± 0.4<br>2.9 ± 0.7	[3]
Au	0.8 cm^3 of IL on a glass plate 4 cm^2	20	35		10	5	2.2±0.4	[4]
Au	0.6 cm^3 of IL on a glass plate 10 cm^2	20	35		40	5	2.3±0.3	[5]

Table S2: Sputtering onto ionic liquid BMIM-PF_6.

Target	Amount of liquid substrate	*p*, Pa	*WD*, mm	*P, W*	*V*, V	*I*, mA	t_s, min	NP size (nm)	Ref.
Au	0.6 cm^3 of IL on a glass plate 10 cm^2	20	35			40	5	2.6 ± 0.3	[6]
Au	0.6 cm^3 of IL on a glass (26 x 38x 1mm3)	20	30			40	5	2.3 ± 0.4	[7]
Au	0.60 cm3 of IL on a glass slide (5.7 cm^2)	20	35			40	5	2.5	[8]
Au	0.60 cm3 of IL on a glass slide (10 cm^2)	20	35			40	5	2.6±0.6	[9]
Au	2.0 cm^3 of IL on a stainless plate 15.9 cm^2	16-19	25		1000	20	50	1.0–1.5	[10]
Au	1.0 cm^3 of IL on a Teflon plate 6.1 cm^2	20	35			40	5	2.6±0.3	[11]
Ag	1.0 cm^3 of IL on a Teflon plate 6.1 cm^2	20	35			40	5	6±1.5	[11]
Ag	multiple cavity holder (40 µL per cavity)	0.5		30			15	6±3	[12]
Ag	0.6 cm^3 of IL on a glass plate 10 cm^2	5<br>5	85<br>85			10<br>40	5<br>5	5.7±1.8<br>11	[13]
Pd	0.2 cm^3 of IL on a glass plate (2.5 x 2.5 cm)	8	45			40	5	3.0	[14]

Table S3: Sputtering onto ionic liquid BMIM-TFSI.

Target	Amount of liquid substrate	*p*, Pa	*WD*, mm	*P, W*	*V*, V	*I*, mA	t_s, min	NP size (nm)	Ref.
Pd	0.2 cm^3 of IL on a glass plate (2.5 x 2.5 cm)	8	45			40	5	2.2	[14]
Au	0.6 cm^3 of IL on a glass (26 x 38x 1 mm3)	20	30			40	5	2.0 ± 0.4	[7]
Au	3.5 cm^3 of IL was poured in a PTFE container (ϕ =4 cm)	10	40			50	1	5-6	[15]
Ag	multiple cavity holder (40 µL per cavity)	0.5		30			15	8±4	[12]
Ag	4 cm^3 of IL into a cylindrical ceramic crucible (ϕ =4.5 cm)	1.3	150	20			20	5-20	[16]

Table S4: Sputtering onto pure PEG-600.

Target	Amount of liquid substrate	*p*, Pa	*WD*, mm	*V*, V	*I*, mA	t_s, min	NP size (nm)	Ref.
Pt	2 ml on a Petri plate	8	50		30		3.9	[17]
Au	2 ml on a Petri plate	8	50		30		6.3	[17]
Au	2 ml on a Petri plate (ϕ = 4 cm)	10	50	420-430	30	5 and 15	5.6±1.9	[18]
Au	2 ml on a Petri plate (ϕ = 4 cm)	10	50	420-430	30	5	5.6±1.8	[19]
Ag		2	50		30	20	7.4±3.6	[20]

Table S5: Sputtering onto pure glycerol.

Target	Amount of liquid substrate	*p*, Pa	*WD*, mm	*V*, V	*I*, mA	t_s, min	NP size (nm)	Ref.
Pt	5 ml on a Petri plate (ϕ = 4cm)	4-6	50	420	40	5	1.7±0.3	[21]
Pd	5 ml on a Petri plate (ϕ = 4cm)	4-6	50	420	40	5	2.4±0.4	[21]
Au	2 ml on a Petri plate (ϕ = 4cm)	7	50		30	5	3.5 ±1.5	[22]
Ag	2 ml on a Petri plate (ϕ = 4cm)	7	50		30	5	3.5 ±2.4	[22]

References

(1) Tsuda, T.; Kurihara, T.; Hoshino, Y.; Kiyama, T.; Okazaki, K.; Torimoto, T.; Kuwabata, S. *Electrochemistry* **2009**, *77*, 693–695. doi:10.5796/electrochemistry.77.693

(2) Tsuda, T.; Yoshii, K.; Torimoto, T.; Kuwabata, S. *J. Power Sources* **2010**, *195*, 5980–5985. doi:10.1016/j.jpowsour.2009.11.027

(3) Suzuki, S.; Suzuki, T.; Tomita, Y.; Hirano, M.; Okazaki, K.; Kuwabata, S.; Torimoto, T. *CrystEngComm* **2012**, *14*, 4922. doi:10.1039/c2ce25235j

(4) Sugioka, D.; Kameyama, T.; Kuwabata, S.; Torimoto, T. *Phys. Chem. Chem. Phys.* **2015**, *17*, 13150–13159. doi:10.1039/c5cp01602a

(5) Okazaki, K. I.; Kiyama, T.; Suzuki, T.; Kuwabata, S.; Torimoto, T. *Chem. Lett.* **2009**, *38*, 330–331. doi:10.1246/cl.2009.330

(6) Khatri, O. P.; Adachi, K.; Murase, K.; Okazaki, K. I.; Torimoto, T.; Tanaka, N.; Kuwabata, S.; Sugimura, H. *Langmuir* **2008**, *24*, 7785–7792. doi:10.1021/la800678m

(7) Suzuki, S.; Ohta, Y.; Kurimoto, T.; Kuwabata, S.; Torimoto, T. *Phys. Chem. Chem. Phys.* **2011**, *13*, 13585–13593. doi:10.1039/c1cp20814d

(8) Okazaki, K. I.; Sakuma, J.; Yasui, J. I.; Kuwabata, S.; Hirahara, K.; Tanaka, N.; Torimoto, T. *Chem. Lett.* **2011**, *40*, 84–86. doi:10.1246/cl.2011.84

(9) Kameyama, T.; Ohno, Y.; Kurimoto, T.; Okazaki, K.; Uematsu, T.; Kuwabata, S.; Torimoto, T. *Phys. Chem. Chem. Phys.* **2010**, *12*, 1804–1811. doi:10.1039/B914230D

(10) Iimori, T.; Hatakeyama, Y.; Nishikawa, K.; Kato, M.; Ohta, N. *Chem. Phys. Lett.* **2013**, *586*, 100–103. doi:10.1016/j.cplett.2013.09.010

(11) Okazaki, K.; Kiyama, T.; Hirahara, K.; Tanaka, N.; Kuwabata, S.; Torimoto, T. *Chem. Commun.* **2008**, No. 6, 691–693. doi:10.1039/B714761A

(12) Meyer, H.; Meischein, M.; Ludwig, A. *ACS Comb. Sci.* **2018**, *20*, 243–250. doi:10.1021/acscombsci.8b00017

(13) SUZUKI, T.; OKAZAKI, K.; KIYAMA, T.; KUWABATA, S.; TORIMOTO, T. *Electrochemistry* **2009**, *77*, 636–638. doi:10.5796/electrochemistry.77.636

(14) Oda, Y.; Hirano, K.; Yoshii, K.; Kuwabata, S.; Torimoto, T.; Miura, M. *Chem. Lett.* **2010**, *39*, 1069–1071. doi:10.1246/cl.2010.1069

(15) Vanecht, E.; Binnemans, K.; Patskovsky, S.; Meunier, M.; Seo, J. W.; Stappers, L.; Fransaer, J. *Phys. Chem. Chem. Phys.* **2012**, *14*, 5662. doi:10.1039/c2cp23677j

(16) Carette, X.; Debièvre, B.; Cornil, D.; Cornil, J.; Leclère, P.; Maes, B.; Gautier, N.; Gautron, E.; El Mel, A. A.; Raquez, J. M.; Konstantinidis, S. *J. Phys. Chem. C* **2018**, *122*, 26605–26612. doi:10.1021/acs.jpcc.8b06987

(17) Pišlová, M.; Kalbáčová, M. H.; Vrabcová, L.; Slepička, P.; Kolská, Z.; Švorčík, V. *Dig. J. Nanomater. Biostructures* **2018**, *13*, 1035–1044

(18) Rezníčková, A.; Slavikova, N.; Kolska, Z.; Kolarova, K.; Belinova, T.; Hubalek Kalbacova, M.; Cieslar, M.; Svorcik, V. *Colloids Surfaces A Physicochem. Eng. Asp.* **2019**, *560*, 26–34. doi:10.1016/j.colsurfa.2018.09.083

(19) Rezníčková, A.; Slepicka, P.; Slavikova, N.; Staszek, M.; Svorcik, V. *Colloids Surfaces A Physicochem. Eng. Asp.* **2017**, *523*, 91–97. doi:10.1016/j.colsurfa.2017.04.005

(20) Ishida, Y.; Nakabayashi, R.; Matsubara, M.; Yonezawa, T. *New J. Chem.* **2015**, *39*, 4227–4230. doi:10.1039/c5nj00294j

(21) Staszek, M.; Siegel, J.; Kolářová, K.; Rimpelová, S.; Švorčík, V. *Micro Nano Lett.* **2014**, *9*, 778–781. doi:10.1049/mnl.2014.0345

(22) Siegel, J.; Kvítek, O.; Ulbrich, P.; Kolská, Z.; Slepička, P.; Švorčík, V. *Mater. Lett.* **2012**, *89*, 47–50. doi:10.1016/j.matlet.2012.08.048
